# Enhancement of Environmental Hazard Degradation in the Presence of Lignin: a Proteomics Study

**DOI:** 10.1038/s41598-017-10132-4

**Published:** 2017-09-12

**Authors:** Su Sun, Shangxian Xie, Yanbing Cheng, Hongbo Yu, Honglu Zhao, Muzi Li, Xiaotong Li, Xiaoyu Zhang, Joshua S. Yuan, Susie Y. Dai

**Affiliations:** 1 School of Life Science and Technology, Huazhong University of Science and Technology, Wuhan, China; 2 Department of Veterinary Pathology, Texas A&M University, College station, TX, USA; 3 Synthetic and Systems Biology Innovation Hub, Department of Plant Pathology and Microbiology, Texas A&M University, College Station, TX USA; 4 Office of Texas State Chemist, Texas A&M University, College Station, TX USA; 5 Present Address: State Hygie nic Laboratory, University of Iowa, Coralville, IA 52246 USA

## Abstract

Proteomics studies of fungal systems have progressed dramatically based on the availability of more fungal genome sequences in recent years. Different proteomics strategies have been applied toward characterization of fungal proteome and revealed important gene functions and proteome dynamics. Presented here is the application of shot-gun proteomic technology to study the bio-remediation of environmental hazards by white-rot fungus. Lignin, a naturally abundant component of the plant biomass, is discovered to promote the degradation of Azo dye by white-rot fungus *Irpex lacteus* CD2 in the lignin/dye/fungus system. Shotgun proteomics technique was used to understand degradation mechanism at the protein level for the lignin/dye/fungus system. Our proteomics study can identify about two thousand proteins (one third of the predicted white-rot fungal proteome) in a single experiment, as one of the most powerful proteomics platforms to study the fungal system to date. The study shows a significant enrichment of oxidoreduction functional category under the dye/lignin combined treatment. An *in vitro* validation is performed and supports our hypothesis that the synergy of Fenton reaction and manganese peroxidase might play an important role in DR5B dye degradation. The results could guide the development of effective bioremediation strategies and efficient lignocellulosic biomass conversion.

## Introduction

Fungi represents an important class of eukaryotic organisms that are ubiquitously present in the natural ecosystem. Particularly, many fungal species are of great economic value and commercial relevance due to the applications in the food, healthcare, and biotechnology industry. There is an emerging new branch of biotechnology known as “white biotechnology”, in which living cells and enzymes have been used to produce degradable products that are more environment friendly. Fungi and their versatile enzymes have been of great focus in the white biotechnology applications^1^. Furthermore, fungal treatment has presented as a viable resolution for environmental bioremediation as fungi can effectively degrade a wide range of pollutants^2^. At the same time, the capability to efficiently decompose lignocellulose materials by fungi has been widely studied in the biofuel researches. Among the various species in the fungi kingdom, white rot fungus has been used for industrial dye treatment and biomass decomposition. White-rot fungus can degrade various xenobiotic compounds, dyes, and lignin (e.g. the most recalcitrant components of lignocellulosic biomass)^3^. It is discovered that the decolorization, detoxification and biomass degradation of white rot fungi can be attributed to a group of important enzymes. Decomposition of lignocellulose material by white rot fungus relies on a variety of lignocellulose degrading enzymes from the extracellular hydrolytic system (i.e. hydrolases such as cellulases and hemicellulases) and ligninolytic system (i.e. oxidative ligninases such as oxidase and peroxidase). The ligninolytic enzyme system is directly involved in pollutant degradation. In order to deeply understand the fungal degrading system for the variety of substrates, study of the whole fungus proteome is crucial to decipher the molecular and cellular degrading mechanism for those xenobiotic compounds.

**Figure 1:**
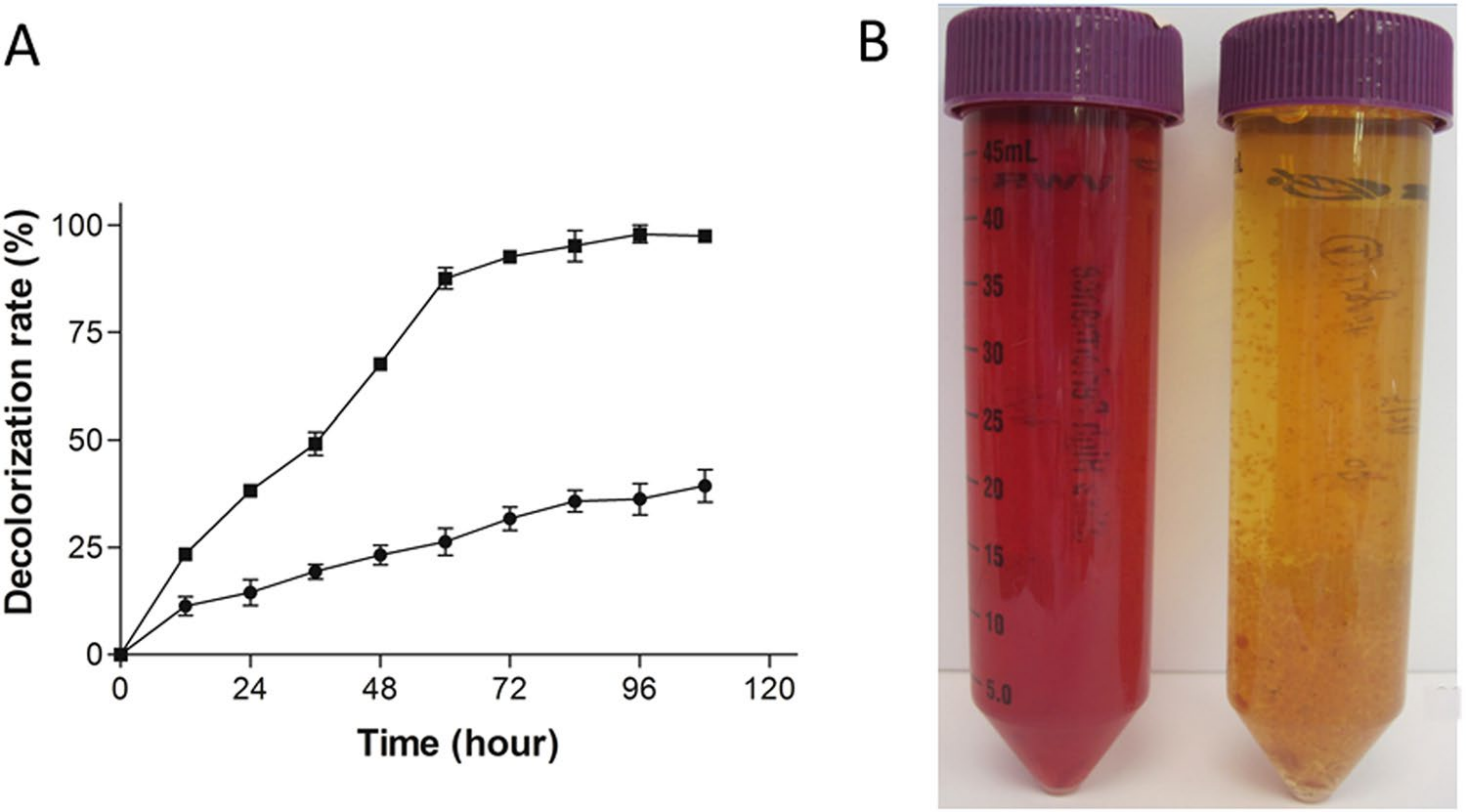
Azo dye DR5B degradation by *I*. *lacteus* CD2. (**A**). Comparison of azo dye DR5B (100 mg/L) decolorization rate by *I*. *lacteus* CD2 with 30 mg/L alkali lignin in medium (solid square) and without alkali lignin in medium (solid circle). (**B**) Photographic comparison of DR5B (100 mg/L) decolorization by *I*. *lacteus* CD2 with 30 mg/L alkali lignin in medium (right) and without alkali lignin in medium (left) after 96 hours.

With regards to environmental pollution, broad application of azo dye in textile, leather, and printing industries poses a major threat to the environment and public health, considering that azo dyes are structurally stable, highly toxic, and potentially carcinogenic^4, 5^. Recent scientific advancement has discovered that white rot fungus can degrade a wide variety of recalcitrant aromatic compounds^2, 3, 6^, which also includes azo dye compounds and lignin. Particularly, our recent discovery that the degradation of azo dye can be promoted in the presence of lignin (Fig. 1), opens up an opportunity to investigate molecular mechanisms of efficient dye degradation by white-rot fungi.

The fungus proteome study has been facilitated by the more public available genome databases and proteomics technology developments^7–14^. Previous ‘omics’ analyses have revealed crucial genetic mechanisms for lignin degradation by white-rot fungi^15, 16^. These studies have led to the discovery of some critical enzymes for lignin degradation, including laccase, cellobiose dehydrogenase, manganese peroxidase (MnP) and other lignin degradation-related enzymes. However, it is still largely unknown how these enzymes work with other proteins to achieve efficient dye degradation, especially under a dye/lignin co-metabolism system. Furthermore, proteins often interact with one another to form a network to carry out biological functions in a dynamic and complex environment. The understanding of such regulatory network will reveal crucial systems level mechanisms to understand fungus degradation capability. Among the various available proteomics platforms^16–19^, the multidimensional protein identification technology (MudPIT) has significant advantages over traditional gel-based proteomics and has been used to quantify differential expression proteins and elucidate relevant regulatory processes^20^. Even though the gel-based proteomics platforms have been used to study white-rot fungus^21, 22^, very few researches have exploited the effective and cutting-edge MudPIT platform to investigate the degradation mechanisms. Further application of the MudPIT platform to study the white-rot fungal degrading system can potentially lead to new scientific discoveries and novel biotechnology applications.

In this study, we employ the MudPIT-based shot-gun proteomics platform to investigate the enhanced degradation of dye by the white-rot fungus *I*. *lacteus* CD2 in the presence of lignin. Global protein expression profile for *I*. *lacteus* CD2 are analyzed for azo dye direct red 5B (DR5B) treatment with and without lignin as a co-substrate. Different conditions are tested to identify key enzymes, pathways, and functional categories involved in lignin and dye degradation. Further time-course analysis revealed the dynamics in the co-regulatory network and coordination between different genes and pathways. Particularly important, we discover that the oxidation and reduction pathway is significantly differentially regulated when mixing textile dye and the synergetic substrate lignin together with the fungus. An *in vitro* validation system simulating the oxidation environment is used to verify the significance of oxidation and reduction pathways. Our results highlight the importance of key enzymes to generate an oxidative environment and redox reaction network for efficient dye degradation.

## Results and Discussion

The goal of the current study is to elucidate the molecular mechanism on how the decolorization of azo dye by the white rot fungus *I*. *lacteus* CD2 is enhanced in the presence of lignin using the proteomics approach. Toward this end, the experiments were designed to compare the azo dye DR5B decolorization efficiency in the presence and absence of lignin in the *I*. *lacteus* CD2 media.

### Characterization of the proteomics platform variation and decolorization of azo dye DR5B by *I*. *lacteus* CD2 with lignin

A comparative proteomics analysis of white-rot fungus *I*. *lacteus* CD2 under dye, lignin and dye/lignin combined treatments is carried out. For DR5B decolorization, the pre-cultured *I*. *lacteus* CD2 is inoculated into modified Kirk medium supplied with 100 mg/L DR5B and cultivated at 30 °C as described in Material and Method section. Synergistic enhancement of azo dye DR5B decolorization by lignin is investigated by comparison of the dye color when incubated in the *I*. *lacteus* CD2 media in the presence and absence of lignin. DR5B is completely decolorized by *I*. *lacteus* CD2 after 120 h in the presence of lignin. Only 49.6% of DR5B is decolorized in the media without lignin for the same incubation period (Fig. 1A). The study also shows that DR5B decolorization rate is significantly improved when lignin is used as a co-substrate (Fig. 1A). The synergistic improvement of dye degradation by lignin can be visualized clearly (Fig. 1B). The incubation of DR5B and lignin alone without fungus reveals that dye decolorization through physical adsorption by lignin is limited (less than 5%, data not shown). The results clearly indicate that the DR5B decolorization by fungus *I*. *lacteus* CD2 could be promoted in the presence of lignin significantly.

During the six days experiment to study the decolorization, a time course proteomics experiment is designed to sample the fungus on day 1, day 3, and day 5. Three biological triplicates are collected for each condition in the proteomics study. The protein numbers identified in each biological triplicate are listed in Supplementary Table 1. The statistical outlier tests (Mendal and Dixon outlier test) were used to identify the outliers in the protein identification numbers. The experiments when using dye as the only substrate on day one has an outlier (identification number of 755 proteins). Generally, the average identified protein numbers by this platform are between 2200–2400 with the relative standard deviation (RSD) value below 20%. In certain proteomics experiments, there are significantly less proteins identified than the other biological replicates, probably due to the complications in the sample preparation process. Typically, a fungal genome denotes around 9000 proteins, and our transcriptomics study suggests that potentially around 5000 proteins can be expected under the experimental conditions. The final determination of protein IDs is the sum of all three biological replicates, which reflects the global protein expression patterns with a better coverage. In the quantitative differential analysis, the proteins that show a changed expression pattern are categorized into two groups, as up-regulated proteins and down-regulated proteins (Supplementary Table 2). The maximum differentially expressed protein number is found on day 3, when 204 proteins are up-regulated, and 124 proteins are down-regulated in the lignin/dye combined treatment compared to the control.

Although previous researches show that the lignin-related compounds could promote aromatic dye degradation when incubating with white-rot fungus, very few studies reveal the systems and molecular mechanisms involved in the decolorization process. The dye-lignin co-metabolism system established in this study offers an ideal model to investigate the synergistic effects and molecular mechanisms for efficient azo dye degradation. Our semi-quantitative proteomics analysis of key enzymes, co-factors, and regulators involved in dye decolorization helps to decipher the regulatory network for enhanced dye degradation by lignin.

### Overview of functional categories for differentially expressed proteins

**Figure 2:**
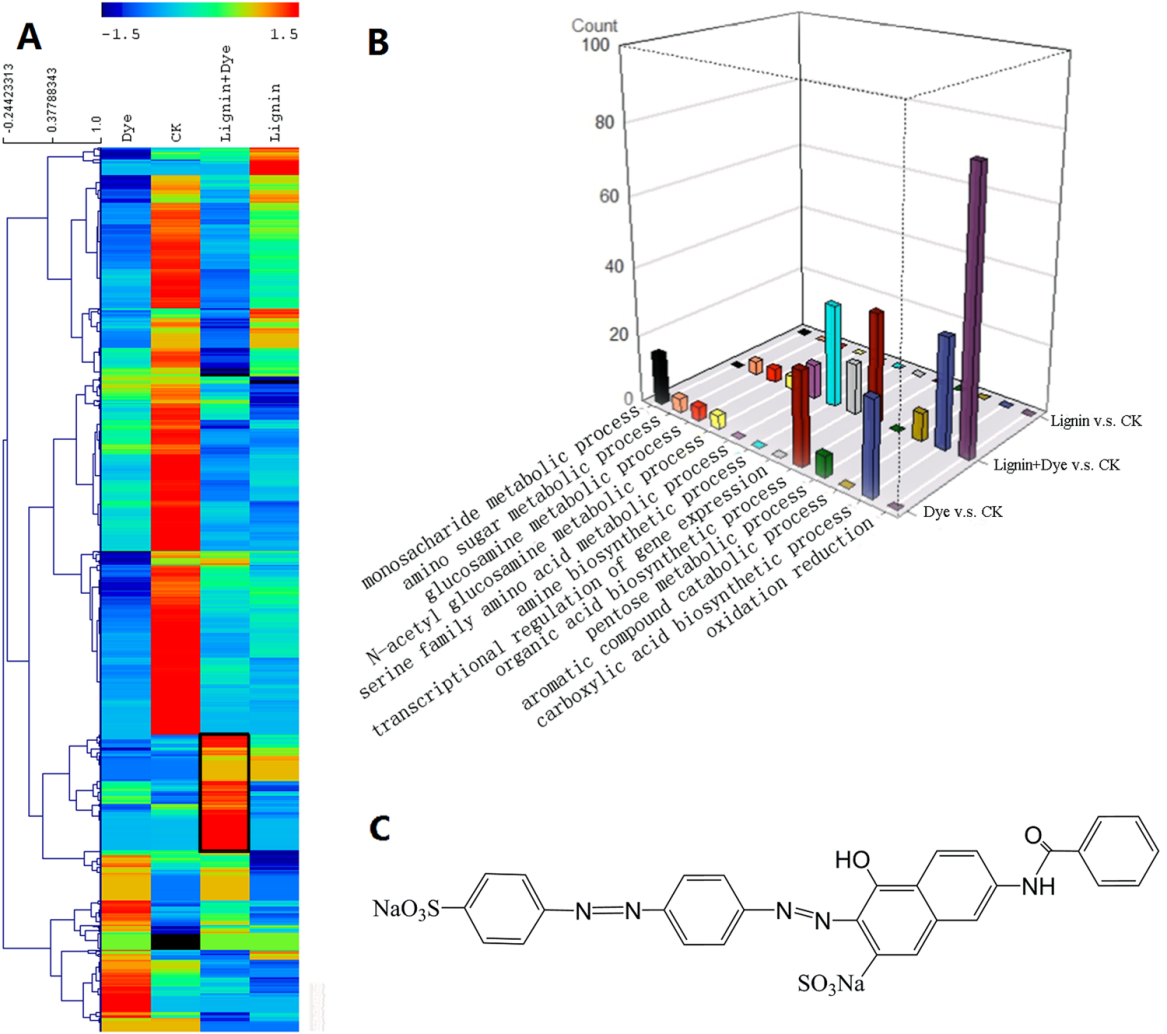
(**A**) Hierarchical cluster overview of differentially expressed proteins among the four different treatment conditions after 72 hours. Totally 896 differentially expressed proteins (*p* value < 0.05 and change fold > 2) are presented in this heat map. Each row represents one protein and each column represented one treatment condition. The enriched functional categories of the black frame highlighted are listed in Table 1. (**B**) Functional category enrichment analysis of the differentially expressed proteins between different conditions. The 896 differentially expressed proteins in Fig. 2A among the compared conditions are used for functional enrichment analysis. The *x* axis indicates the functional categories which are significantly enriched in at least one pair of compared conditions (FDR < 50% and *p* value < 0.05). The *y* axis indicates the compared treatment conditions. The *z* axis indicates the count of the proteins in each functional category. “CK” refers to the condition of *I*. *lacteus* CD2 growing on Kirk medium; “Dye” refers to the condition of *I*. *lacteus* CD2 growing on Kirk medium supplied with 100 mg/L DR5B; “Lignin + Dye” refers to the condition of *I*. *lacteus* CD2 growing on Kirk medium supplied with 100 mg/L DR5B and 30 mg/L lignin; “Lignin” refers to the condition of *I*. *lacteus* CD2 growing on Kirk medium supplied with 30 mg/L lignin. (**C**) Structure of DR5B.

Comparative proteomics analysis is conducted for *I*. *lacteus* CD2 growing on dye, lignin, lignin and dye co-treatment, as well as control Kirk medium. Protein quantitative analysis reveals that the protein expression profile in *I*. *lacteus* CD2 is very dynamic in responses to different treatments, as a total of 896 proteins were differentially expressed under the aforementioned four conditions after 72 h cultivation (Fig. 2A, Supplementary Table 3). The overview of cluster analysis reveals that a large group of proteins are down-regulated in lignin, dye or combined treatments as compared to the control group. Each treatment induces expression of a signature group of proteins (Fig. 2A, Figure S1). Detailed analysis of functional category enrichment is carried out for a group of enzymes induced specifically by the dye/lignin combined treatment (Fig. 2A). The analysis indicates that the combined treatment significantly induces the expression of a group of proteins in oxidation reduction and serine hydrolase activity based on gene ontology (GO) (Table 1).

**Table 1:** The enriched functional categories of the overexpressed subcluster in lignin/dye combined treatment condition.

Category	Term/Pathway	Gene #	Gene %	P-value	FDR
GO:0055114	oxidation reduction	22	23.6	0.0193	23.7
GO:0008236	serine-type peptidase activity	4	4.3	0.0065	7.7
GO:0017171	serine hydrolase activity	4	4.3	0.0065	7.7
GO:0004091	carboxylesterase activity	4	4.3	0.0630	55.0
GO:0016298	lipase activity	3	3.2	0.0756	61.9
SP_PIR_KEYWORDS	oxidoreductase	19	20.4	0.0568	49.9
SP_PIR_KEYWORDS	signal	8	8.6	0.0644	54.5
SP_PIR_KEYWORDS	Secreted	4	4.3	0.0701	57.7
SP_PIR_KEYWORDS	iron	8	8.6	0.0755	60.5
SP_PIR_KEYWORDS	lipid degradation	3	3.2	0.0816	63.5

The cluster analysis outcome is consistent with the comprehensive functional category analysis of all 896 differentially expressed proteins as visualized in Fig. 2B. A total of 12 enriched GO is identified among the four conditions (Fig. 2B). As shown in the 3D plot, the dye treatment and dye/lignin combined treatment leads to the enrichment of several functional categories. For example, monosaccharide metabolic process, organic acid biosynthetic process and carboxylic acid biosynthetic process are more enriched in *I*. *lacteus* CD2 grown on dye. Furthermore, when lignin is added into the dye decolorization system, the protein expression of several dye and/or lignin degradation related functional categories are significantly induced (Fig. 2B, Supplementary Table 4). These categories included oxidation reduction, aromatic compound catabolic process, transcriptional regulation of gene express, amine biosynthetic process and serine family amino acid metabolic process.

These cluster and functional category analyses offer new insights into the molecular mechanisms of efficient DR5B decolorization by white-rot fungus. Considering that lignin significantly promotes the dye decolorization, the functional categories enriched in dye/lignin combined treatment should have contributed to the increased dye decolorization. Among these functional categories, the oxidation reduction category is significantly enriched in dye/lignin combined treatment. It is well known that lignin and dye depolymerization is an oxidoreduction reaction. The enzymes in oxidation reduction functional category could play critical roles in lignin and dye catabolism processes. Many well studied enzymes have been classified into the oxidation reduction category, including MnP, versatile peroxidase (VP), lignin peroxidase, laccase, and other H_2_O_2_ generation oxidoreductase^3, 15, 16, 23^. Besides the lignin and dye depolymerization, the degradation process would release various aromatic monomers. The functional categories of aromatic compound catabolic process, amine biosynthetic process and serine family amino acid metabolic process are all relevant to the aromatic compound catabolism. For example, serine hydrolase is one of the largest enzyme families, where some of the serine hydrolases from different species are shown to be involved in aromatic compound cleavage pathway and to have high catalytic activity on some C-C bond hydrolysis^24, 25^.

Overall, both cluster and functional category analyses confirm the significant synergy between DR5B and lignin on regulating the protein expression to promote dye degradation. Some key functional categories for dye decolorization like oxidoreduction reactions and aromatic compound degradation are only enriched under dye/lignin co-metabolism conditions. The detailed analysis of proteins and networks under dye/lignin combined treatment further revealed molecular mechanisms for efficient dye degradation as well as synergy between DR5B and lignin. The enhancement of dye decolorization facilitated by the oxidation reduction environment is further validated *in vitro*.

### Key oxidation reduction related enzymes during dye decolorization

**Figure 3:**
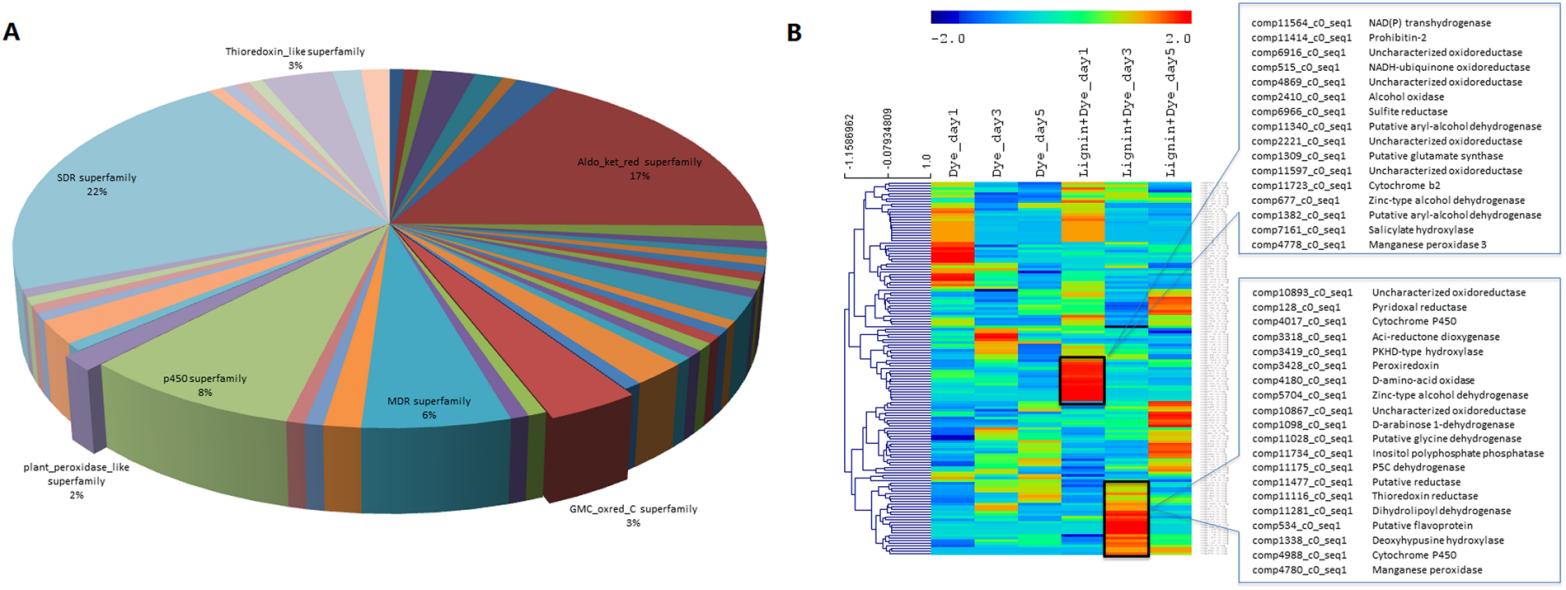
Dynamic of oxidation reduction related proteins during DR5B decolorization by *I*. *lacteus* CD2 in presence of lignin or without lignin. The samples from three time points (1 day, 3days and 5 days) of each treatment are included in the analysis. Totally 143 oxidation reduction related proteins are identified as differentially expressed proteins (*p* value < 0.05 and change fold > 2) among these sample and used for conserved domain analysis (**A**) and hierarchical cluster analysis (**B**). (**A**) shows the percentages of proteins in each superfamily based on conserved domain analysis. Each row of the hierarchical cluster heatmap represents one protein and each column represented one sample. The sample labels of “Dye” and “Lignin + Dye” represent the same conditions described in Fig. 2 legend, and “day1”, “day2” and “day3” represent the samples are collected from the time points of 1 day, 2 day and 3 day respectively.

Among all proteins detected in the proteomics study, 541 proteins were classified into the functional category of oxidation reduction based on GO term, and 143 of them were differentially expressed during dye decolorization by *I*. *lacteus* CD2 under different lignin and dye treatments (Supplementary Table 5). Further domain analysis of these differentially expressed oxidation reduction-related proteins shows redox transformation-related superfamilies account for the majority of differentially regulated proteins. The relevant protein families included short-chain dehydrogenases/reductases (SDR) superfamily, Aldo_ket_red superfamily, p450 superfamily and medium-chain dehydrogenases/reductases (MDR) superfamily (Fig. 3A). During dye/lignin degradation by white-rot fungi, various highly active reaction intermediates will be produced to cause the redox changes^26^. Efficient redox transformation is critical for transforming these reaction intermediates into non-toxic and stable chemicals, which is essential for both efficient dye/lignin degradation and self-protection against radicals.

In addition, Glucose-methanol-choline (GMC) -oxidoreductase superfamily and plant peroxidase-like superfamily are also differentially regulated, and these proteins accounted for a significant percentage of the total differentially expressed oxidation reduction proteins during dye/lignin degradation. GMC-oxidoreductase superfamily is heavily involved in H_2_O_2_ generation in fungus^3, 27, 28^. H_2_O_2_-dependent plant peroxidase-like superfamily is a major group of lignin and dye depolymerization enzymes in white-rot fungi and can utilize H_2_O_2_ generated from GMC-oxidoreductase superfamily^3, 16^. The synergy between these two groups of enzymes could be the main driving force for the efficient lignin and dye degradation.

The expression profile of the aforementioned oxidation reduction-related proteins is further studied in a time-course analysis of different dye degradation stages. The cluster analysis reveals the dynamic expression profile of these proteins (Fig. 3B). Analysis of overexpressed proteins under dye/lignin combined treatment reveals two over-expressed MnPs during dye degradation in the presence of lignin. One of the MnPs (comp4778_c0_seq. 1) is specifically overexpressed on the first day during dye/lignin co-metabolic decolorization, and the other MnP protein (comp4780_c0_seq. 1) is overexpressed after three days under the dye/lignin combined treatment (Fig. 3B). Meanwhile, there are several proteins with potential H_2_O_2_ generation activity expressed with a similar pattern as MnP. For example, alcohol oxidase (comp2410_c0_seq. 1) and several uncharacterized oxidoreductases (comp2221_c0_seq. 1, comp4869_c0_seq. 1, comp11597_c0_seq. 1 and comp6916_c0_seq. 1) are overexpressed and clustered together with MnP (comp4778_c0_seq. 1) on the first day after dye/lignin combined treatment (Fig. 3B). Alcohol oxidase has been widely studied as a main H_2_O_2_ generating source in fungus. Those uncharacterized oxidoreductases in the same cluster might also contribute to the H_2_O_2_ generation or other redox transformation processes to provide oxidative environment for lignin depolymerization. Even though some H_2_O_2_ generation enzymes such as Pyranose 2-oxidase (comp586_c0_seq. 1) are highly expressed under DR5B only treatment conditions, no MnP or similar proteins are overexpressed in the absence of lignin. Therefore, MnP might have played a central role in dye decolorization, and H_2_O_2_ generation enzymes act in a chemical reaction network to efficiently supply H_2_O_2_ for MnP.

### Co-regulatory network analysis revealed essential modules for efficient dye degradation

To further understand the mechanism of dye degradation at the systems level, we conducted co-regulatory network analysis based on proteomics^29, 30^. All of the 4413 proteins from aforementioned eight conditions are used as input for the co-regulatory network analysis. A total of 37 co-expression modules (each module was named by color) are identified with the number of proteins in each module ranging from 11 to 711 (Supplementary Table 6). Five modules (coded as brown, green, light-yellow, midnight-blue, and pink) are enriched with oxidation reduction process based on the GO terms. The brown coded module has the most significant enrichment in oxidation reduction process and other processes related to aromatic compound metabolism, including “aromatic compound catabolic process”, “aromatic amino acid family metabolic process”, “L-phenylalanine catabolic process”, “ubiquinone metabolic process”, and “tyrosine metabolic process” (Supplementary Tables 6 and 7). The 24 proteins that have potential signal peptides are listed in Table 2. These 24 secreted proteins include MnP (comp327_c0_seq. 1), VP (comp596_c0_seq. 1) and choline dehydrogenase (comp4485_c0_seq. 1) (Table 2). As aforementioned, MnP and VP are the major lignin depolymerization enzymes characterized in white-rot fungi. Choline dehydrogenase is widely believed to be involved in extracellular H_2_O_2_ generation in different wood degradation fungi^31^. These potentially secreted enzymes in this functional module might play an essential role in dye decolorization through an efficient redox transferring network, where H_2_O_2_ is generated by choline dehydrogenase to provide the oxidative environment for MnP and VP to degrade azo dyes and lignin. Meanwhile, fourteen proteins related to aromatic compound metabolism are also found in the same module (Table 3). Among these proteins, three of them (comp1651_c0_seq. 1, comp5239_c0_seq. 1 and comp5246_c0_seq. 1) encodes homogentisate 1,2-dioxygenase; another three (comp10990_c0_seq. 1, comp1364_c0_seq. 1 and comp395_c0_seq. 1) encode epoxide hydrolase; one (comp11130_c0_seq. 1) encodes phenylalanine ammonia-lyase; and one (comp328_c0_seq. 1) encodes phenol 2-monooxygenase. All of these enzymes are involved in aromatic compound catabolism. The classification of all these enzymes into one co-regulatory network indicates their coordinative roles in the efficient dye and lignin degradation. Further functional validation is performed based on the co-regulatory network analysis.

**Table 2:** Proteins with potential signal peptides in brown module from the co-expression network analysis.

Protein ID	Protein name	Connections
comp1049_c0_seq. 1	UPF0357 protein C1687.07	1010
comp11108_c0_seq. 1	Peroxidase 2	1333
comp11546_c0_seq. 1	Probable glycosidase C21B10.07	1010
comp11577_c0_seq. 1	Dolichyl-diphosphooligosaccharide–protein glycosyltransferase subunit 1	1305
comp11696_c0_seq. 1	Probable ATP-dependent permease	1242
comp1296_c0_seq. 1	Chitinase 1	1010
comp1376_c0_seq. 1	Protein disulfide-isomerase	1841
comp1434_c0_seq. 1	Endoglucanase EG-II	1010
comp1859_c0_seq. 1	NADH dehydrogenase [ubiquinone] complex I, assembly factor 7	1010
comp2041_c0_seq. 1	Thioredoxin domain-containing protein C13F5.05, mitochondrial	1270
comp2452_c0_seq. 1	Histidine-rich membrane protein KE4 homolog 1	1010
comp2645_c0_seq. 1	Lipase 1	1496
comp3035_c0_seq. 1	Extracellular metalloproteinase MEP	1431
comp327_c0_seq. 1	Manganese peroxidase 3	1010
comp358_c0_seq. 1	Ribonuclease T2	1010
comp3938_c0_seq. 1	Glucan endo-1,3-alpha-glucosidase agn1	1010
comp4459_c0_seq. 1	Oxalate decarboxylase OxdC	1570
comp4485_c0_seq. 1	Choline dehydrogenase	2032
comp5320_c0_seq. 1	Phosphatidylglycerol/phosphatidylinositol transfer protein	1381
comp596_c0_seq. 1	Versatile peroxidase VPL1	1304
comp6414_c0_seq. 1	Putative agmatinase 1	1010
comp6525_c0_seq. 1	Oxalate decarboxylase OxdC	1010
comp7357_c0_seq. 1	Probable endo-1,3(4)-beta-glucanase An02g00850	1010
comp9647_c0_seq. 1	Probable glycosidase C21B10.07	1010

**Table 3:** Proteins involved in aromatic compounds degradation in brown module from the co-expression network analysis.

Protein ID	Protein name	Connections
comp10990_c0_seq. 1	Putative epoxide hydrolase	1913
comp11130_c0_seq. 1	Phenylalanine ammonia-lyase	996
comp1364_c0_seq. 1	Putative epoxide hydrolase	1459
comp1531_c0_seq. 1	Probable prephenate dehydrogenase [NADP( + )]	1768
comp1651_c0_seq. 1	Homogentisate 1,2-dioxygenase	2003
comp223_c0_seq. 1	Aryl-alcohol dehydrogenase [NADP( + )]	1475
comp328_c0_seq. 1	Phenol 2-monooxygenase	990
comp3325_c0_seq. 1	Probable anthranilate synthase component 1	1897
comp395_c0_seq. 1	Putative epoxide hydrolase	1591
comp4631_c0_seq. 1	Tryptophan synthase	1135
comp5239_c0_seq. 1	Homogentisate 1,2-dioxygenase	1571
comp5246_c0_seq. 1	Homogentisate 1,2-dioxygenase	1584
comp596_c0_seq. 1	Versatile peroxidase VPL1	1304
comp7161_c0_seq. 1	Salicylate hydroxylase	1912

### Functional validation of proteomics-derived hypothesis

**Figure 4:**
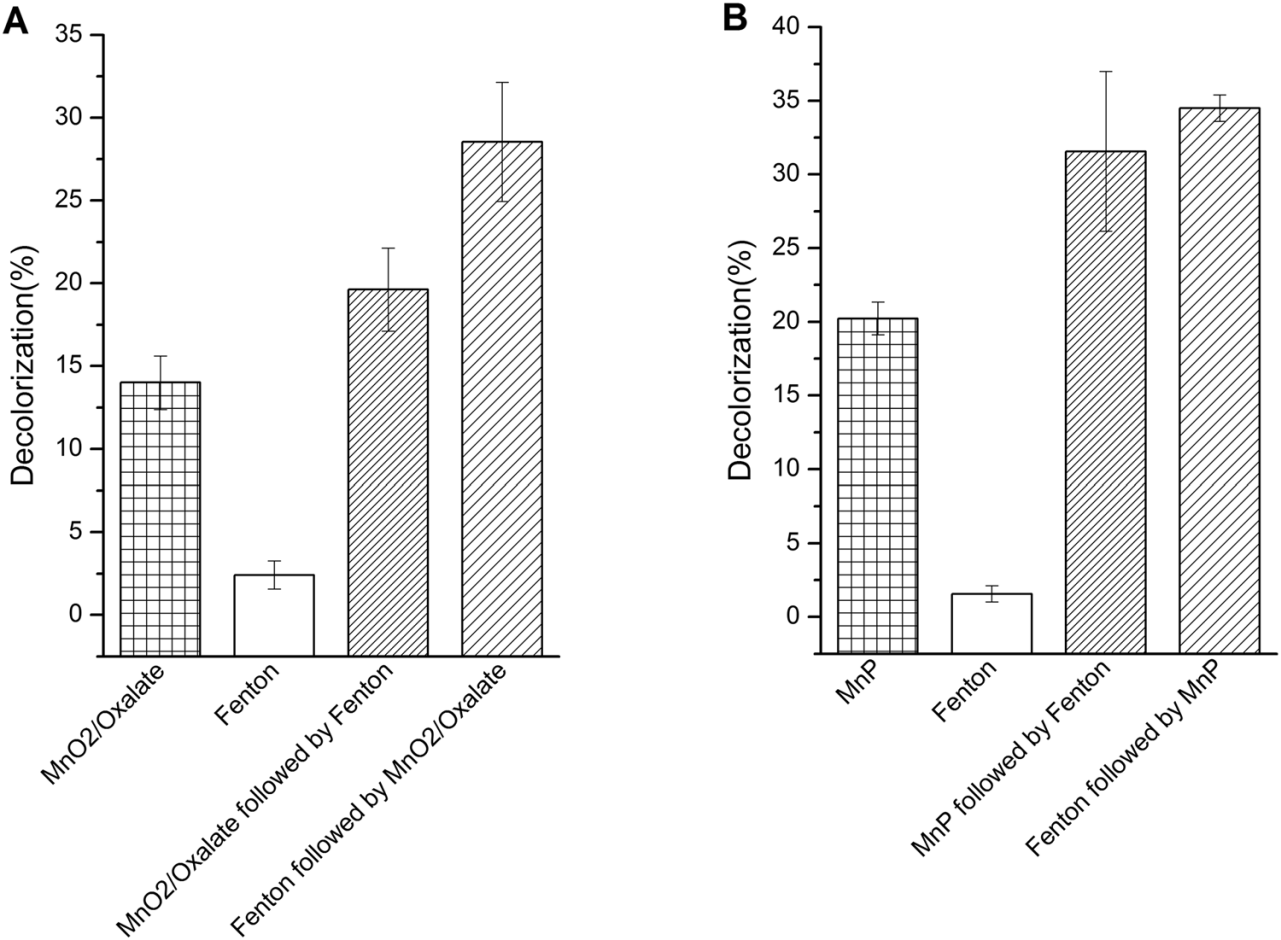
*In vitro* DR5B dye decolorization. (**A**) DR5B dye decolorization by Fenton reaction and MnO_2_/oxalate catalysis. (**B**) DR5B dye decolorization by Fenton reaction and purified MnP. The experiment results are the average of three replicates.

*In vitro* dye decolorization with Fenton reaction and MnO_2_/oxalate catalysis as well as MnP cloned from *I*. *lacteus* CD2 are carried out in order to validate the hypothesis derived from proteomics study. In this study, the MnO_2_/oxalate catalysis is used to mimic the MnP activity. At low pH value conditions, oxalate can reduce MnO_2_ into soluble Mn^3+^ chelate which could act as Mn oxidant to catalyze lignin and pollutant degradation as the same as MnP [52]. To mimic the fungal Fenton reaction condition *in vivo*, the ferrous concentration in Fenton reaction is controlled at a low concentration level, which is at the micromole level (8.75 uM). As is shown in Fig. 4A, the decolorization of DR5B dye with Mn oxidant is enhanced significantly by the following Fenton reaction, and the decolorization ratio of dye by the synergetic reaction (Fenton chemistry followed with Mn oxidation) is doubled compared to the sole Mn oxidation. The synergy is more significant than an additive effect, as the Mn oxidation alone only decolorizes about 13% of the original dye and the Fenton chemistry alone only decolorizes 2% of the original dye, and the synergy in Fenton chemistry followed with Mn oxidation decolorizes about 27% of the original dye. The very similar result is obtained with the *In vitro* dye decolorization experiment by MnP and Fenton reaction (Fig. 4B). This phenomenon indicates that Fenton reaction with trace ferrous during the fungal degradation could synergize the oxidative degradation with manganese oxidant induced by MnP. This result well confirms the proteomics-derived hypothesis that the synergy of Fenton reaction and manganese peroxidase might play an important role in DR5B dye degradation.

### Shotgun proteomics could be explored for guiding the design of effective bioremediation process

The shotgun MudPIT proteomics study identified around 2000 proteins covering about one third of the expected white-rot fungal proteome, and presented as one of the most powerful platforms to characterize the white rot fungal proteome system. As shown the current study, the azo dye DR5B degradation by the white-rot fungus could be significantly promoted in the presence of lignin due to the induction of an efficient enzymatic network for polyaromatic compound depolymerization and further aromatic catabolism. From the proteomics study, the key functional categories were revealed for polyaromatic compound degradation (i.e. the two categories of oxidative reduction and aromatic compound degradation were only enriched during the dye/lignin co-metabolism). Key enzymes in these functional categories like MnP are also up-regulated by the dye/lignin combined treatment. Further time-course proteomics analysis of co-metabolism conditions enabled the mapping of co-regulatory network highlighting the coordination of two key steps of polyaromatic compound degradation. In general, there are two steps for azo dye or lignin degradation: depolymerization of heterogeneous polymers into monomers and catabolism of aromatic monomers. From the co-regulatory network analysis, our proteomics study and *in vitro* validation suggest that in the first step, MnP and VP could synergize with H_2_O_2_ generation enzymes (i.e. alcohol oxidase and choline dehydrogenase) and other oxidation enzymes to establish an extracellular redox transferring network to efficiently oxidize dye or lignin for depolymerization. In the second step, several key aromatic compound catabolism enzymes are identified to play pivotal roles in complete dye or lignin degradation. Particularly in the current study, homogentisate 1,2-dioxygenase is discovered to be significantly overexpressed and coordinates with the depolymerization enzymes in the co-regulatory network. Overall, the present proteomics analysis platform has demonstrated the use of shotgun proteomics platform can further expand our knowledge of the white-rot fungus proteome and underline the mechanisms for efficient lignin and aromatic compound degradation by white-rot fungus. The scientific principle and key components discovered in this study can be used to guide the development of effective bioremediation of aromatic dye as well as efficient lignin and lignocellulosic biomass conversion.

## Materials and Methods

### Microorganism and growth condition

The white rot fungus *Iprex lacteus* CD2 has been characterized as an efficient lignin degrader in previous studies^32, 33^. The fungus was pre-cultured on 100 mL modified Kirk medium (containing 1% glucose as carbon source)^34^ in 250 mL Erlenmeyer flasks at 30 °C with shaking speed of 150 rpm for 7 days before dye decolorization.

### Dye decolorization

For DR5B decolorization, 10 mL of the pre-cultured *I*. *lacteus* CD2 was inoculated into 100 mL modified Kirk medium supplied with 100 mg/L DR5B (purchased from Colorfran S.A., Mexico) and cultivated at 30 °C with the shaking speed at 150 rpm for 5 days. To understand the effect of alkali lignin (purchased from Sigma-Aldrich, USA) on DR5B decolorization by *I*. *lacteus* CD2, 30 mg/L of lignin was added to the aforementioned dye decolorization medium. Meanwhile, additional two experiments were set up as controls: 1) *I*. *lacteus* CD2 grown on modified Kirk medium; 2) *I*. *lacteus* CD2 grown on modified Kirk medium supplied with 30 mg/L of lignin. The decolorization rate of DR5B was measured by UV/vis spectrophotometer at the wavelength 510 nm.

### Total protein extraction

The mycelium of the white-rot fungus from aforementioned culture was collected by centrifuging at 5000 × g, washed twice with double-distilled H_2_O and briefly dried with tissue paper. 100 mg of the harvested mycelium sample was then grounded in liquid nitrogen to fine powder and boiled for 10 min in 1 mL Alkali-SDS buffer (5% SDS; 50 mM Tris-HCl, pH 8.5; 0.15 M NaCl; 0.1 mM EDTA; 1 mM MgCl_2_; 50 mM Dithiothretiol)^35^. The clear supernatant of the boiled sample after centrifuging at 3000 × g for 10 min was collected and transferred to a fresh tube. To each tube, chilled 100% tricholoroacetic acid (TCA) was added to a final concentration of 20%. The solution was mixed well and incubated at −20 °C for 2 hours. Samples were centrifuged at 16,000 × g for 30 min at 4 °C to remove the supernatant. The pellet was harvested and washed twice with 1 mL chilled acetone following by centrifuging at 16,000 × g for 30 min at 4 °C. The protein pellet was air-dried and then dissolved in solution buffer containing 7 M urea, 2 M thiourea, 40 mM triszma base, and 1% 3-(4-Heptyl)phenyl-3-hydroxypropyl dimethylammoniopropanesulfonate (C7BzO). The extracted protein was stored at −80 °C prior to LC-MS/MS analysis.

### MudPIT based shot-gun proteomics

MudPIT-based shot-gun proteomics was carried out to analyze each the extracted protein. Approximately 100 μg of protein was digested by Trypsin Gold, Mass Spectrometry Grade (Promega, WI, USA) with 1:40 w/w at 37 °C for 24 h. The digested peptides were desalted using a Sep-Pak plus C18 column (Waters Limited, ON, Canada) and then loaded onto a biphasic (strong cation exchange/reversed phase) capillary column using a pressure tank. The 2D back column was composed of 5 cm of C18 reverse phase resin (C18-AQ, The Nest Group, Inc, Southborough, MA, USA) and 3 cm of strong cation exchange (SCX) resin PolySULFOETHYL A, (The Nest Group, Inc, Southborough, MA, USA). The back column was then connected to a 15-cm-long 100 um-ID C18 column (packed in house with the same C18 reverse phase in the back column) and sprayed through a SilicaTip (New objective, Inc, Woburn, MA). The two-dimensional liquid chromatography separation and tandem mass spectrometry conditions followed the protocols previously described by Washburn *et al*.^36^. Basically, Before SCX separation, a 1 h RP gradient from 100% Solvent A (95% H2O, 5% ACN, and 0.1% formic acid) to 100% Solvent B (30% H2O, 70% ACN, and 0.1% formic acid) was configured to move peptides from C18 resin to SCX resin in the back column. The SCX LC separation was performed with eleven salt pulses containing increasing concentrations of ammonium acetate. Each salt pulse was followed by a 2 h reverse phase gradient from 100% Solvent A to 60% Solvent B. The LC eluent was directly nanosprayed into a linear ion trap mass spectrometer, Finnigan LTQ (Thermo Fisher Scientific, San Jose, CA). The mass spectrometer was set to the data-dependent data acquisition mode, and full mass spectra were recorded on the peptides over a 300–1700 m/z range, followed by five tandem mass (MS/MS) events for the most abundant ions from the first MS analysis. The Xcalibur data system (Thermo Fisher Scientific, San Jose, CA) was used to control the LC-LTQ system and collect the data. The isolated total protein from the mycelium of *I*. *lacteus* CD2 was analyzed by MudPIT-based shot-gun proteomics system as described in previous publications^19, 37^. In summary, the extracted protein was digested by Mass Spectometry Grade Trypsin (Promega. USA) and then loaded onto an in-house packed two phase column constituted of strong cation exchange and C18 reverse phase to implement the two dimensional chromatography separation. The total peptides in analytical columns was acquired and analyzed by LTQ mass spectrometry (Thermo Finnegan) for MS/MS analysis.

### Proteomics data analysis

Tandem mass spectra were extracted from the raw files and converted into the MS2 file. The MS2 file was searched against the *I*. *lacteus* CD2 protein database established based on the previous transcriptomic analysis of *I*. *lacteus* CD2^38^. A ProLuCID algorithm was used to search for data using the Texas A&M Supercomputing Facility^39^. The validity of peptide/spectrum matches was assessed in DTASelect2.0 using a 0.05 false discovery cutoff, with a cross-correlation score (XCorr) that’s larger than 1, and normalized difference in cross-correlation scores (DeltaCN) larger than 0.08. Proteins with more than two peptides were identified as detected and were recorded^19, 40^. The differential expression proteins were identified by PatternLab with TFold module between two samples^41, 42^. The blue-dot proteins which have p-values of  < 0.05 and an absolute fold change >2.5 were classified as differentially expressed proteins. In the differential protein expression analysis, counting of differently expressed proteins includes all the proteins that have been identified as differentially expressed proteins in either one pair of analysis.

### Functional annotation and classification

Gene Ontology (GO) annotations for the protein were conducted by using InterproScan (http://www.ebi.ac.uk/Tools/pfa/iprscan/). The Database for Annotation, Visualization and Integrated Discovery (DAVID) was used to analyze the functional enrichment with false-detection rate (FDR) <50% and the *p-*value <0.05^43^. Potential signal peptides of the proteins were predicted by SignalP4.0^44^.

### Protein co-expression network analysis

All the detected proteins from the aforementioned eight different conditions were used for protein co-regulatory network analysis using Weighted Gene Co-expression Network Analysis (WGCNA) package^45^. The key parameter, β, for weighted network construction was optimized to maintain both the scale-free topology and sufficient node connectivity as recommended by the manual. Networks were visualized using Cytoscape, and Edge-wighted Spring Embedded layout was applied^46^.

### DR5B dye decolorization by Fenton reagent and MnO_2_/oxalate

The concentration of DR5B dye was 0.05 mg/mL in all *in vitro* dye decolorization reactions. Fenton reaction was carried out in a total volume of 16 mL reaction mixture containing NaFeEDTA (8.75 μm) and H_2_O_2_ (2.5 mM). The MnO_2_/oxalate reaction was set up in a total volume of 16 mL oxalate buffer (100 mM, pH 2.5) containing MnO_2_ (50 mM). The MnP was purified from *I*. *lacteus* CD2 with the method as we reported in our previous publication^47^. For DR5B dye decolorization by MnP, the reaction was set in sodium malonate (pH 3.0) containing 50 U/L purified MnP, 1 mM MnSO_4_ and 2.5 mM H_2_O_2_.

Four experiment conditions were designed to study the synergy between Fenton chemistry and oxidation for dye decoloring: DR5B dye was subjected to the sole Fenton reaction, the sole MnO_2_/oxalate or MnP reaction, the Fenton reaction followed by MnO_2_/oxalate reaction or MnP, and MnO_2_/oxalate or MnP reaction followed by Fenton reaction, respectively. All reactions were incubated at 25 °C for 48 h.

## Electronic supplementary material

Supplementary materials

## References

[1] Frazzetto G (2003). White biotechnology. EMBO Rep..

[2] Wesenberg D, Kyriakides I, Agathos SN (2003). White-rot fungi and their enzymes for the treatment of industrial dye effluents. Biotechnol. Adv..

[3] Xie S, Syrenne R, Sun S, Yuan JS (2014). Exploration of Natural Biomass Utilization Systems (NBUS) for advanced biofuel-from systems biology to synthetic design. Curr. Opin. Biotechnol..

[4] Golka K, Kopps S, Myslak ZW (2004). Carcinogenicity of azo colorants: influence of solubility and bioavailability. Toxicol. Lett..

[5] Gonzales CA, Riboli E, Lopez-Abente G (1988). Bladder cancer among workers in the textile industry: results of a spanish case-control study. Am. J. Ind. Med..

[6] Saroj S, Kumar K, Pareek N, Prasad R, Singh R (2014). Biodegradation of azo dyes Acid Red 183, Direct Blue 15 and Direct Red 75 by the isolate *Penicillium oxalicum* SAR-3. Chemosphere.

[7] Bianco L, Perrotta G (2015). Methodologies and perspectives of proteomics applied to filamentous fungi: from sample preparation to secretome analysis. Int. J. Mol. Sci..

[8] Kim Y, Nandakumar MP, Marten MR (2007). Proteomics of filamentous fungi. Trends Biotechnol..

[9] Bhadauria V (2007). Advances in fungal proteomics. Microbiol. Res..

[10] Tian C (2009). Systems analysis of plant cell wall degradation by the model filamentous fungus *Neurospora crassa*. Proc. Natl. Acad. Sci. USA..

[11] Doyle S (2011). Fungal proteomics: from identification to function. FEMS Microbiol. Lett..

[12] Kroll K, Pahtz V, Kniemeyer O (2014). Elucidating the fungal stress response by proteomics. J. Proteomics.

[13] Rodrigues ML, Nakayasu ES, Almeida IC, Nimrichter L (2014). The impact of proteomics on the understanding of functions and biogenesis of fungal extracellular vesicles. J. Proteomics.

[14] Gonzalez-Fernandez R (2014). Proteomic analysis of mycelium and secretome of different Botrytis cinerea wild-type strains. J. Proteomics.

[15] Martinez D (2004). Genome sequence of the lignocellulose degrading fungus *Phanerochaete chrysosporium* strain RP78. Nat. Biotech..

[16] Fernandez-Fueyo E (2012). Comparative genomics of *Ceriporiopsis subvermispora* and *Phanerochaete chrysosporium* provide insight into selective ligninolysis. Proc. Natl. Acad. Sci. USA..

[17] Gstaiger M, Aebersold R (2009). Applying mass spectrometry-based proteomics to genetics, genomics and network biology. Nat. Rev. Genet..

[18] Altelaar AFM, Munoz J, Heck AJR (2013). Next-generation proteomics: towards an integrative view of proteome dynamics. Nat. Rev. Genet..

[19] Zhang, Y. *et al*. Application of an improved proteomics method for abundant protein cleanup: molecular and genomic mechanisms study in plant defense. *Mol*. *Cell*. *Proteomics* (2013). 10.1074/mcp.M112.025213 PMC3820953 23943779

[20] Yates JR, Ruse CI, Nakorchevsky A (2009). Proteomics by mass spectrometry: approaches, advances, and applications. Annu. Rev. Biomed. Eng..

[21] Matsuzaki F, Shimizu M, Wariishi H (2008). Proteomic and metabolomic analyses of the white-rot fungus *Phanerochaete chrysosporium* exposed to exogenous benzoic acid. J. Proteome Res..

[22] Salvachua D (2013). Differential proteomic analysis of the secretome of *Irpex lacteus* and other white-rot fungi during wheat straw pretreatment. Biotechnol. Biofuels.

[23] Xie S (2015). Simultaneous conversion of all cell wall components by an oleaginous fungus without chemi-physical pretreatment. Green Chem..

[24] Puente XS, López-Otn C (1995). Cloning and expression analysis of a novel human serine hydrolase with sequence similarity to prokaryotic enzymes involved in the degradation of aromatic compounds. J.Biol. Chem..

[25] Hernáez MJ (2000). Identification of a serine hydrolase which vleaves the alicyclic ring of tetralin. J. Bacteriol..

[26] Gómez-Toribio V, García-Martín AB, Martínez MJ, Martínez ÁT, Guillén F (2009). Induction of extracellular hydroxyl radical production by white-rot fungi through quinone redox cycling. Appl. Environ. Microbiol..

[27] Martinez D (2009). Genome, transcriptome, and secretome analysis of wood decay fungus Postia placenta supports unique mechanisms of lignocellulose conversion. Proc. Natl. Acad. Sci. USA.

[28] Vanden Wymelenberg A (2010). Comparative transcriptome and secretome analysis of wood decay fungi *Postia placenta* and *Phanerochaete chrysosporium*. Appl. Environ. Microbiol..

[29] Yuan JS, Galbraith DW, Dai SY, Griffin P, Stewart CN (2008). Plant systems biology comes of age. Trends Plant Sci..

[30] Zhang Y, Gao P, Yuan JS (2010). Plant protein-protein interaction network and interactome. Curr. Genomics.

[31] Yakovlev IA (2013). Genes associated with lignin degradation in the polyphagous white-rot pathogen *Heterobasidion* irregulare show substrate-specific regulation. Fungal Genet. Biol..

[32] Du W (2011). The promoting effect of byproducts from *Irpex lacteus* on subsequent enzymatic hydrolysis of bio-pretreated cornstalks. Biotechnol. Biofuels.

[33] Yu H (2010). Fungal treatment of cornstalks enhances the delignification and xylan loss during mild alkaline pretreatment and enzymatic digestibility of glucan. Bioresour. Technol..

[34] Kirk TK, Schultz E, Connors WJ, Lorenz LF, Zeikus JG (1978). Influence of culture parameters on lignin metabolism by *Phanerochaete chrysosporium*. Arch. Microbiol..

[35] Chourey K (2010). Direct cellular lysis/protein extraction protocol for soil metaproteomics. J. Proteome Res..

[36] Washburn MP, Wolters D, Yates JR (2001). Large-scale analysis of the yeast proteome by multidimensional protein identification technology. Nat. Biotechnol..

[37] Zhang Y, Liu S, Dai S, Yuan J (2012). Integration of shot-gun proteomics and bioinformatics analysis to explore plant hormone responses. BMC Bioinformatics.

[38] Sun S (2016). Genomic and molecular mechanisms for efficient biodegradation of aromatic dye. J. Hazard. Mater..

[39] Xu T (2015). ProLuCID: An improved SEQUEST-like algorithm with enhanced sensitivity and specificity. J. Proteomics..

[40] Xu, T. *et al*. In *Molecular & Cellular Proteomics*. S174-S174 (AMER SOC BIOCHEMISTRY MOLECULAR BIOLOGY INC 9650 ROCKVILLE PIKE, BETHESDA, MD 20814-3996 USA).

[41] Carvalho, P. C., Yates, J. R. & Barbosa, V. C. Analyzing shotgun proteomic data with PatternLab for proteomics. *Current protocols in bioinformatics*/*editoral board*, *Andreas D*. *Baxevanis… [et al*.*]* CHAPTER, Unit-13.1315, doi:10.1002/0471250953.bi1313s30 (2010). 10.1002/0471250953.bi1313s30 PMC2933420 20521246

[42] Carvalho PC (2016). Integrated analysis of shotgun proteomic data with PatternLab for proteomics 4.0. Nat. Protocols.

[43] Huang DW, Sherman BT, Lempicki RA (2008). Systematic and integrative analysis of large gene lists using DAVID bioinformatics resources. Nat. Protocols.

[44] Petersen TN, Brunak S, von Heijne G, Nielsen H (2014). SignalP 4.0: discriminating signal peptides from transmembrane regions. Nat. Meth..

[45] Langfelder P, Horvath S (2008). WGCNA: an R package for weighted correlation network analysis. BMC Bioinformatics.

[46] Smoot ME, Ono K, Ruscheinski J, Wang P-L, Ideker T (2011). Cytoscape 2.8: new features for data integration and network visualization. Bioinformatics.

[47] Qin X, Zhang J, Zhang X, Yang Y (2014). Induction, purification and characterization of a novel manganese peroxidase from *Irpex lacteus* CD2 and its application in the decolorization of different types of dye. PLoS One.

