# Supplementary material for: Enhancement of Environmental Hazard Degradation in the Presence of Lignin: a Proteomics Study
Source: Sci Rep. 2017 Sep 12;7:11356. doi: 10.1038/s41598-017-10132-4 (PMC5595786; doi:10.1038/s41598-017-10132-4)
Supplement: Supplementary file 1 — Supplementary materials [file 41598_2017_10132_MOESM1_ESM.pdf]

Enhancement of Environmental Hazard Degradation in the Presence of Lignin: a Proteomics Study

Su Sun, Shangxian Xie, Yanbing Cheng, Hongbo Yu, Honglu Zhao, Muzi Li,

Xiaotong Li, Xiaoyu Zhang, Joshua S. Yuan, and Susie Y. Dai

Supplementary materials

Supplementary Table 1. The protein numbers identified in each biological triplicate.

Supplementary Table 2. Differentially expressed protein number in each comparison.

Supplementary Table 3. Differentially expressed proteins of *I. lacteus* CD2 among the four treatment conditions after 72 hours.

Supplementary Table 4. The functional enrichment of differentially expressed proteins between dye/lignin combined treatment and dye or lignin as the single substrate.

Supplementary Table 5. Differentially expressed oxidation reduction related proteins during different stages of DR5B decolorization by *I. lacteus* CD2.

Supplementary Table 6. Functional enrichment analysis of each module from total protein co-expression network.

Supplementary Table 7. Protein information in brown module.

Figure S1. Hierarchical cluster overview of the total 896 differentially expressed proteins among the different time course and conditions.

Supplementary Table 1. The protein numbers identified in each biological triplicate.

Sample	Identified Protein Counts	Dixon test* Z-score	Mandel's statistics* h(i)	Identified Peptides Counts
CK-3day-Sample 1	2480	0.968		18817
CK-3day-Sample 2	1729	-0.778	0.788	10093
CK-3day-Sample 3	2519	1.059		20027
Dye-1day-Sample 1	755	**-3.042**		3079
Dye-1day-Sample 2	1728	-0.780	**-1.938**	9479
Dye-1day-Sample 3	2387	0.752		17775
Dye-3day-Sample 1	2222	0.368		16822
Dye-3day-Sample 2	1598	-1.082	-0.167	9112
Dye-3day-Sample 3	2257	0.450		17571
Dye-5day-Sample 1	1451	-1.424		7980
Dye-5day-Sample 2	2091	0.064	-0.46	15176
Dye-5day-Sample 3	2335	0.631		16681
Lignin+Dye-1day-Sample 1	2060	-0.008		13454
Lignin+Dye-1day-Sample 2	2215	0.352	-0.116	16171
Lignin+Dye-1day-Sample 3	1837	-0.527		11786
Lignin+Dye-3day-Sample 1	2386	0.750		18596
Lignin+Dye-3day-Sample 2	2376	0.726	1.277	19546
Lignin+Dye-3day-Sample 3	2299	0.547		17481
Lignin+Dye-5day-Sample 1	2319	0.594		17889
Lignin+Dye-5day-Sample 2	2143	0.185	0.831	13488
Lignin+Dye-5day-Sample 3	2295	0.538		15898
Lignin-3day-Sample 1	1469	-1.382		8012
Lignin-3day-Sample 2	2599	1.245	-0.215	23495
Lignin-3day-Sample 3	1976	-0.204		12784

* The Dixon test was carried out among all the samples and the Mandel's statistics was carried out among biological triplicates. The numbers showed in bold were the outlier.

Supplementary Table 2. Differentially expressed protein number in each comparison.

	Up-regulated proteins				Down -regulated proteins			
	CK *vs.* Lignin	CK *vs.* Dye	CK *vs.* Lignin+Dye	Dye *vs.* Lignin +Dye	CK *vs.* Liginin	CK *vs.* Dye	CK *vs.* Lignin+Dye	Dye *vs.* Lignin +Dye
1 day	--	--	--	13	--	--	--	26
3 day	74	155	204	88	18	78	124	111
5 day	--	--	--	21	--	--	--	81

The protein expression in each sample was compared with each other and the significantly up-regulated and down-regulated protein numbers were recorded.

Supplementary Table 3. Differentially expressed proteins of *I. lacteus* CD2 among the four treatment conditions after 72 hours.

Assembly_ID	Annotated Uniprot ID	Normanized expression abundance							
		CK+Dye _day1	CK+Dye _day3	CK+Dye _day5	CK	Lignin+Dye _day1	Lignin+Dye _day3	Lignin+Dye _day5	Lignin
comp1000_c0_seq1	P22194.2	0.061102	0.058169	0.042012	0.066073	0.028476	0.01551	0.082171	0.057605
comp101_c0_seq1	D4D675.1	0	0.047277	0.018682	0.026764	0.012401	0.027088	0.02624	0.0148
comp1016_c0_seq1	Q5F3J5.1	0.014016	0.022722	0.025554	0.080638	0.032199	0.033565	0.054011	0.052883
comp10168_c0_seq1	O14346.2	0.023318	0.012777	0.014048	0.052924	0.010577	0.002814	0.013485	0.021906
comp10203_c0_seq1	O14123.1	0	0.005822	0.005458	0.025256	0.018093	0.007034	0.00901	0.013117
comp10213_c0_seq1	Q6TGC6.1	0.020819	0.007763	0.006823	0.050941	0.034067	0.011179	0.008785	0.033361
comp10221_c0_seq1	O42643.1	0.004999	0.051079	0.025775	0.085463	0.037406	0.036314	0.013373	0.044204
comp1026_c0_seq1	P37959.2	0.012909	0.007117	0.027507	0.050083	0.015784	0.019878	0.026775	0.05907
comp1030_c0_seq1	P33723.1	0.021105	0.144098	0.099513	0.066677	0.015607	0.13479	0.085903	0.079794
comp1031_c0_seq1	Q9HE05.1	0.075872	0.050648	0.038322	0.151707	0.104807	0.039784	0.057167	0.038389
comp1033_c0_seq1	Q9UKM7.2	0.004999	0.009219	0.005861	0.041675	0.019652	0.013992	0.009093	0.017534
comp10335_c0_seq1	P70627.1	0.057456	0.047036	0.107333	0.134741	0.050792	0.139455	0.127711	0.086681
comp10355_c0_seq1	O14255.1	0.09919	0.08647	0.058931	0.185325	0.109003	0.088351	0.105879	0.086047
comp1036_c0_seq1	Q11119.2	0.132111	0.109166	0.063454	0.206835	0.143392	0.120364	0.081023	0.12179
comp10390_c0_seq1	P0C1B4.1	0	0.003935	0	0.012153	0.003471	0	0	0
comp10397_c0_seq1	Q0CEF3.1	0.027335	0.075742	0.099234	0.205118	0.033581	0.084382	0.107131	0.074264
comp1045_c0_seq1	Q4P4X6.2	0.004877	0.08378	0.06758	0.036596	0.008678	0.117848	0.077617	0.028966
comp1059_c0_seq1	Q9P720.1	0.051064	0.072887	0.048091	0.189546	0.094154	0.082976	0.077675	0.047943
comp10592_c0_seq1	Q5BCI8.1	0	0.018841	0	0.031726	0	0.064271	0	0.032333
comp106_c0_seq1	P0CS63.1	0	0.007763	0.00241	0.009741	0	0	0.002928	0
comp10610_c0_seq1	Q2UN00.1	0	0.035688	0.007709	0.039054	0	0.081716	0.006062	0.031655
comp10633_c0_seq1	Q03529.1	0.003607	0	0	0.020815	0.005207	0.007163	0.004393	0.006733
comp10641_c0_seq1	Q9P7P0.1	0.019836	0.020863	0.011319	0.059128	0.025976	0.019638	0.024417	0.027633
comp1067_c0_seq1	Q09779.3	0.014712	0.003558	0.012281	0.050512	0.003648	0.009356	0.021264	0.018801
comp107_c0_seq1	P87058.1	0.072429	0.045633	0.023897	0.132438	0.083039	0.044005	0.024125	0.050478
comp10728_c0_seq1	P87033.1	0.037087	0.103962	0.052056	0.057274	0.017621	0.052963	0.058855	0.045778

comp10729_c0_seq1	P87033.1	0.043016	0.123368	0.023197	0.065506	0	0.048251	0.070886	0.011806
comp10747_c0_seq1	Q9Y7K2.2	0.080064	0.076957	0.066264	0.095366	0.077638	0.034556	0.100804	0.061956
comp10748_c0_seq1	Q9Y7K2.2	0.069245	0.057738	0.039824	0.090067	0.051087	0.039082	0.066346	0.041756
comp1077_c0_seq1	Q9UU99.1	0.013606	0.028275	0.004094	0.038788	0.008267	0.018037	0.005837	0.008089
comp10841_c0_seq1	Q06004.3	0.017909	0	0.020096	0	0.008267	0.035482	0.016048	0.033076
comp10854_c0_seq1	O42820.1	2.648457	3.209675	2.084232	4.099351	3.88309	3.611315	3.863698	1.80494
comp10862_c0_seq1	P54872.2	1.352681	0.107467	0.088344	0.142178	1.541488	0.065883	0.1607	0.008416
comp10867_c0_seq1	O74959.1	0.079642	0.00744	0.014637	0.032493	0.039733	0	0.002909	0.006733
comp10877_c0_seq1	Q54WR8.1	0.014426	0.062157	0.019298	0.024397	0.008267	0.027217	0.006062	0.010909
comp10878_c0_seq1	P02723.1	1.578095	0.635267	0.675219	1.520302	2.378466	0.669614	0.9443	1.010068
comp1088_c0_seq1	P78811.2	0.34662	0.6467	0.655558	0.690577	0.305336	1.318172	0.849826	0.915361
comp10881_c0_seq1	Q8LC83.2	0.090693	0.07744	0.028655	0.177384	0.10875	0.089677	0.06181	0.082199
comp10884_c0_seq1	Q6GN02.1	0.558489	0.159113	0.101515	0.165059	1.247818	0.135802	0.122144	0.065453
comp10885_c0_seq1	Q09914.1	0.224908	0.324422	0.301864	0.145853	0.074131	0.150506	0.333916	0.130337
comp10887_c0_seq1	P36551.3	0.069421	0.127544	0.051706	0.076326	0.03473	0.072278	0.148963	0.117855
comp10888_c0_seq1	O42706.2	0.130964	0.179709	0.123677	0.190999	0.105817	0.107315	0.151365	0.074942
comp10890_c0_seq1	Q8CIE6.2	0.219088	0.156555	0.155803	0.477273	0.254244	0.167165	0.268398	0.21794
comp10893_c0_seq1	Q9P324.1	1.433883	2.925966	1.41707	1.287802	1.896021	3.226742	2.147412	0.927121
comp10895_c0_seq1	P08978.1	0.043602	0.025796	0.040742	0.035134	0.013562	0.059692	0.04745	0.017839
comp10900_c0_seq1	Q4P6E9.1	0.146179	0.156714	0.381551	0.405528	0.164899	0.238819	0.783547	0.270079
comp10905_c0_seq1	Q6CBI0.1	0.00541	0.003558	0	0.009741	0	0	0.003153	0.0094
comp10906_c0_seq1	O74905.1	0.577261	0.35315	0.239491	0.611321	0.757091	0.296982	0.197895	0.39727
comp10910_c0_seq1	O14388.2	0.145407	0.204206	0.122998	0.443166	0.208439	0.162605	0.172922	0.19741
comp10912_c0_seq1	P41805.1	0.162868	0.134641	0.094906	0.306296	0.420015	0.167951	0.133173	0.135302
comp10916_c0_seq1	P45443.1	0.014016	0.057981	0.039469	0.079989	0.032508	0.042792	0.046395	0.04075
comp10917_c0_seq1	Q9P7U2.1	0.490907	0.10744	0.062351	0.096291	0.074679	0.070074	0.210105	0.066876
comp10918_c0_seq1	Q9P7U2.1	3.040926	2.348992	1.295823	2.170525	2.612027	1.29779	1.390805	1.173089
comp1092_c0_seq1	Q00333.2	0.018319	0.026119	0.016405	0.046071	0.021035	0.025728	0.013607	0.016834
comp10920_c0_seq1	O74770.1	1.36282	0.947355	0.771939	0.609282	1.035882	0.920243	0.796941	0.286037
comp10921_c0_seq1	P28748.1	0.10127	0.09542	0.088304	0.38348	0.235793	0.245821	0.113261	0.212034

comp10922_c0_seq1	Q8TFH1.1	0.212136	0.467217	0.238168	0.222945	0.140831	0.431615	0.345563	0.239425
comp10931_c0_seq1	Q95VF7.3	0.029139	0.064529	0.046641	0.2196	0.189943	0.159461	0.064161	0.139214
comp10933_c0_seq1	Q92356.1	0	0	0.003907	0	0	0.017686	0.003153	0
comp10934_c0_seq1	O59941.2	0.040817	0.032345	0.032944	0.052587	0.032817	0.006237	0.035678	0.041231
comp10935_c0_seq1	Q9FJX2.1	0.04631	0.076305	0.055958	0.228348	0.100748	0.056222	0.046741	0.106947
comp10936_c0_seq1	Q11118.1	0.082141	0.215741	0.137089	0.147569	0.068209	0.373887	0.158348	0.100628
comp10942_c0_seq1	Q00668.2	0	0.030485	0.002729	0.018311	0	0.042616	0	0.003367
comp10943_c0_seq1	O59675.3	0.231983	0.234285	0.079679	0.181241	0.175587	0.094906	0.124478	0.105176
comp10944_c0_seq1	P52808.2	0.306788	0.336203	0.377097	0.73122	0.939576	0.342377	1.010337	0.717781
comp10946_c0_seq1	Q9UTA8.1	0.139077	0.016336	0.078996	0.08908	0.117592	0.051649	0.064337	0.049189
comp10948_c0_seq1	O74836.1	0.516132	0.386859	0.239477	0.453883	0.916901	0.145483	0.169713	0.415783
comp10950_c0_seq1	Q9P7K6.1	0.00541	0.018168	0.011319	0.033918	0.027125	0.062302	0.024125	0.056884
comp10951_c0_seq1	O42984.1	0.280554	0.091863	0.072918	0.267554	0.209639	0.158908	0.140884	0.128589
comp10966_c0_seq1	P49606.1	0.00541	0.005822	0.014132	0.03146	0.010413	0.005627	0.01023	0.011456
comp10970_c0_seq1	Q99002.1	0.355005	0.676742	0.442353	0.463738	0.307918	0.32643	0.356947	0.822398
comp10971_c0_seq1	P30050.1	0.055119	0.046579	0.070303	0.105382	0.041009	0.070214	0.079476	0.106487
comp10978_c0_seq1	O42700.2	0.603888	2.662436	1.433397	1.034358	0.657764	1.895456	1.057908	1.667552
comp1098_c0_seq1	Q04212.1	0.018033	0.01116	0.002729	0.025302	0.045717	0	0.002928	0.012811
comp10987_c0_seq1	Q9P844.1	0.007213	0.01116	0.003907	0.058536	0.03348	0.083503	0.048981	0.045209
comp10991_c0_seq1	O59768.1	0.00541	0.003881	0.015227	0.032584	0	0.003294	0.01313	0.018342
comp10994_c0_seq1	A1DBW0.1	0.301235	0.261294	0.450287	0.949853	0.390675	0.412425	0.477285	0.545386
comp10996_c0_seq1	P78571.1	0.191352	0.10399	0.101851	0.337	0.429862	0.084623	0.098911	0.223117
comp10997_c0_seq1	P42769.1	0.0315	0.215359	0.133461	0.065681	0.045465	0.223219	0.19263	0.132939
comp10999_c0_seq1	Q9US22.1	0.058223	0.204559	0.126769	0.220854	0.098319	0.291234	0.175269	0.072558
comp11001_c0_seq1	O82204.1	0.075765	0.074236	0.064301	0.236615	0.239869	0.096313	0.119371	0.085895
comp11004_c0_seq1	Q7RVI1.2	0.046352	0.097039	0.090909	0.203227	0.128284	0.116488	0.145723	0.15498
comp11007_c0_seq1	P51997.2	0.057705	0.090782	0.036346	0.139986	0.28947	0.042664	0.037517	0.24336
comp11011_c0_seq1	A5H0J4.1	0.01825	0.07027	0.053718	0.009832	0.010091	0.05026	0.055619	0.071202
comp11018_c0_seq1	Q92250.2	0.434398	0.602613	0.545648	0.57548	0.335897	0.310755	0.574254	0.288288
comp11019_c0_seq1	Q4WCG2.1	0	0.049192	0.013273	0.017115	0.005472	0.047347	0.013738	0.0242

comp11025_c0_seq1	O74893.1	0.082717	0.159729	0.075097	0.106973	0.269018	0.084652	0.04967	0.180858
comp11027_c0_seq1	O74983.1	0.003607	0.023775	0.00482	0.007374	0.022139	0.047763	0.070496	0.021906
comp11028_c0_seq1	Q09785.1	0.039424	0.0169	0.002729	0.025164	0.021256	0	0.004364	0.004722
comp11033_c0_seq1	P0CM47.1	0.056511	0.038897	0.025133	0.061969	0.023874	0.028753	0.034487	0.026956
comp11038_c0_seq1	P36914.2	0.013606	0.126067	0.06048	0.06409	0.023477	0.127433	0.032725	0.031983
comp11039_c0_seq1	Q92196.1	0.019836	0.029866	0.070884	0.132412	0.073796	0.073028	0.081023	0.118358
comp11049_c0_seq1	Q28F67.1	0.110448	0.112373	0.099145	0.229435	0.21077	0.153902	0.136075	0.197673
comp1105_c0_seq1	P0CO12.1	0	0.020808	0.032917	0.058224	0.0356	0.033741	0.037668	0.039874
comp11054_c0_seq1	Q9UT19.1	1.056065	1.28981	0.891204	1.883903	1.929594	1.05506	1.142905	1.625447
comp11059_c0_seq1	O94628.1	0.007213	0.017035	0.054753	0.017928	0.038863	0.039451	0.043214	0.025928
comp11063_c0_seq1	Q92253.2	0.022199	0.052482	0.031074	0.018065	0.01059	0.019397	0.031427	0.008416
comp11064_c0_seq1	Q19QT7.1	0.258817	0.154419	0.15599	0.320813	0.750788	0.100456	0.23166	0.147546
comp11065_c0_seq1	P17576.1	0	0.003881	0.056304	0.024214	0	0.232565	0.043335	0.110161
comp11070_c0_seq1	P49741.1	0.134557	0.276118	0.274564	0.560218	0.233192	0.522218	0.321533	0.423046
comp11072_c0_seq1	P59668.1	0.062456	0.079515	0.056123	0.077239	0.034067	0.090686	0.106612	0.024267
comp11075_c0_seq1	Q6WP50.1	0.284862	0.180114	0.100992	0.068056	0.10716	0.153115	0.199176	0.021228
comp11079_c0_seq1	P24021.2	0.019426	0.007117	0.018598	0.056716	0.007296	0.012299	0.034262	0.050544
comp11081_c0_seq1	Q0USX0.1	0	0.061566	0.021124	0.012153	0.003471	0.088475	0.009093	0.027961
comp11085_c0_seq1	O00061.1	0	0.009381	0	0.020431	0.003471	0.005886	0.002928	0.006733
comp11086_c0_seq1	O60198.1	0.131471	0.26143	0.140067	0.267517	0.259698	0.211066	0.099868	0.11954
comp11088_c0_seq1	Q7Z9I4.1	0.296573	0.227098	0.719176	0.457675	0.928044	0.302728	0.905529	0.652904
comp11098_c0_seq1	Q01085.1	0.039424	0.122562	0.077635	0.233278	0.068329	0.12065	0.091704	0.105831
comp111_c0_seq1	Q1MTQ9.1	0.018033	0	0	0.040725	0.008267	0	0	0.00505
comp11103_c0_seq1	Q2TCH3.1	1.571206	2.303912	1.552976	1.692313	1.86258	2.24679	3.085967	1.046903
comp11111_c0_seq1	P49742.1	0.088167	0.165578	0.152786	0.211093	0.142156	0.366056	0.197515	0.237615
comp11115_c0_seq1	P38972.2	0.097184	0.022534	0.017367	0.087875	0.06578	0.015288	0.036175	0.054261
comp11116_c0_seq1	P56578.1	0.120842	0.642757	0.531603	0.176737	0.140331	0.905579	0.52341	0.202958
comp11123_c0_seq1	Q10305.1	0.301048	0.63616	0.244767	0.490344	0.153982	0.372889	0.21541	0.371885
comp11130_c0_seq1	O93967.1	0.038441	0.022158	0.030826	0.035855	0.029081	0.019749	0.089526	0.044817
comp11131_c0_seq1	Q03666.1	0.163792	0.092239	0.235173	0.256305	0.186366	0.103409	0.195934	0.508786

comp11134_c0_seq1	Q9URW6.1	0	0.019056	0.00859	0.034138	0.003471	0.018102	0	0.038673
comp11136_c0_seq1	P02992.1	0.372471	0.607441	0.273127	0.439641	0.326387	0.372943	0.24681	0.367363
comp11140_c0_seq1	Q0CUL0.1	0.046692	0.065635	0.005458	0.004916	0.051971	0.039803	0.009122	0.003367
comp11144_c0_seq1	Q4WY82.2	0.091936	0.183429	0.073892	0.133562	0.167792	0.141501	0.101649	0.0303
comp11147_c0_seq1	Q03134.3	0.058153	0.009381	0.006636	0.010599	0.041495	0.058015	0.045155	0.218638
comp11151_c0_seq1	Q54TY7.1	0.023318	0.019218	0.012365	0.042325	0.003471	0.024034	0.008971	0.011456
comp11154_c0_seq1	Q00772.2	0.127945	0.402371	0.311506	0.3737	0.191344	0.344385	0.337071	0.190153
comp11155_c0_seq1	P87320.2	0.021515	0.020055	0.021917	0.049004	0.024727	0.018677	0.016404	0.026737
comp11156_c0_seq1	Q9NKW1.1	0.017212	0.10056	0.033958	0.025439	0.012414	0.098363	0.036957	0.012417
comp11157_c0_seq1	Q8X077.1	0.081882	0.272155	0.1531	0.371156	0.08723	0.202191	0.188744	0.191421
comp11164_c0_seq1	P36017.1	0.014426	0.037279	0.044502	0.089729	0.039053	0.065243	0.065523	0.075182
comp11166_c0_seq1	Q9HDG2.1	0.072455	0.23901	0.132739	0.155968	0.096496	0.191199	0.225554	0.086856
comp11168_c0_seq1	Q6RUV5.1	0.049995	0.071403	0.03246	0.021985	0.003471	0.034797	0.052345	0.01515
comp11170_c0_seq1	O42661.1	0.043727	0.162502	0.071014	0.146119	0.042056	0.048373	0.046517	0.04005
comp11175_c0_seq1	P78568.1	0.418734	0.44912	0.539283	0.187728	0.098598	0.820254	0.569768	0.401222
comp11182_c0_seq1	P78774.3	0.017622	0.022966	0.013273	0.099516	0.048702	0.054721	0.024042	0.041253
comp11195_c0_seq1	Q42891.1	0.050203	0.165608	0.103728	0.113698	0.020668	0.267221	0.076601	0.109022
comp11201_c0_seq1	Q8WZY4.1	0.184019	0.043451	0.06224	0.178634	0.128726	0.069482	0.113558	0.134099
comp11204_c0_seq1	P77625.2	0.062704	0.035473	0.051148	0.102357	0.095656	0.027328	0.048449	0.040422
comp11209_c0_seq1	B0YB65.1	0.023318	0.057118	0.045176	0.138742	0.02455	0.062811	0.084061	0.061979
comp11212_c0_seq1	O13819.1	0.009016	0.01682	0.005458	0.043229	0.020006	0.017287	0.004729	0.008067
comp11216_c0_seq1	Q54MV7.2	0.012909	0.011724	0	0.052275	0.007119	0.023378	0.006062	0.017183
comp11221_c0_seq1	P38716.1	0.246798	0.06631	0.112051	0.186833	0.250412	0.168153	0.140695	0.120938
comp11223_c0_seq1	Q09116.2	0.064725	0.099572	0.109476	0.249523	0.13058	0.136015	0.178976	0.152772
comp11224_c0_seq1	C7GPC1.2	0.122971	0.137498	0.270117	0.271105	0.081494	0.151942	0.408116	0.21149
comp11229_c0_seq1	P87035.1	0.016516	0.028195	0.022049	0.087454	0.033391	0.035787	0.073263	0.043133
comp11232_c0_seq1	Q9USQ9.1	0.014016	0	0.002729	0.067106	0.020505	0.015288	0.007292	0.035044
comp11247_c0_seq1	P40566.1	0.016516	0.007117	0.00859	0.04905	0.017797	0.009356	0.016516	0.016178
comp11250_c0_seq1	O94514.1	0.134789	0.079895	0.053208	0.129605	0.094601	0.068011	0.054175	0.040772
comp11251_c0_seq1	C3K630.1	0.038962	0.182943	0.10734	1.487014	0.026771	0.181607	0.114026	0.494952

comp11252_c0_seq1	Q5R885.1	0.266835	0.155664	0.177141	0.514478	0.456872	0.221968	0.202595	0.309714
comp11253_c0_seq1	O74187.1	0.230754	0.130997	0.238662	0.290532	0.248888	0.168842	0.344868	0.288748
comp11254_c0_seq1	O14313.2	0	0	0.013862	0	0	0.02234	0.031802	0.060667
comp11262_c0_seq1	P32390.1	0.082278	0.155311	0.152136	0.183087	0.118692	0.112468	0.102025	0.12894
comp11263_c0_seq1	P28745.2	0	0.045472	0.021274	0.034092	0.004619	0.022997	0.022495	0.01515
comp11264_c0_seq1	Q09859.2	0.102713	0.035258	0.018253	0	0.0407	0.025746	0.020787	0.021883
comp11270_c0_seq1	P90513.1	0.029125	0.039973	0.027592	0.074818	0.034641	0.021396	0.0221	0.05332
comp11281_c0_seq1	O00087.2	0.009016	0.091486	0.123704	0.063907	0.031554	0.229202	0.131343	0.04005
comp11284_c0_seq1	Q9UTJ7.1	0.416537	0.331948	0.393615	0.599369	0.577574	0.388632	0.726652	0.415857
comp11291_c0_seq1	Q00808.1	0.037334	0.053696	0.028823	0.182941	0.035228	0.07658	0.052822	0.119562
comp11292_c0_seq1	P33363.2	0.01082	0.003881	0.004094	0.020386	0.014446	0.007228	0.004364	0
comp11294_c0_seq1	P34559.1	0.00541	0.024475	0.01073	0.110289	0.026581	0.031549	0.028912	0.077676
comp11295_c0_seq1	Q9LT08.1	0.01082	0.060136	0.020822	0.087783	0.034054	0.03761	0.06766	0.082004
comp11298_c0_seq1	Q8MZC4.1	0.003607	0	0	0.009741	0	0	0	0
comp11302_c0_seq1	P47161.1	0.070366	0.160893	0.214534	0.356015	0.123987	0.384898	0.208849	0.286254
comp11303_c0_seq1	Q7XSA2.3	0.011106	0.01116	0.023684	0.039008	0	0.032845	0.046498	0.043439
comp11305_c0_seq1	P78875.2	0.149721	0.06728	0.047501	0.202731	0.239137	0.133355	0.112537	0.154782
comp11314_c0_seq1	O74965.1	0.030656	0.030216	0.052867	0.097232	0.056793	0.047447	0.023686	0.037406
comp11315_c0_seq1	P38988.1	0.098918	0.075501	0.047554	0.131834	0.179826	0.064271	0.106827	0.079183
comp11326_c0_seq1	O49312.1	0.07191	0.028598	0.013256	0.065378	0.107956	0.071668	0.025477	0.05649
comp1133_c0_seq1	Q9C2M6.1	0.007213	0	0	0.009741	0	0	0.003153	0
comp11334_c0_seq1	Q10490.1	0.179458	0.181698	0.095539	0.327895	0.147538	0.152217	0.110678	0.188382
comp11337_c0_seq1	Q0USZ4.1	0.014016	0.005822	0.005861	0.023227	0.016945	0.009051	0.003153	0
comp11340_c0_seq1	Q9P7U2.1	0.044139	0.047762	0.013862	0.009832	0.117158	0.019509	0.009093	0.035634
comp11342_c0_seq1	Q6Z8F5.1	0.00541	0.015283	0	0.017069	0.012887	0	0	0
comp11343_c0_seq1	Q9UW15.2	0.094256	0.217285	0.181255	0.410269	0.143745	0.200524	0.233631	0.258994
comp11347_c0_seq1	O60072.1	0.036979	0.015094	0.026843	0.075639	0.074242	0.034638	0.038504	0.03605
comp11350_c0_seq1	Q9UTT1.2	0.15323	0.31723	0.194869	0.557711	0.1566	0.327793	0.332746	0.342592
comp11355_c0_seq1	Q92429.1	0.123316	0.050299	0.08643	0.18952	0.076786	0.082155	0.085474	0.053911
comp11358_c0_seq1	P0CQ17.1	0.104628	0.06965	0.065457	0.159722	0.12022	0.0741	0.092708	0.121093

comp11367_c0_seq1	O13797.1	0.020122	0.013207	0.054434	0.135754	0.144363	0.081827	0.102797	0.124262
comp11371_c0_seq1	Q5RFT1.1	0	0.020917	0.013729	0.06249	0.006929	0.006589	0.007657	0.017183
comp11372_c0_seq1	P53587.2	0.116838	0.209269	0.066285	0.148157	0.092772	0.138196	0.119883	0.088319
comp11373_c0_seq1	Q8TG12.1	0.01082	0	0	0.018403	0.017033	0	0.003153	0.012133
comp11375_c0_seq1	P48601.2	0.107741	0.121642	0.048276	0.229578	0.154582	0.083779	0.080984	0.109856
comp11378_c0_seq1	P39721.1	0.159673	0.046766	0.047187	0.065086	0.117137	0.104501	0.087633	0.051135
comp11398_c0_seq1	P17608.1	0	0.075254	0.016055	0.01229	0	0.048936	0.026298	0.013466
comp11402_c0_seq1	O74559.1	0.026925	0.033586	0.01932	0.042371	0.022417	0.012105	0.035765	0.019675
comp11406_c0_seq1	Q8J1R4.1	0.047907	0.022804	0.043912	0.063661	0.076521	0.033741	0.066196	0.067684
comp11411_c0_seq1	Q9USP6.1	0	0.045147	0.016002	0.007374	0.004619	0.037324	0.008785	0.01515
comp11413_c0_seq1	P21976.2	0.036066	0.054905	0.016458	0.06104	0.055923	0.031549	0.023808	0.05273
comp11414_c0_seq1	P50085.2	0.018033	0.037897	0.024003	0.057794	0.064195	0.021331	0.040324	0.044117
comp11418_c0_seq1	Q92887.3	0.024411	0.045525	0.034122	0.063707	0.027036	0.019462	0.072145	0.033317
comp11419_c0_seq1	O43102.1	0.111468	0.07736	0.047984	0.210463	0.098704	0.07797	0.070403	0.042456
comp11426_c0_seq1	P53163.1	0.024015	0.033774	0.035136	0.056286	0.01615	0.027217	0.056728	0.030758
comp11433_c0_seq1	P50094.1	0.030656	0.00744	0.014775	0.122605	0.056413	0.028929	0.037761	0.047856
comp11434_c0_seq1	O70595.1	0.038031	0.077601	0.05933	0.081797	0.054091	0.043173	0.064234	0.071706
comp11437_c0_seq1	Q01290.1	0.027376	0.126578	0.075465	0.140461	0.047541	0.056146	0.135108	0.086921
comp11441_c0_seq1	P14065.1	0.127058	0.102646	0.107899	0.24267	0.156367	0.14945	0.153431	0.137139
comp11445_c0_seq1	Q9UUE6.1	0.355712	0.291599	0.165045	0.429949	0.292787	0.209851	0.129799	0.182893
comp11447_c0_seq1	Q9HGN7.1	0.038441	0.031215	0.052906	0.095587	0.053688	0.063326	0.069877	0.068536
comp11451_c0_seq1	O42897.2	0.313362	0.255956	0.162967	0.384522	0.248411	0.167581	0.255994	0.256593
comp11455_c0_seq1	P42769.1	0.030532	0.058007	0.189277	0.119518	0.041142	0.079735	0.412388	0.113223
comp11457_c0_seq1	P32386.2	0.080938	0.270513	0.319673	0.446243	0.066076	0.130811	0.516908	0.25716
comp11464_c0_seq1	Q9P7V2.1	0.103903	0.176199	0.057509	0.2176	0.133553	0.105122	0.143643	0.153733
comp11466_c0_seq1	O94335.1	0.049423	0.052508	0.095725	0.111056	0.052527	0.074518	0.126442	0.097175
comp11477_c0_seq1	Q12458.1	0.057904	0.056201	0.087586	0.142041	0.02474	0.18517	0.11061	0.091621
comp11482_c0_seq1	O43049.2	0.245499	0.060056	0.067326	0.257218	0.204995	0.095034	0.094537	0.103254
comp11484_c0_seq1	Q9P7H3.1	0.053153	0.151483	0.06551	0.128446	0.048411	0.07039	0.104159	0.077106
comp11486_c0_seq1	O94753.1	0	0.003881	0	0	0	0.02992	0.004729	0.008416

comp11491_c0_seq1	Q9P7X0.1	0.134826	0.016982	0.031849	0.066886	0.059266	0.033261	0.025535	0.022584
comp11495_c0_seq1	Q8NBS9.2	0.040941	0.027872	0.017769	0.071327	0.045143	0.026162	0.039328	0.038761
comp11497_c0_seq1	Q96558.1	0.189374	0.248197	0.191042	0.232171	0.142283	0.137105	0.149694	0.098662
comp115_c0_seq1	O94489.1	0.007499	0.018438	0.009552	0.043613	0.014048	0.021525	0.034384	0.021228
comp1150_c0_seq1	Q8CEE6.3	0.017622	0.003558	0	0.014611	0.029359	0	0	0
comp11505_c0_seq1	Q4P7Y8.1	0.069645	0.137171	0.065754	0.09535	0.125223	0.111276	0.09205	0.061059
comp1151_c0_seq1	Q4WVE5.1	0.081349	0.025555	0.084642	0.132438	0.068576	0.091001	0.057632	0.085609
comp11510_c0_seq1	Q56R42.1	0.107004	0.071646	0.069683	0.122568	0.127829	0.060653	0.079315	0.092498
comp11514_c0_seq1	Q9CYR6.1	0.063849	0.058625	0.10645	0.206094	0.110071	0.081698	0.204899	0.164708
comp11519_c0_seq1	P38992.1	0	0	0	0.014611	0.004619	0	0.007545	0.008089
comp11522_c0_seq1	A8N2M6.1	0.003607	0.003881	0.004094	0.033443	0	0.01663	0.007638	0.012133
comp11523_c0_seq1	P0CQ73.1	0.050817	0.127086	0.120831	0.195377	0.081904	0.089816	0.130562	0.169054
comp11532_c0_seq1	O43093.1	0.101459	0.069758	0.0486	0.139729	0.112394	0.050484	0.075064	0.090442
comp11536_c0_seq1	P78980.1	0.014712	0.007763	0.017823	0.043138	0.029068	0.015112	0.033032	0.03452
comp11547_c0_seq1	O50017.1	0.035531	0.054153	0.016591	0.083955	0.070431	0.055583	0.020431	0.035328
comp11552_c0_seq1	Q9UBW8.1	0.024015	0.035338	0.093872	0.109977	0.024152	0.032669	0.111083	0.089612
comp11553_c0_seq1	Q9P7L5.1	0.028728	0.028222	0.018545	0.042279	0.039437	0.012456	0.025487	0.017511
comp11556_c0_seq1	Q9BXS5.3	0.028032	0.02798	0.019617	0.060344	0.019432	0.031438	0.028518	0.027283
comp11561_c0_seq1	P53687.1	0	0.005337	0.011484	0.004824	0	0.052371	0.008727	0
comp11564_c0_seq1	Q13423.3	0.026694	0.042993	0.024083	0.102102	0.05341	0.036268	0.043608	0.085654
comp11565_c0_seq1	P33280.3	0.055012	0.051186	0.020716	0.050083	0.084991	0.027328	0.016506	0.028048
comp11569_c0_seq1	Q304B9.1	0.009016	0.003935	0.046878	0.028527	0.003648	0.040858	0.007516	0.011783
comp11574_c0_seq1	Q6TKS7.1	0.00541	0.106984	0.040896	0.168951	0	0.084992	0.028415	0.120632
comp11576_c0_seq1	P30574.2	0.059135	0.07623	0.10459	0.087454	0.033877	0.169791	0.122802	0.065475
comp11577_c0_seq1	P04843.1	0.11209	0.083885	0.175536	0.199415	0.165955	0.135253	0.344067	0.226425
comp11581_c0_seq1	Q7T3C6.1	0.007213	0.038463	0.014048	0.050128	0.010678	0.047687	0.026961	0.013466
comp11584_c0_seq1	Q9P6N2.1	0.035572	0.01682	0.006823	0.037976	0.025124	0.015464	0.013597	0.020025
comp11586_c0_seq1	Q9P3W1.2	0.009999	0	0.002729	0.020386	0.010413	0	0.00901	0.006733
comp11587_c0_seq1	O60017.1	0.041978	0.11111	0.074747	0.085983	0.064694	0.206237	0.150071	0.209346
comp11588_c0_seq1	O14081.1	0.166425	0.040081	0.017664	0.116539	0.098748	0.073621	0.046376	0.130776

comp11589_c0_seq1	Q870W0.1	0.462372	0.046794	0.095973	0.17775	0.17318	0.040905	0.083654	0.071094
comp11590_c0_seq1	A8NEP3.2	0.138642	0.129598	0.125779	0.292171	0.151976	0.132774	0.155658	0.159721
comp11591_c0_seq1	O14085.1	0.012623	0.007117	0	0.028572	0.008678	0.004941	0	0.012811
comp11597_c0_seq1	Q9UT60.1	0.012499	0.013342	0.006823	0.060819	0.041968	0.019878	0.01676	0.026912
comp11599_c0_seq1	Q12306.1	0	0	0.007815	0.017023	0	0	0	0.013817
comp11600_c0_seq1	Q76PD2.2	0.00541	0.039408	0.011643	0.012153	0.008855	0.023858	0.012245	0.021883
comp11601_c0_seq1	O04421.2	0.00541	0.007493	0	0.014611	0.008855	0	0	0
comp11602_c0_seq1	O59672.1	0.04594	0.039381	0.016777	0.066027	0.04744	0.028864	0.028668	0.048884
comp11608_c0_seq1	P0CO94.1	0.022622	0.051697	0.026094	0.093833	0.017797	0.038971	0.022222	0.071574
comp11612_c0_seq1	P66776.1	0.101798	0.094507	0.235722	0.012061	0.253776	0.023747	0.45998	0
comp11619_c0_seq1	Q4PHN4.1	0.19014	0.1879	0.123746	0.285927	0.132871	0.125897	0.117817	0.1806
comp11624_c0_seq1	P49354.1	0.075378	0.108222	0.072724	0.038788	0.031082	0.065227	0.055844	0.027392
comp11637_c0_seq1	O43670.1	0.003607	0.011644	0.005458	0	0.020492	0.013401	0.018977	0.017861
comp11641_c0_seq1	Q875Q8.1	0.146142	0.112725	0.081863	0.21056	0.0634	0.088686	0.116823	0.067005
comp11656_c0_seq1	Q2UPZ7.1	0.079642	0.034179	0.0717	0.141382	0.103261	0.071908	0.08975	0.088955
comp11659_c0_seq1	Q8LQJ9.1	0.01623	0.016389	0.020685	0.052366	0.021256	0.012855	0.010669	0.028748
comp11663_c0_seq1	O94567.1	0.159162	0.183343	0.115076	0.312008	0.179225	0.13656	0.107263	0.179199
comp11665_c0_seq1	O05220.1	0.026515	0.026631	0.029089	0.056286	0.029169	0.030031	0.028518	0.047264
comp11669_c0_seq1	Q58EB4.1	0.12158	0.070296	0.069931	0.139045	0.045231	0.070185	0.06172	0.082637
comp11675_c0_seq1	P0CM39.1	0.070527	0.01725	0.031982	0.070852	0.02542	0.027439	0.050657	0.027283
comp11677_c0_seq1	Q4P5N0.1	0.086031	0.013262	0.02574	0.062919	0.03329	0.017926	0.031344	0.0209
comp11679_c0_seq1	A8N2Y6.2	0.081678	0.062292	0.070733	0.108398	0.090171	0.059182	0.093044	0.074898
comp11680_c0_seq1	Q8LKS5.2	0.079258	0.067118	0.079235	0.118997	0.039645	0.061885	0.098799	0.069192
comp11681_c0_seq1	Q99128.2	0.022212	0.054717	0.015094	0.074818	0.046468	0.032445	0.035727	0.048795
comp11684_c0_seq1	Q6FUD0.1	0.038155	0.024422	0.017769	0.062665	0.030684	0.046227	0.015091	0.043614
comp11689_c0_seq1	A1D1Z9.1	0.07553	0.186041	0.211095	0.372881	0.082756	0.255951	0.226846	0.220803
comp11690_c0_seq1	Q9UKD2.2	0.00541	0.007763	0	0.017069	0.003648	0	0	0.009772
comp11691_c0_seq1	P87317.1	0.050243	0.073855	0.067012	0.151106	0.041672	0.097902	0.079418	0.119124
comp11694_c0_seq1	Q9UT21.1	0.019426	0.022399	0.020206	0.030555	0.028772	0.003294	0.013597	0.00505
comp11698_c0_seq1	Q4PF85.1	0.285589	0.128545	0.062643	0.171292	0.157555	0.065355	0.103947	0.090397

comp11720_c0_seq1	Q9USS2.1	0.018319	0	0.005458	0.040771	0.023388	0.012345	0.016404	0.012811
comp11723_c0_seq1	P09437.2	0.049423	0.043424	0.036692	0.099087	0.138475	0.039803	0.056981	0.045078
comp11727_c0_seq1	Q6GM14.1	0.036227	0.037979	0.015816	0.112072	0.042909	0.03774	0.014603	0.022342
comp11732_c0_seq1	Q00922.1	0.112989	0.083182	0.020079	0.210206	0.230604	0.144416	0.013363	0.118447
comp11733_c0_seq1	P47154.1	0.016516	0.022561	0.013411	0.061724	0.029655	0.021267	0.019425	0.018539
comp11734_c0_seq1	Q2I6J0.1	0.009016	0.003881	0.003907	0.012199	0.011562	0	0.002909	0.003367
comp11737_c0_seq1	O34389.1	0.012623	0.051079	0.045012	0.082401	0.035405	0.0422	0.055498	0.053669
comp11739_c0_seq1	B0D0N9.1	0.071348	0.118224	0.100576	0.161997	0.092052	0.095468	0.110223	0.094376
comp11743_c0_seq1	P0CL95.1	0.04012	0.022158	0.020366	0.041109	0.023697	0.023923	0.002928	0.012133
comp11754_c0_seq1	P22394.2	0	0.116414	0.126443	0.021985	0	0.143741	0.072971	0.079335
comp11755_c0_seq1	Q9Z1Z0.2	0.068591	0.045852	0.096509	0.152102	0.089774	0.089741	0.15175	0.107954
comp11757_c0_seq1	Q09766.1	0	0.005337	0.00241	0.014657	0.005207	0	0.002909	0.003017
comp11773_c0_seq1	P70584.1	0	0	0.004094	0.027577	0.003648	0.004414	0	0.011456
comp11779_c0_seq1	Q9US41.1	0.033891	0.01116	0.010916	0.051937	0.039557	0.018324	0.01673	0.029667
comp1178_c0_seq1	Q4WWD3.1	0.050082	0.202991	0.08002	0.15476	0.053322	0.135448	0.129616	0.047833
comp11786_c0_seq1	Q9VTF9.1	0.004999	0.014718	0.014637	0	0.003471	0.018453	0.017512	0.032005
comp11787_c0_seq1	O94316.1	0.159598	0.140811	0.077605	0.206826	0.080447	0.108638	0.11263	0.097436
comp11789_c0_seq1	Q4P2E8.1	0.053236	0.018276	0.022825	0.073172	0.077391	0.049057	0.03293	0.039089
comp11791_c0_seq1	Q92373.1	0.003607	0.024098	0.018917	0.083571	0.015784	0.043127	0.050583	0.02595
comp11792_c0_seq1	Q09690.1	0.009016	0.00744	0.012365	0.027714	0.015298	0.012216	0.015174	0.026409
comp11801_c0_seq1	Q8TG24.1	1.631243	0.165936	0.496203	0.193094	2.371889	0.075332	0.479751	0.032136
comp11804_c0_seq1	P78753.3	0.012623	0.0569	0.016756	0.039008	0.028463	0.032845	0.014911	0.020484
comp11813_c0_seq1	P0CM02.1	0.029139	0.016659	0.019458	0.050932	0.042233	0.043496	0.018185	0.042105
comp11819_c0_seq1	P87121.3	0	0.012777	0.006636	0	0.008943	0.012169	0.014837	0.004525
comp11829_c0_seq1	Q7K0E3.1	0.007213	0.037844	0.008001	0.039008	0.006929	0.01377	0.011685	0.040509
comp11830_c0_seq1	O13671.2	0.169936	0.139628	0.106677	0.21695	0.12268	0.105334	0.169601	0.204843
comp11831_c0_seq1	P14964.1	0.003607	0.009381	0.009366	0.044134	0.030406	0.018166	0.009093	0.016178
comp11832_c0_seq1	Q2NL17.1	0.00541	0.007763	0.003907	0.017928	0.003648	0	0	0.003367
comp11835_c0_seq1	Q10752.2	0.01082	0.019003	0.021412	0.050374	0.022417	0.011449	0.013738	0.023392
comp11836_c0_seq1	P47176.1	0.034262	0.024475	0.023041	0.065552	0.062777	0.017686	0.023901	0.0498

comp11837_c0_seq1	O43065.4	0.033783	0.039999	0.029869	0.088396	0.050835	0.024339	0.022616	0.053626
comp11841_c0_seq1	Q10478.1	0.033852	0.027657	0.048595	0.093449	0.060825	0.03601	0.060012	0.0512
comp1187_c0_seq1	Q6NU44.1	0.058277	0.114692	0.153286	0.24683	0.095921	0.134941	0.2603	0.272004
comp1200_c0_seq1	P38918.2	0.050654	0.052858	0.075358	0.094308	0.064504	0.031854	0.091	0.108695
comp1205_c0_seq1	B0XPV4.2	0.010409	0.037494	0.019909	0.044783	0.015784	0.019832	0.020797	0.0101
comp1213_c0_seq1	Q7ZV00.1	0.014426	0.02062	0.009233	0.03793	0.022417	0.020165	0.025272	0.027764
comp1223_c0_seq1	P0CR72.1	0.046978	0.082347	0.037653	0.122451	0.040776	0.065643	0.046556	0.103252
comp1230_c0_seq1	P78814.2	0.164389	0.066683	0.024003	0.134154	0.048204	0.067576	0.027204	0.060993
comp1241_c0_seq1	Q0MQ88.3	0.148014	0.029921	0.011908	0.079385	0.052589	0.024773	0.019435	0.0155
comp1244_c0_seq1	Q9US44.1	0.007499	0.003881	0	0.014657	0	0	0.01552	0.010909
comp1247_c0_seq1	Q10178.2	0.098339	0.11523	0.087945	0.0947	0.066964	0.089324	0.079427	0.035306
comp125_c0_seq1	B1AQJ2.1	0	0	0	0.009741	0	0	0	0.00505
comp1257_c0_seq1	Q96UV5.1	0.279624	0.16124	0.233651	0.374836	0.175586	0.227185	0.22324	0.186544
comp1259_c0_seq1	O93937.2	0.05635	0.063371	0.025824	0.131196	0.148598	0.051029	0.034009	0.067662
comp1261_c0_seq1	P0CM31.1	0.009016	0.020217	0.035322	0.035809	0.019621	0.01639	0.056321	0.048161
comp1267_c0_seq1	Q758S7.1	0.554031	0.126473	0.029169	0.063998	0.349289	0.132568	0.047465	0.033164
comp1268_c0_seq1	Q00472.2	0	0.040026	0.037121	0.007374	0	0.028864	0.031446	0.030299
comp1269_c0_seq1	P45432.2	0.04512	0.139489	0.065218	0.169537	0.02542	0.068526	0.066756	0.070767
comp1274_c0_seq1	O59704.1	0.035531	0.031295	0.03005	0.087409	0.053031	0.046596	0.05559	0.067073
comp1275_c0_seq1	P75791.1	0.020819	0	0.015227	0.022844	0.034641	0.003294	0.005837	0.013139
comp128_c0_seq1	O14295.1	0.061897	0	0.016591	0.017023	0.044454	0	0.020899	0.011783
comp1291_c0_seq1	Q8HXY4.1	0.021105	0.003881	0	0.022844	0.010091	0.010106	0.006062	0.008416
comp1309_c0_seq1	Q9C102.1	0.082443	0.117847	0.065683	0.242149	0.175587	0.081347	0.070277	0.087579
comp1318_c0_seq1	Q04344.2	0.048891	0.043907	0.033958	0.086029	0.113586	0.092091	0.053361	0.071618
comp1320_c0_seq1	Q00737.3	0.038372	0.045633	0.019617	0.033734	0.025389	0.027774	0.010425	0.016483
comp1338_c0_seq1	Q96WP5.1	0.015409	0.003935	0.003907	0.018403	0.004619	0	0	0.003367
comp1344_c0_seq1	O94083.1	0.205453	0.167232	0.236275	0.221437	0.242143	0.387644	0.438433	0.265836
comp135_c0_seq1	Q9XH44.1	0.007213	0	0.012095	0.025731	0.021357	0.009356	0.018102	0.00505
comp1352_c0_seq1	Q6CA53.1	0.049014	0.03914	0.033352	0.117452	0.023464	0.024561	0.073811	0.026278
comp1374_c0_seq1	O14176.1	0.021515	0.030001	0.01718	0.06779	0.049074	0.028624	0.025413	0.017183

comp1376_c0_seq1	P55059.1	0.485492	0.590015	0.638037	0.776856	0.507449	0.447852	0.88617	0.618859
comp1379_c0_seq1	P49348.1	0.049957	0.020702	0.03219	0.101662	0.115459	0.022803	0.046517	0.052337
comp1382_c0_seq1	Q9P7U2.1	0.027497	0.089004	0.041454	0	0	0.005627	0.040231	0
comp1385_c0_seq1	Q4P4Y2.1	0.027211	0.039273	0.037227	0.051299	0.013562	0.064892	0.022222	0.09066
comp1387_c0_seq1	Q6Q0N1.1	0.335656	0.266203	0.253618	0.60017	0.272086	0.316803	0.2928	0.396569
comp1396_c0_seq1	Q12019.1	0.048781	0.022561	0.017664	0.0412	0.063413	0.018277	0.02085	0.016505
comp1402_c0_seq1	P17576.1	0.058127	0.027818	0.198724	0.10179	0.069813	0.069253	0.189627	0.098796
comp1403_c0_seq1	P17576.1	0.058127	0.027818	0.198724	0.10179	0.069813	0.069253	0.189627	0.098796
comp1405_c0_seq1	P07820.4	3.399095	3.702818	5.67768	3.957255	3.873486	6.096779	3.950232	5.295937
comp1408_c0_seq1	Q09172.1	0.014426	0.040269	0.037817	0.102578	0.030684	0.059228	0.035595	0.12509
comp141_c0_seq1	P17576.1	0	0	0.07884	0	0	0.036972	0.064308	0
comp1415_c0_seq1	Q6GLM5.1	0.029835	0.046498	0.048525	0.05137	0.030892	0.022627	0.031558	0.04484
comp1422_c0_seq1	P49010.1	0.014426	0.011321	0.023848	0.042617	0.024241	0.007515	0.004729	0
comp1423_c0_seq1	Q4P965.1	0.028604	0.022642	0.078163	0.097012	0.003648	0.03275	0.066045	0.047505
comp1425_c0_seq1	P41764.2	0.488947	0.436117	0.316103	0.682821	0.419743	0.401456	0.424672	0.371491
comp1430_c0_seq1	O94489.1	0.845867	1.298972	1.29253	1.555272	0.737181	1.078188	1.145761	0.788758
comp1446_c0_seq1	Q4WY82.2	0.039603	0.068414	0.031663	0.07236	0.072564	0.067353	0.031212	0.035022
comp1449_c0_seq1	Q4WY82.2	0.039603	0.068414	0.031663	0.07236	0.072564	0.067353	0.031212	0.035022
comp1456_c0_seq1	Q8J0Q0.2	0.031228	0.030216	0.065297	0.072497	0.036597	0.030382	0.085986	0.061323
comp1459_c0_seq1	P41805.1	0.11	0.059598	0.062356	0.151224	0.338623	0.073509	0.07302	0.055047
comp146_c0_seq1	Q9UUB5.1	0	0.003881	0	0.009741	0.003471	0	0.007273	0.007084
comp1460_c0_seq1	Q5XDZ5.1	0.082264	0.142888	0.175266	0.141182	0.077183	0.17141	0.118433	0.059288
comp1469_c0_seq1	P30598.2	0.022212	0.046901	0.016888	0.026901	0.02308	0.029726	0.019444	0.030584
comp1486_c0_seq1	Q4P0I7.1	0.061392	0.140918	0.146495	0.199551	0.123594	0.078293	0.145649	0.125901
comp1493_c0_seq1	Q9USJ5.2	0.075161	0.117444	0.259605	0.184712	0.146497	0.092167	0.31501	0.21726
comp1499_c0_seq1	Q01752.1	0.114188	0.509955	1.087463	0.441713	0.34533	0.205969	1.08388	0.338704
comp1501_c0_seq1	O94671.1	0.062852	0.022911	0.014319	0.046337	0.057911	0.065114	0.022081	0.060425
comp1503_c0_seq1	P87068.2	0	0.020217	0.063264	0.014473	0.006942	0.064587	0.11223	0.045472
comp1515_c0_seq1	Q962X9.1	0.027211	0.015633	0.015227	0.038405	0.005207	0.008829	0.004393	0.008416
comp1519_c0_seq1	O14104.1	0.016516	0.015256	0.036346	0.049699	0.048601	0.020675	0.04662	0.058655

comp152_c0_seq1	P50125.2	0.052265	0.01682	0.063167	0.057666	0.174442	0.045254	0.129943	0.077958
comp1524_c0_seq1	Q48436.2	0.034575	0.089249	0.082961	0.102715	0.021344	0.177048	0.085387	0.190237
comp1525_c0_seq1	P61962.1	0.003607	0.003558	0	0.035088	0.010413	0.00422	0.002909	0.013139
comp153_c0_seq1	Q69TH4.1	0	0.005902	0.006025	0	0	0.014789	0	0.020309
comp1539_c0_seq1	P0CQ09.1	0.025408	0.00744	0.003907	0.028143	0.006942	0.007228	0	0.017292
comp154_c0_seq1	O14111.1	0	0.016659	0.007868	0.039867	0.007119	0.007515	0.010547	0.013139
comp157_c0_seq1	O60182.1	0.01866	0.02469	0.017637	0.043183	0.027535	0.009356	0.017868	0.028617
comp1601_c0_seq1	O94666.1	0.015409	0.007493	0.00241	0.022752	0.034142	0	0.007545	0.008089
comp1605_c0_seq1	Q3E792.1	0.103384	0.315174	0.167085	0.292208	0.167342	0.166045	0.275612	0.134582
comp1641_c0_seq1	O74478.1	0.076186	0.043289	0.031982	0.110673	0.064208	0.036139	0.040314	0.048489
comp1644_c0_seq1	P28737.2	0.003607	0.027172	0.035508	0	0.0104	0.022756	0.037337	0.003367
comp1655_c0_seq1	Q3SWX2.1	0	0.009219	0.009769	0.025256	0.005207	0.007515	0.011909	0.014975
comp1670_c0_seq1	Q4WRH9.1	0.093833	0.135366	0.069857	0.195981	0.058869	0.110322	0.02394	0.01985
comp1671_c0_seq1	B0XVI5.1	0	0.071379	0.164555	0.020386	0.004619	0.286159	0.12508	0.221718
comp1690_c0_seq1	P41636.1	0.003607	0.032668	0.012095	0.043658	0.003471	0.015944	0.013475	0
comp1694_c0_seq1	P27614.2	0.004999	0.007816	0	0.04905	0.004619	0.009531	0.026868	0.036379
comp1698_c0_seq1	A2AGT5.1	0.039234	0.043504	0.024943	0.062965	0.043275	0.037194	0.043832	0.0101
comp17_c0_seq1	Q9P3U1.2	0.01082	0.012831	0.010411	0.035947	0.019533	0.019684	0.009122	0.021906
comp1723_c0_seq1	Q4PEJ3.1	0.00541	0	0.006636	0.02521	0.015298	0.002814	0.016648	0.028245
comp1724_c0_seq1	Q66X93.1	0.135597	0.099544	0.084703	0.279306	0.257883	0.122104	0.149336	0.182282
comp1736_c0_seq1	Q6DER1.1	0.021925	0	0	0.028189	0.034862	0	0.00899	0
comp1748_c0_seq1	Q12618.1	0.071472	0.061969	0.024003	0.066073	0.031082	0.073545	0.036306	0.024594
comp1750_c0_seq1	Q70J59.1	0.003607	0.003558	0.005139	0.009741	0.008855	0.072501	0.018307	0.080471
comp1770_c0_seq1	P08159.2	0	0.020055	0.004094	0	0	0.025699	0.066788	0.047506
comp1780_c0_seq1	O74945.1	0.027335	0.053073	0.013645	0.084905	0.079329	0.034667	0.071838	0.057584
comp1785_c0_seq1	P30666.2	0.024425	0.05984	0.032487	0.094554	0.026816	0.058895	0.050525	0.050347
comp1795_c0_seq1	O43108.1	0	0	0	0.009741	0.003471	0	0.003153	0
comp1796_c0_seq1	O14295.1	0.063563	0.028706	0.095188	0.091712	0.086038	0.010698	0.133358	0.057278
comp1799_c0_seq1	P04841.1	0	0	0.003615	0	0.009826	0.016871	0.019313	0.011783
comp180_c0_seq1	Q02899.3	0	0.022964	0.009073	0	0	0.023489	0.002909	0.035393

comp1803_c0_seq1	P38795.1	0.03762	0.028356	0.023414	0.048145	0.052439	0.017926	0.031363	0.033689
comp1838_c0_seq1	P53326.1	0.00541	0.003935	0	0.014565	0.003648	0	0.009122	0
comp1850_c0_seq1	Q09877.1	0.009016	0.007117	0.02002	0.033534	0.018769	0.002814	0.025628	0.012811
comp1852_c0_seq1	Q8VE80.1	0	0	0	0.012199	0	0	0	0.003017
comp1860_c0_seq1	O74476.1	0.108276	0.1365	0.067304	0.170092	0.069106	0.075239	0.073644	0.094924
comp1875_c0_seq1	Q17R09.1	0.009999	0.021105	0.01143	0.040725	0.0104	0.013863	0.010566	0.019347
comp1884_c0_seq1	Q32L63.1	0.029997	0.005337	0.004094	0.009741	0.01615	0	0.003153	0
comp1914_c0_seq1	P0CO35.1	0	0.00744	0.008001	0.017023	0.003471	0	0.005837	0.003367
comp1916_c0_seq1	Q9URX7.2	0.037459	0.060163	0.034552	0.121638	0.101614	0.060284	0.044576	0.083598
comp1955_c0_seq1	P14242.3	0.030409	0.024313	0.012635	0.066119	0.037141	0.04145	0.01022	0.044795
comp1987_c0_seq1	O74435.1	0.019015	0.005822	0	0	0	0.014807	0	0
comp1989_c0_seq1	O74885.2	0.155404	0.037414	0.056379	0.158489	0.065325	0.073814	0.072908	0.051987
comp20_c0_seq1	Q5R7H0.1	0.033372	0.022561	0.017234	0.048912	0.028008	0.018582	0.016068	0.033689
comp2010_c0_seq1	O59801.1	0.022622	0.019164	0.01073	0.049991	0.021565	0.010827	0.033061	0.024945
comp2013_c0_seq1	Q9LQV2.1	0.022908	0.045417	0.041002	0.07978	0.021741	0.022498	0.007882	0.056534
comp2014_c0_seq1	Q4R5D9.1	0.014016	0.018761	0.017367	0.04025	0.008267	0.045929	0.024173	0.06412
comp2015_c0_seq1	O14139.1	0.071773	0.067171	0.03433	0.143046	0.039746	0.056146	0.028668	0.050631
comp2016_c0_seq1	P40387.2	0.398671	0.052858	0.100026	0.346807	0.211257	0.148424	0.103566	0.197436
comp2019_c0_seq1	O42641.1	0.115036	0.142259	0.068301	0.12731	0.121536	0.071353	0.054258	0.044597
comp2026_c0_seq1	P39743.1	0.096948	0.065877	0.026546	0.135187	0.047528	0.061644	0.050807	0.026233
comp203_c0_seq1	P53394.1	0.00541	0.003881	0.003907	0.012153	0.005207	0	0.004729	0
comp2034_c0_seq1	Q8TG24.1	0.995502	0.061621	0.150881	0.154606	1.165867	0.050242	0.177076	0.032661
comp2051_c0_seq1	P48510.2	0.019426	0.024582	0.019909	0.072185	0.017696	0.045495	0.02681	0.061889
comp2058_c0_seq1	D8PR70.1	0.026515	0.028328	0.017664	0.093266	0.026462	0.044598	0.019761	0.056731
comp2059_c0_seq1	D8PR70.1	0.012909	0.008896	0	0.023319	0.005207	0.008635	0	0.009772
comp2060_c0_seq1	O14192.1	0.022212	0.027898	0.024189	0.065378	0.01502	0.025441	0.072145	0.0465
comp2061_c0_seq1	Q4PEV8.1	0.157463	0.18146	0.155307	0.189663	0.042069	0.092255	0.127585	0.177953
comp2067_c0_seq1	O14159.1	0.007213	0.031941	0.01073	0.041155	0.011738	0.025921	0.025292	0.013466
comp2069_c0_seq1	Q9Y719.2	0.023729	0.03922	0.01812	0.086413	0.043103	0.049123	0.034477	0.033667
comp207_c0_seq1	P49024.1	0	0.003558	0.006318	0	0	0.013336	0.004393	0.003017

comp2072_c0_seq1	Q70KF8.1	0.055081	0.096416	0.088145	0.076984	0.0769	0.065883	0.050019	0.091686
comp2074_c0_seq1	P40233.2	0.039124	0.020997	0.005458	0.039738	0.026374	0.022211	0.005857	0.00505
comp2075_c0_seq1	P40233.2	0.039124	0.020997	0.005458	0.039738	0.026374	0.022211	0.005857	0.00505
comp2076_c0_seq1	O13848.1	0.059384	0.057981	0.079612	0.121501	0.129988	0.120826	0.195168	0.155436
comp2104_c0_seq1	Q501Z5.1	0	0.003881	0	0.009741	0	0	0	0
comp2105_c0_seq1	Q06816.2	0.269122	0.338875	0.194541	0.460258	0.158244	0.258181	0.327937	0.207642
comp2114_c0_seq1	Q9LZ06.1	0.020463	0.015203	0.003907	0.024397	0.145906	0.02843	0.015174	0.048445
comp2129_c0_seq1	Q9HFU4.2	0.044833	0.044907	0.023924	0.099087	0.04619	0.066521	0.053595	0.076101
comp2136_c0_seq1	O93927.2	0.021229	0.032156	0.018731	0.059311	0.032627	0.013752	0.022475	0.043111
comp2148_c0_seq1	Q9DB16.3	0.051963	0.092458	0.056331	0.14045	0.059178	0.063913	0.106949	0.054589
comp2153_c0_seq1	P32375.2	0.053726	0.045285	0.034419	0.100987	0.038408	0.035213	0.038889	0.044117
comp2175_c0_seq1	Q4P7G1.1	0.003607	0.008896	0.003907	0.009741	0.013474	0.029966	0	0.006034
comp2181_c0_seq1	Q7Z9I4.1	0.027049	0.033048	0.087236	0.137526	0.095511	0.034556	0.138626	0.084233
comp2183_c0_seq1	O42916.1	0.079519	0.0517	0.040759	0.052412	0.049674	0.036619	0.028405	0.013466
comp2188_c0_seq1	Q4QQW3.1	0.067169	0.04488	0.034844	0.02521	0.075032	0.064523	0.018909	0.036377
comp2198_c0_seq1	Q12132.1	0.003607	0.013395	0.008271	0	0.004619	0.011994	0	0.016156
comp2209_c0_seq1	O74341.1	0.040943	0.249298	0.097843	0.140507	0.075752	0.103985	0.066663	0.178017
comp2210_c0_seq1	Q7ZV68.1	0.012623	0.022534	0.023817	0.043229	0.03283	0.022932	0.019332	0.031109
comp2221_c0_seq1	O14351.1	0.038606	0.03744	0.023095	0.079559	0.083843	0.031068	0.022466	0.044423
comp2223_c0_seq1	P32473.2	0.047047	0.069597	0.076803	0.113468	0.055998	0.057424	0.075807	0.074547
comp223_c0_seq1	Q01752.1	0.070079	0.157534	0.566314	0.237753	0.222659	0.105874	0.771682	0.231386
comp2244_c0_seq1	P56329.2	0.021639	0.035473	0.014824	0.098182	0.053732	0.043496	0.025375	0.072537
comp2268_c0_seq1	P53049.1	0.008606	0.042128	0.016002	0.031634	0.003648	0.034621	0.016506	0.0101
comp227_c0_seq1	O94616.1	0.012623	0.003558	0.004094	0.030555	0.005207	0.007755	0.004393	0.008416
comp2275_c0_seq1	P51044.1	0.927276	1.374741	0.903735	0.652595	1.27968	0.803598	0.764687	0.431045
comp2278_c0_seq1	Q09816.1	0.100242	0.197553	0.189331	0.35109	0.125197	0.440499	0.528522	0.253553
comp2281_c0_seq1	P22394.2	0	0.003558	0.008001	0	0	0.015399	0.003153	0.003367
comp2290_c0_seq1	Q9UUM7.1	0.091973	0.124154	0.056309	0.051891	0.026462	0.072796	0.043935	0.034498
comp2294_c0_seq1	P30656.3	0.031924	0.24075	0.105344	0.090041	0.047616	0.142565	0.100916	0.100628
comp2308_c0_seq1	Q5RBE5.1	0.020819	0.04523	0.042331	0.109302	0.040422	0.041496	0.077242	0.092191

comp2314_c0_seq1	C1GD57.1	0.081745	0.101968	0.061182	0.172104	0.082372	0.079106	0.046405	0.080865
comp232_c0_seq1	Q53FA7.2	0.037908	0.018976	0.014824	0.043183	0.034641	0.0252	0.062258	0.034147
comp2335_c0_seq1	P08096.2	0.018033	0.020863	0.006636	0.056899	0.027522	0.015399	0.012245	0.023939
comp2351_c0_seq1	Q9C0W1.1	0.026104	0.028706	0.003907	0.03008	0	0.010458	0	0.013139
comp2358_c0_seq1	Q9UTI3.1	0.007213	0	0	0.023273	0	0.006589	0	0
comp2362_c0_seq1	P41922.1	0.007213	0.003558	0.005861	0.048054	0.018844	0.006237	0.027743	0.021578
comp2369_c0_seq1	P0CM61.1	0.033017	0.064313	0.037732	0.05232	0.035626	0.02122	0.03581	0.034672
comp238_c0_seq1	Q9P7V2.1	0.004999	0.030189	0.017367	0.039867	0.037216	0.016695	0.024125	0.0094
comp2386_c0_seq1	O13923.1	0.090063	0.052643	0.092074	0.217654	0.097644	0.091083	0.204095	0.112982
comp2387_c0_seq1	P48444.1	0.125433	0.047063	0.057328	0.082713	0.105495	0.041831	0.047718	0.043525
comp2401_c0_seq1	Q9USW1.1	0.012623	0.013019	0.019591	0.062236	0.029037	0.037241	0.030216	0.043898
comp2403_c0_seq1	Q08217.1	0.00541	0.003881	0.003907	0.012153	0	0	0	0.0094
comp2404_c0_seq1	P25605.2	0.051474	0.06383	0.046544	0.15644	0.091054	0.051427	0.034282	0.0815
comp2410_c0_seq1	Q00922.1	0.053564	0.020943	0.005861	0.052229	0.0996	0.018277	0.015203	0.008416
comp2415_c0_seq1	B2B3P6.1	0.131523	0.651602	0.504519	0.441008	0.144831	0.810472	0.840854	0.304467
comp2419_c0_seq1	Q75E59.1	0.01082	0.015041	0.063462	0.019481	0.006942	0.173991	0.012123	0.051308
comp2445_c0_seq1	P0CP26.1	0.113028	0.109466	0.073543	0.167634	0.070033	0.078071	0.061559	0.06705
comp2448_c0_seq1	P84675.2	0.012499	0.018679	0.012095	0.049562	0.010413	0.025376	0.007545	0.006733
comp2451_c0_seq1	O94480.1	0.012499	0.011321	0.017637	0.02776	0	0.00422	0	0
comp2455_c0_seq1	P34480.1	0.031638	0.018761	0.019909	0.09832	0.036364	0.032029	0.033164	0.050873
comp2484_c0_seq1	P43069.1	0.014016	0.022588	0.013729	0.040634	0.025389	0.018037	0.005818	0.011456
comp249_c0_seq1	O42709.1	0.008484	0.005902	0.004094	0.035809	0.012401	0.007357	0	0
comp2500_c0_seq1	P38230.1	0.064068	0.031753	0.038135	0.075072	0.065559	0.069547	0.059842	0.107842
comp2519_c0_seq1	Q6PBE2.1	0	0	0.004094	0.034301	0.011915	0.003294	0	0.008416
comp2531_c0_seq1	Q8RY79.1	0.114095	0.028195	0.030826	0.069947	0.079387	0.058607	0.071838	0.044817
comp2546_c0_seq1	O14359.1	0.023443	0.043531	0.019028	0.106232	0.03851	0.030271	0.004729	0.035548
comp2547_c0_seq1	Q09878.2	0.093862	0.03744	0.034179	0.060298	0.092331	0.027153	0.015427	0.037536
comp2555_c0_seq1	O14340.2	0.009999	0.054852	0.03923	0.061248	0.008943	0.035102	0.040436	0.040225
comp2567_c0_seq1	O14227.1	0.025122	0.015094	0.006318	0.045421	0.017342	0.011179	0.016311	0.040401
comp2578_c0_seq1	Q5PNN8.1	0.007213	0.011375	0	0.045687	0.017506	0.022867	0	0.037034

comp2579_c0_seq1	Q4P9T3.1	0.027049	0.011724	0.007709	0.036805	0.022329	0.011003	0.014574	0.01045
comp2580_c0_seq1	Q9HGX4.3	0.293306	0.122368	0.153206	0.595829	0.297564	0.363336	0.149214	0.535269
comp2586_c0_seq1	Q4PIC4.1	0.032676	0.08647	0.031229	0.05667	0.03745	0.056574	0.039347	0.036728
comp2597_c0_seq1	Q05911.1	0.197901	0.070973	0.027321	0.196699	0.179163	0.095561	0.07358	0.102181
comp2608_c0_seq1	Q9V3J1.2	0.219016	0.147123	0.126147	0.266947	0.157603	0.078422	0.158606	0.107449
comp2612_c0_seq1	P54861.1	0.040941	0.041698	0.041029	0.110078	0.069035	0.054564	0.048968	0.041931
comp2616_c0_seq1	Q6BTP9.1	0.058264	0.014718	0.014824	0.044609	0.08845	0.02074	0.016058	0.006733
comp2621_c0_seq1	O60084.1	0.009999	0.011051	0.013384	0	0.012136	0.010458	0.007273	0.003367
comp2642_c0_seq1	Q6DCX2.1	0.004999	0.005337	0	0.032922	0.017608	0.009402	0.010454	0.007084
comp265_c0_seq1	O94524.1	0	0	0	0.012199	0	0	0	0
comp2653_c0_seq1	A3KP37.1	0.012213	0.009381	0.01073	0.035809	0.018844	0.004941	0.010547	0.011456
comp2674_c0_seq1	Q755I4.1	0.012499	0	0	0.02129	0.003471	0	0	0
comp2681_c0_seq1	P59668.1	0	0.003881	0.005139	0.040771	0	0.006108	0	0.013466
comp2689_c0_seq1	P53066.1	0.009016	0.071564	0.034844	0.050466	0.008678	0.029726	0.057625	0.026278
comp269_c0_seq1	Q9UDR5.1	0.023729	0.011321	0.021757	0.032968	0.031744	0.003294	0.049538	0.014822
comp2691_c0_seq1	P00427.1	0.101633	0.093264	0.251653	0.154643	0.089257	0.199313	0.400976	0.179788
comp2702_c0_seq1	P34121.1	0.077837	0.085148	0.047484	0.09067	0.051131	0.070261	0.02376	0.027742
comp2703_c0_seq1	Q7PCJ8.1	0.027621	0.016659	0.016861	0.038405	0.005472	0.013817	0.018336	0.013466
comp2742_c0_seq1	O75746.2	0.143326	0.071994	0.04207	0.076023	0.078225	0.040507	0.048561	0.052337
comp2756_c0_seq1	P22142.2	0.088207	0.056793	0.056521	0.110023	0.096994	0.063467	0.087796	0.079009
comp276_c0_seq1	O13848.1	0.01082	0.020217	0.021009	0.053828	0.036465	0.048742	0.113475	0.066328
comp2765_c0_seq1	P80576.2	0.050764	0.02841	0.006823	0.058361	0.071208	0.028143	0.01979	0.058917
comp2772_c0_seq1	P0CN17.1	0.012623	0.094453	0.146646	0.142562	0.045969	0.027727	0.091032	0.109615
comp2778_c0_seq1	P54007.1	0.021229	0.061319	0.048015	0.073356	0.026771	0.03673	0.043506	0.039044
comp2795_c0_seq1	O13302.1	0.160432	0.125983	0.05855	0.182603	0.166798	0.066715	0.089584	0.096148
comp2798_c0_seq1	Q09923.1	0.004999	0.00744	0.075438	0.039867	0.01521	0.042101	0.121255	0.0404
comp2800_c0_seq1	P80324.1	0.04594	0.020379	0.022001	0.042754	0.053971	0.021045	0.019557	0.025272
comp2809_c0_seq1	P0CN07.1	0.047552	0.058949	0.021863	0.095249	0.02308	0.027411	0.043365	0.070744
comp2814_c0_seq1	O42939.1	0.037265	0.02054	0.043169	0.068439	0.008091	0.038331	0.059778	0.045953
comp2829_c0_seq1	Q9USR3.1	0.018033	0.02806	0.017956	0.066931	0.033979	0.010522	0.026849	0.027414

comp2832_c0_seq1	A6ZRD1.1	0.022908	0.120492	0.102588	0.076472	0.014446	0.19236	0.11377	0.101437
comp2833_c0_seq1	C8VDQ4.1	0.031514	0.019191	0.004094	0.035901	0.009826	0.009402	0.003153	0.017314
comp2840_c0_seq1	Q02899.3	0.007213	0.280834	0.510217	0.20331	0.033303	0.330244	0.474576	0.507075
comp2841_c0_seq1	Q84QK0.1	0.027787	0.045365	0.073144	0.101836	0.057063	0.047587	0.113777	0.071685
comp2847_c0_seq1	Q00614.1	0.20562	0.03779	0.018545	0.067744	0.152026	0.026367	0.025496	0.01445
comp2849_c0_seq1	O94142.1	0	0.016336	0.002729	0	0	0.02533	0.002909	0.008089
comp2858_c0_seq1	O74782.1	0	0	0.005861	0.014565	0	0	0.002928	0.003367
comp289_c0_seq1	O43051.1	0.007213	0.003881	0	0.018403	0.008855	0	0.002909	0.00505
comp2893_c0_seq1	Q5ZLG0.1	0.014712	0.00744	0.011908	0.02034	0.041716	0.007515	0.007292	0.003367
comp2899_c0_seq1	O31826.1	0.021639	0.009381	0.006823	0.033489	0.030507	0.018277	0.038767	0.0256
comp290_c0_seq1	Q54EW1.1	0.157286	0.086605	0.044772	0.253209	0.175149	0.051649	0.043457	0.069541
comp2900_c0_seq1	Q09692.1	0.068724	0.07938	0.024406	0.070943	0.043584	0.045606	0.032676	0.04792
comp2907_c0_seq1	P29251.1	0.012213	0.005337	0.005458	0.025685	0.018668	0.004941	0	0.01045
comp2911_c0_seq1	A8P006.1	0	0	0	0	0	0.011929	0	0.003367
comp2916_c0_seq1	Q9Y8B5.1	0.394564	0.229547	0.293089	0.346346	0.35163	0.190623	0.325216	0.226225
comp2920_c0_seq1	P78567.3	0.408758	0.22731	0.258379	1.113127	0.857168	0.287884	0.266728	0.490924
comp2940_c0_seq1	Q1LXK4.2	0.022908	0.034179	0.014637	0.060507	0.032994	0.017861	0.024081	0.024594
comp296_c0_seq1	O94350.4	0.175172	0.096739	0.088605	0.152394	0.180413	0.065401	0.122417	0.080976
comp2961_c0_seq1	Q6FRC6.1	0.019836	0.005337	0.03503	0.134593	0.040126	0.031327	0.059535	0.097722
comp297_c0_seq1	Q9P7U2.1	0.056884	0.007117	0.15513	0	0.039243	0.024626	0.250157	0.021883
comp2971_c0_seq1	P41003.2	0.029125	0.018976	0.026732	0.058699	0.025787	0.039146	0.021255	0.030803
comp2976_c0_seq1	O74762.1	0.332311	0.283964	0.215545	0.393238	0.37881	0.223265	0.268457	0.29522
comp2977_c0_seq1	O27051.1	0.070079	0.065715	0.053464	0.20571	0.109917	0.048308	0.08976	0.148421
comp2981_c0_seq1	P41004.2	0.029014	0.015094	0.023981	0.079697	0.022814	0.028975	0.032686	0.037711
comp2983_c0_seq1	Q10068.1	0.060653	0.120917	0.059733	0.259495	0.05129	0.116892	0.079418	0.20504
comp2990_c0_seq1	P82934.2	0.06325	0.042966	0.049725	0.161751	0.093276	0.109666	0.049587	0.098245
comp2994_c0_seq1	A1CP08.1	0.008606	0.028195	0.183219	0.059823	0.005207	0.069658	0.125224	0.04545
comp3008_c0_seq1	P87157.1	0.009016	0.033964	0.046833	0.06409	0.031833	0.048625	0.043009	0.023217
comp3011_c0_seq1	Q9P7G4.1	0.036924	0.011375	0.018545	0.031031	0.01562	0.007884	0.004393	0.007739
comp3020_c0_seq1	Q9P7V2.1	0.066459	0.083262	0.062489	0.180801	0.084832	0.057664	0.103786	0.073915

comp3022_c0_seq1	Q9P7V2.1	0.032909	0.024179	0.020096	0.084035	0.021534	0.015223	0.025618	0.042434
comp304_c0_seq1	Q5KPJ5.1	0.069671	0.044719	0.040275	0.086842	0.089901	0.043256	0.040568	0.085895
comp3046_c0_seq1	P28584.1	0	0	0	0	0.008267	0.009051	0.003153	0.004525
comp3052_c0_seq1	O42923.1	0	0.014933	0.005861	0.062144	0.024241	0.026959	0.013485	0.017642
comp3053_c0_seq1	Q9P7U2.1	0.803275	0.505727	0.259963	0.364876	0.459133	0.366994	0.268812	0.183899
comp3055_c0_seq1	O60016.2	0.00541	0.028946	0.006823	0.004824	0	0.025746	0.008971	0.014625
comp3059_c0_seq1	A1CMP1.1	0.241933	0.214396	0.385364	0.556749	0.390504	0.385245	0.429283	0.529623
comp3071_c0_seq1	A8NEG8.3	0.009999	0	0.006636	0.033918	0.014048	0.002814	0.006062	0.0101
comp3074_c0_seq1	Q0VZ69.1	0	0.016982	0.007868	0.049096	0	0.014297	0.030162	0.0101
comp3086_c0_seq1	P62318.1	0.003607	0.052885	0.059296	0.036422	0.030052	0.078433	0.075583	0.017861
comp3094_c0_seq1	Q53552.1	0.03762	0.041672	0.064194	0.024397	0.023388	0.015399	0.065606	0.008089
comp3095_c0_seq1	P23262.4	0.003607	0.071296	0.069466	0	0.003648	0.01377	0.110201	0
comp3096_c0_seq1	Q53552.1	0.00541	0.114962	0.083785	0	0.005472	0.036268	0.128162	0
comp3101_c0_seq1	Q53552.1	0.03762	0.041672	0.064194	0.024397	0.023388	0.015399	0.065606	0.008089
comp3110_c0_seq1	Q8TG11.1	0.016516	0.003558	0.008187	0.055683	0.027036	0.009051	0.019669	0.008416
comp3111_c0_seq1	Q9H6T3.2	0.003607	0	0	0.023227	0	0.006108	0.007292	0
comp3119_c0_seq1	Q9UVT8.1	0.009999	0.07407	0.077853	0.007374	0.005207	0.037564	0.0358	0.025534
comp312_c0_seq1	O94653.2	0.012909	0.005902	0.00859	0.031506	0.020492	0.00422	0.009122	0.003367
comp3126_c0_seq1	O94641.1	0.103534	0.118655	0.056415	0.118751	0.118899	0.070871	0.045004	0.039089
comp3137_c0_seq1	O13713.1	0	0.01116	0.003615	0.040817	0.0104	0.021156	0.022456	0.0256
comp314_c0_seq1	P21734.1	0.00541	0.020702	0.013459	0.04068	0.017329	0.025394	0.027947	0.007084
comp3153_c0_seq1	P43619.1	0.033838	0.022319	0.024646	0.067873	0.036774	0.034908	0.049538	0.038061
comp3165_c0_seq1	O13985.2	0.009016	0	0.00241	0.043475	0.003648	0.030623	0.008747	0.014822
comp3166_c0_seq1	O42963.1	0.041227	0.038167	0.020871	0.073647	0.053795	0.033325	0.042144	0.080079
comp3177_c0_seq1	Q10343.2	0.034057	0.018841	0.00859	0.044134	0.033581	0.017732	0.004364	0.006383
comp3194_c0_seq1	O42870.3	0.041762	0.074881	0.053043	0.223758	0.126296	0.06961	0.078085	0.154608
comp3204_c0_seq1	O60162.1	0.031924	0.037602	0.00859	0.047625	0.022814	0.018582	0.012245	0.013466
comp3209_c0_seq1	Q07E00.3	0.009999	0.009219	0.018545	0.047021	0.003471	0.033131	0.020909	0.015784
comp323_c0_seq1	P36619.2	0.086091	0.160617	0.216764	0.217909	0.139872	0.107944	0.205679	0.159328
comp3249_c0_seq1	Q63615.1	0.009016	0.0131	0.01073	0.035426	0.021565	0.010809	0.028322	0.006733

comp3256_c0_seq1	P46592.2	0.003607	0.01116	0.009769	0.027622	0	0	0.005837	0
comp3257_c0_seq1	Q42966.1	0.081102	0.038005	0.027459	0.069573	0.073358	0.054323	0.05379	0.077871
comp3259_c0_seq1	Q9USZ2.2	0.022622	0.241838	0.113773	0.062665	0.015974	0.112849	0.078309	0.064709
comp3260_c0_seq1	P68209.1	0.057581	0.046579	0.048968	0.129725	0.102997	0.056638	0.079412	0.055618
comp3267_c0_seq1	A4XTE7.1	0.110237	0.042643	0.032966	0.106578	0.047925	0.052529	0.048177	0.067793
comp3276_c0_seq1	Q42649.2	0.158666	0.049783	0	0.019481	0.045831	0.010652	0.010445	0.014822
comp3277_c0_seq1	Q9US35.1	0.007213	0.019029	0.036489	0.052495	0.010413	0.042376	0.033999	0.068973
comp3318_c0_seq1	O48707.1	0.039916	0.080462	0.02791	0.128901	0.026184	0.12337	0.048773	0.052511
comp3328_c0_seq1	Q2UKD0.1	0.066143	0.734609	0.829477	0.281972	0.07206	0.913692	0.348511	0.428305
comp3329_c0_seq1	B8M044.1	0.022908	0.005337	0.015227	0.009741	0.015197	0.035594	0.018214	0.031678
comp3348_c0_seq1	A7EXH9.1	0.04053	0.131317	0.112808	0.044042	0.049175	0.066234	0.073166	0.021183
comp3351_c0_seq1	Q9UT88.1	0.037963	0.079999	0.038916	0.046025	0.053309	0.042025	0.045908	0.027961
comp3382_c0_seq1	Q4P358.2	0.107455	0.145419	0.116334	0.318245	0.170574	0.140734	0.15329	0.239101
comp3395_c0_seq1	O14077.1	0.022622	0.063371	0.027353	0.101187	0.036554	0.046643	0.063136	0.043462
comp3396_c0_seq1	Q24K02.1	0.022622	0.063371	0.027353	0.101187	0.036554	0.046643	0.063136	0.043462
comp3406_c0_seq1	B2RZL5.1	0.112289	0.081729	0.080923	0.200594	0.056678	0.091786	0.123007	0.114555
comp3408_c0_seq1	Q16HW7.1	0.153105	0.112215	0.097381	0.250977	0.068417	0.102003	0.139542	0.151873
comp3419_c0_seq1	P40032.1	0.035941	0.116308	0.036081	0.092674	0.022505	0.248002	0.03762	0.121965
comp342_c0_seq1	P0CL89.1	0.026925	0.088331	0.024592	0.03168	0.013871	0.057729	0.033154	0.04462
comp3427_c0_seq1	O94739.3	0.061035	0.058302	0.052385	0.293955	0.039185	0.067817	0.056859	0.402532
comp3428_c0_seq1	O94561.1	0.024425	0.021481	0.012365	0.05275	0.039159	0.054176	0.012245	0.042018
comp3429_c0_seq1	A7Z026.1	0.00541	0.009704	0.009476	0.009741	0.00912	0	0	0.009051
comp3430_c0_seq1	O36027.3	0.009016	0.018976	0	0.021939	0.004619	0	0	0
comp3446_c0_seq1	P78810.2	0.009016	0.005822	0.011722	0.027806	0.011562	0.003294	0.013139	0.014167
comp3455_c0_seq1	Q54DY9.1	0.003607	0.003558	0.003615	0.012199	0.003471	0	0.003153	0.009772
comp3456_c0_seq1	P30675.3	0	0.042966	0.028048	0.029185	0.015298	0.0252	0.025423	0.014472
comp3459_c0_seq1	Q9FS88.1	0.102727	0.014718	0.05023	0.060032	0.124363	0.009051	0.04068	0.0418
comp347_c0_seq1	Q553X7.1	0.197518	0.148433	0.060668	0.230875	0.229642	0.10228	0.100326	0.096893
comp3471_c0_seq1	P78621.2	0.010409	0	0	0	0	0.015223	0.006081	0.003367
comp3491_c0_seq1	Q5PXQ6.1	0.016516	0.055175	0.140308	0.037317	0.027624	0.125307	0.118146	0.08233

comp3494_c0_seq1	Q5PXQ6.1	0.028728	0.050699	0.150104	0.052403	0.037538	0.134598	0.099652	0.116543
comp3511_c0_seq1	O59760.1	0.031924	0.018976	0.008187	0.055857	0.056206	0.027504	0.026849	0.008416
comp3512_c0_seq1	O59760.1	0	0.003558	0.006823	0.012153	0.003471	0	0.010454	0
comp3524_c0_seq1	Q8BMB3.1	0.007213	0.005337	0.002729	0.03263	0.003648	0.009051	0	0.003367
comp3525_c0_seq1	Q9Y8G7.1	0	0.018599	0.00241	0	0.005472	0.018277	0.004364	0
comp3554_c0_seq1	Q10421.1	0.034996	0.008896	0.019134	0.031863	0.003648	0.037564	0.013334	0.058151
comp3558_c0_seq1	Q5BC69.1	0.004999	0.003935	0	0.019481	0.005207	0	0.003153	0.008067
comp3563_c0_seq1	Q32LB0.1	0.00541	0.016874	0.006636	0.033534	0.012887	0.014918	0.00899	0.02759
comp359_c0_seq1	Q9HFW2.1	0.030532	0.018276	0.027507	0.096583	0.04513	0.05784	0.067426	0.067093
comp3601_c0_seq1	O14134.1	0.044709	0.063098	0.063894	0.112235	0.067622	0.042774	0.052486	0.041603
comp3612_c0_seq1	P30574.2	0.059135	0.07623	0.10459	0.087454	0.033877	0.169791	0.122802	0.065475
comp3627_c0_seq1	P32386.2	0.024589	0.036011	0.009955	0.06801	0.028375	0.019573	0.004393	0.03465
comp3651_c0_seq1	O14002.1	0.018538	0.007493	0.014159	0.044179	0.035127	0.015048	0.027195	0.024922
comp366_c0_seq1	O14250.1	0.083193	0.075124	0.060287	0.249692	0.159542	0.165618	0.158737	0.150014
comp3689_c0_seq1	A7EZP8.1	0.018319	0.003881	0	0.023319	0.020403	0.007515	0.005837	0.012133
comp3696_c0_seq1	B0Y3M6.1	0.088615	0.040134	0.040534	0.107273	0.093713	0.089934	0.01673	0.120195
comp3698_c0_seq1	Q53552.1	0	0	0	0.009741	0	0	0.003153	0
comp375_c0_seq1	Q9LQV2.1	0.008606	0	0	0.015515	0	0	0	0
comp3758_c0_seq1	P40377.1	0.043316	0.039596	0.005458	0.075977	0.082288	0.024163	0.016516	0.043111
comp3769_c0_seq1	P34127.2	0.004999	0	0.002729	0.014611	0.007119	0	0.005837	0.012811
comp381_c0_seq1	O93927.2	0.029835	0.012831	0.009955	0.03051	0.02099	0.00857	0.002909	0.0155
comp3811_c0_seq1	Q09799.1	0.004999	0	0.007231	0.021939	0.004619	0	0.007273	0
comp3818_c0_seq1	O14206.2	0.007213	0.01488	0.002729	0.031414	0.011549	0.005757	0.022392	0.011106
comp3827_c0_seq1	Q9Y7Q2.1	0.09132	0.091188	0.249091	0.289877	0.031934	0.141589	0.173873	0.447487
comp383_c0_seq1	Q9P7S2.1	0.265054	0.251705	0.22436	0.415384	0.306383	0.148923	0.313616	0.270408
comp3832_c0_seq1	Q9UUD8.1	0.035245	0.03922	0.034397	0.075247	0.044834	0.035852	0.034964	0.033034
comp3841_c0_seq1	Q09140.2	0.04773	0.049515	0.025075	0.073089	0.058427	0.023923	0.020636	0.040028
comp3895_c0_seq1	O00061.1	0	0	0	0.015515	0	0.002814	0	0
comp3949_c0_seq1	Q9UT00.1	0.019426	0.109896	0.117805	0.063406	0.058043	0.074758	0.106578	0.034717
comp395_c0_seq1	Q06816.2	0.00541	0.092589	0.210724	0.016374	0.007119	0.07869	0.443907	0.00505

comp3951_c0_seq1	O74959.1	0	0.00744	0	0.020815	0	0.002943	0.002928	0.012133
comp3984_c0_seq1	Q09851.2	0.00541	0.003558	0.022346	0.057182	0.019343	0.023026	0.040183	0.01045
comp4017_c0_seq1	O00061.1	0.009999	0	0.003907	0.019527	0.015974	0	0.010454	0.015609
comp4026_c0_seq1	O14086.1	0.017622	0.003558	0.005861	0.023748	0.020681	0.004941	0.004729	0.014495
comp4039_c0_seq1	P12753.1	0.018319	0.044934	0.02877	0.087583	0.034244	0.041609	0.030084	0.035153
comp4067_c0_seq1	Q70GH4.1	0.00541	0.035473	0.025483	0.007374	0.004619	0.194326	0.019274	0.081805
comp409_c0_seq1	P77526.1	0.028318	0.049083	0.063313	0.121501	0.047925	0.068262	0.095578	0.106641
comp411_c0_seq1	O74455.1	0.118834	0.094018	0.090478	0.240778	0.167103	0.095357	0.138584	0.131871
comp4134_c0_seq1	O74839.1	0	0.003935	0.004094	0	0.003648	0.01639	0.006305	0.003367
comp4136_c0_seq1	P15454.2	0.003607	0.0131	0.010916	0.0518	0.022505	0.016279	0.01025	0.021228
comp414_c0_seq1	Q9SK39.1	0.014426	0.022749	0.014048	0.073155	0.02308	0.035213	0.003153	0.071182
comp4144_c0_seq1	Q6TGC6.1	0	0	0.004094	0.022752	0	0.007515	0.012274	0.006733
comp4163_c0_seq1	O74493.1	0.036103	0.242487	0.338524	0.009649	0	0.469018	0.248047	0.049539
comp4167_c0_seq1	Q9Y5K5.3	0.010409	0.039758	0.023817	0.057749	0.006942	0.031678	0.019537	0.034956
comp4180_c0_seq1	Q99042.1	0.009016	0.016982	0.009233	0.048437	0.019343	0.031549	0.014603	0.019347
comp4189_c0_seq1	P0CO89.1	0.003607	0.005822	0.002729	0.012199	0.003648	0	0.004729	0
comp4194_c0_seq1	Q8NHP1.6	0	0.135447	0.075141	0	0	0.052529	0.191843	0
comp4214_c0_seq1	P33677.2	0.074955	0.008896	0.056893	0.122302	0.040908	0.085807	0.20575	0.582661
comp4216_c0_seq1	P10961.1	0.014712	0.022642	0.011319	0.046454	0.005472	0.022803	0.007302	0.009772
comp4225_c0_seq1	O23461.1	0	0	0.00241	0.012153	0	0	0.002928	0.003367
comp4226_c0_seq1	Q12068.1	0.12621	0.137173	0.267318	0.202056	0.088158	0.091516	0.379885	0.071422
comp4239_c0_seq1	Q8L5U0.2	0.123584	0.137876	0.083306	0.145578	0.044644	0.064142	0.11914	0.188142
comp4275_c0_seq1	O59805.1	0.016105	0.09806	0.032815	0.167388	0.016168	0.05845	0.028732	0.044094
comp4292_c0_seq1	Q9US55.1	0.065652	0.05674	0.117457	0.127484	0.078508	0.10786	0.124558	0.084845
comp4294_c0_seq1	P56272.1	0	0	0.002729	0	0	0.010698	0.002909	0.007084
comp4299_c0_seq1	Q06330.3	0.088125	0.016659	0.031712	0.147592	0.167006	0.050641	0.061954	0.08137
comp4309_c0_seq1	Q07505.1	0.099191	0.060919	0.224545	0.22712	0.187797	0.296008	0.472839	0.380133
comp431_c0_seq1	Q9FEF8.1	0.051269	0.058142	0.0341	0.093137	0.11628	0.068538	0.031933	0.037667
comp4328_c0_seq1	Q96V64.1	0	0.007816	0.007231	0.035901	0	0.002943	0	0.003367
comp4339_c0_seq1	P78586.1	0.421993	0.161405	0.104235	0.368941	0.219074	0.160289	0.16901	0.211533

comp436_c0_seq1	Q00808.1	0.019712	0.051889	0.049057	0.107228	0.020006	0.067085	0.046863	0.088627
comp4362_c0_seq1	Q15427.1	0.00541	0.003881	0	0.017023	0	0	0	0.003367
comp4367_c0_seq1	P42041.2	0.086222	0.037764	0.052751	0.062236	0.049303	0.036361	0.057269	0.096016
comp4376_c0_seq1	O94718.2	0.01623	0.009757	0.003907	0.023227	0.003471	0.004414	0.013597	0.008089
comp4384_c0_seq1	Q6URB0.1	0.384739	0.582338	0.822979	0.436692	0.430827	0.545574	0.511633	0.230703
comp4391_c0_seq1	Q70GH4.1	0	0.016982	0.033803	0.019481	0.005207	0.082114	0.034365	0.097327
comp44_c0_seq1	P53488.1	0.325672	0.135394	0.125743	0.250181	0.162501	0.095591	0.10813	0.124
comp4409_c0_seq1	O13779.4	0.007213	0.093643	0	0.02341	0.005207	0.146888	0.057235	0
comp4411_c0_seq1	Q3UXZ9.2	0.026515	0.026496	0.017664	0.046071	0.021079	0.027458	0.016272	0.029623
comp4424_c0_seq1	Q9Y7J5.1	0	0	0	0.009741	0	0	0	0
comp4442_c0_seq1	Q94A97.1	0.026721	0.128679	0.031415	0.031726	0.01971	0.098616	0.028152	0.040509
comp4443_c0_seq1	P15245.3	0.007499	0.040864	0.04362	0.014657	0.005472	0.003294	0.070335	0
comp4444_c0_seq1	P83778.2	0.023018	0.219392	0.240549	0.128491	0.057782	0.371267	0.328271	0.158474
comp4445_c0_seq1	P49602.1	0.026925	0.036199	0.052587	0.053399	0.023181	0.021636	0.067611	0.006733
comp4448_c0_seq1	P06774.1	0	0.003935	0	0.009741	0	0	0	0
comp4462_c0_seq1	Q8LPJ4.1	0.056225	0.098383	0.028207	0.07638	0.043231	0.075191	0.028293	0.044794
comp4468_c0_seq1	P23506.3	0.014712	0.003881	0.003907	0.013149	0.036377	0	0.006305	0.00505
comp4481_c0_seq1	A6NDR6.2	0.018033	0.003881	0	0.058616	0.020226	0.013641	0.014866	0.030345
comp4485_c0_seq1	Q4A0Q1.1	0.026228	0.165964	0.442046	0.267425	0.010413	0.456142	0.602321	0.196426
comp449_c0_seq1	P50580.3	0.103645	0.151724	0.081863	0.277973	0.142919	0.107695	0.109375	0.134207
comp4490_c0_seq1	Q9Y8G7.1	0.044178	0.0355	0.009073	0.058141	0.015872	0.024274	0.002928	0.006733
comp4504_c0_seq1	O13739.1	0.135854	0.047036	0.042526	0.107528	0.093744	0.042727	0.048074	0.082439
comp4523_c0_seq1	B8NW36.1	0.016516	0.023369	0.052442	0.034347	0.041438	0.066604	0.040324	0.070962
comp4526_c0_seq1	Q9Z0P5.1	0.008606	0.04523	0.051232	0.014657	0.006942	0.066732	0.025458	0.013466
comp4549_c0_seq1	Q5B4Z3.2	0.010287	0.012939	0.014319	0.020386	0.008091	0.003294	0.004364	0.015172
comp456_c0_seq1	O59827.1	0.01623	0.107409	0.056818	0.060344	0.043919	0.047523	0.008727	0.130708
comp457_c0_seq1	Q10133.1	0	0.020379	0.014048	0.033489	0.005207	0.016519	0.010474	0.01515
comp4583_c0_seq1	Q9C0Z7.1	0.1842	0.068627	0.048853	0.15594	0.117265	0.228072	0.04134	0.070087
comp4604_c0_seq1	P27526.1	0.021105	0	0.017367	0.043996	0	0.00422	0.020899	0.015806
comp4616_c0_seq1	Q8BL99.2	0.008606	0.052535	0.036621	0.018786	0.006942	0.039093	0.036053	0.009772

comp4621_c0_seq1	P0CM93.1	0	0.005337	0	0.009741	0.003648	0	0.003153	0
comp4641_c0_seq1	Q9UBJ2.1	0	0.003558	0.003615	0.009741	0	0	0.004393	0.003367
comp4651_c0_seq1	Q08645.1	0.034138	0.028356	0.011962	0.079313	0.093055	0.019333	0.055273	0.024267
comp4659_c0_seq1	P38811.1	0.126046	0.082456	0.04172	0.154923	0.11931	0.079254	0.078095	0.109461
comp4667_c0_seq1	Q10211.1	0.027497	0.00744	0.012684	0	0.008855	0.012169	0	0
comp4677_c0_seq1	P0CR77.1	0	0.009381	0	0	0	0.010522	0.007292	0
comp468_c0_seq1	P42535.4	0.012623	0.189058	0.138697	0.202093	0.039261	0.104085	0.206782	0.056709
comp4706_c0_seq1	Q7G6K7.2	0.008606	0.020943	0.008001	0.045687	0.010767	0.017621	0.007302	0.028966
comp4730_c0_seq1	P32816.1	0.065814	0.138597	0.037689	0.042846	0.04766	0.128722	0.06566	0.09794
comp4751_c0_seq1	Q9P4T7.1	0.029835	0.03585	0.005458	0.032968	0.032331	0.01265	0.025881	0.011456
comp4755_c0_seq1	Q9P7I2.1	0.121796	0.195417	0.096903	0.146107	0.054293	0.087502	0.106927	0.091315
comp4756_c0_seq1	Q9UTG1.1	0.032745	0.032453	0.023414	0.090296	0.07028	0.040184	0.073215	0.026956
comp4778_c0_seq1	Q96TS6.1	0.007499	0.013315	0.019006	0.004824	0	0.047874	0.062619	0.083423
comp4780_c0_seq1	Q96TS6.1	0.011106	0.011724	0.011165	0	0.003648	0.109019	0.077983	0.1299
comp4869_c0_seq1	O13991.3	0.145309	0.052642	0.049375	0.107748	0.253069	0.084335	0.073049	0.097132
comp4876_c0_seq1	Q6CBH3.1	0.048373	0.155994	0.16528	0.203664	0.214657	0.336127	0.322475	0.139127
comp4882_c0_seq1	Q9SVM8.1	0.192619	0.277741	0.266582	0.82785	0.395011	0.700169	0.281478	0.516112
comp4883_c0_seq1	Q5BD77.1	0.022212	0.0131	0.051121	0.040863	0.015974	0.049234	0.027053	0.0202
comp489_c0_seq1	A6U848.1	0	0	0.005861	0.014657	0.003471	0	0	0
comp4907_c0_seq1	O74493.1	0	0.033156	0.020206	0.049121	0	0.08641	0.027204	0.021533
comp4955_c0_seq1	Q1K9C4.1	0.038101	0.031538	0.018731	0.055382	0.030905	0.030207	0.027345	0.026933
comp4959_c0_seq1	P04551.1	0.012623	0.003558	0.003907	0.03423	0.028286	0.007515	0	0.014297
comp4960_c0_seq1	P04551.1	0.021925	0.017223	0.005861	0.055949	0.030684	0.014918	0.007638	0.006733
comp4988_c0_seq1	O00061.1	0.018319	0.003558	0.002729	0.017069	0.011549	0	0	0.009445
comp502_c0_seq1	A6ZY34.1	0.008606	0.003881	0.011776	0	0.004619	0.010698	0.012386	0.006733
comp505_c0_seq1	Q9URZ5.2	0.359316	0.142349	0.28152	0.427425	0.400515	0.147922	0.332417	0.245815
comp5053_c0_seq1	Q5UQG7.1	0.00541	0.084341	0.07147	0.061203	0.004619	0.046679	0.040792	0.035678
comp5075_c0_seq1	Q9PU58.2	0.046761	0.086284	0.047368	0.130792	0.093144	0.068251	0.084483	0.070243
comp508_c0_seq1	P22987.3	0.004999	0.012777	0.013459	0.042754	0.020492	0.022867	0.019678	0.025623
comp512_c0_seq1	P49742.1	0.009016	0.124662	0.136717	0.291044	0.071478	0.314512	0.155031	0.151283

comp515_c0_seq1	P40915.1	0.023032	0.024663	0.030639	0.083142	0.042909	0.02867	0.022549	0.056184
comp5153_c0_seq1	P39992.1	0.055939	0.060566	0.014637	0.130025	0.046689	0.084207	0.015032	0.064098
comp5163_c0_seq1	P38230.1	0.138765	0.062345	0.135809	0.194519	0.151804	0.118206	0.152738	0.179417
comp5165_c0_seq1	P97390.1	0	0.009219	0.011722	0.025685	0.003471	0.010569	0.012367	0.003017
comp5167_c0_seq1	Q9LUQ6.1	0.097824	0.280428	0.165536	0.198001	0.213212	0.125799	0.120614	0.174477
comp5170_c0_seq1	O13830.1	0.021639	0.003881	0	0.009741	0.003471	0	0	0
comp5225_c0_seq1	P79066.1	0.003607	0.022319	0.172121	0.043229	0.004619	0.257334	0.098102	0.111997
comp5260_c0_seq1	P26404.1	0.015819	0.107144	0.064527	0.048575	0.019621	0.076646	0.096984	0.028267
comp5268_c0_seq1	Q4WVS2.2	0.021639	0.007493	0.003907	0.069272	0.008943	0.020629	0.014545	0.049407
comp531_c0_seq1	P38093.1	0	0.003558	0.00241	0	0	0.010698	0.004393	0
comp5315_c0_seq1	Q99145.1	0.211429	0.034935	0.052987	0.092187	0.390717	0.070085	0.150542	0.065085
comp534_c0_seq1	Q10499.2	0.063797	0.149597	0.185206	0.149344	0.170228	0.354296	0.270732	0.294758
comp5349_c0_seq1	Q10294.1	0.007213	0.003558	0.005458	0.023273	0.012136	0	0.004393	0
comp5354_c0_seq1	Q4P3U9.1	0.003607	0.003558	0.005861	0.018403	0.013297	0.002814	0.005837	0.004722
comp5387_c0_seq1	B4U524.1	0.019015	0	0	0.029047	0.027125	0	0.003153	0
comp539_c0_seq1	P33723.1	0.03471	0.108975	0.075426	0.024443	0.005207	0.091775	0.065436	0.052139
comp54_c0_seq1	Q6V3W9.1	0.038727	0.01682	0.025372	0.059649	0.03473	0.023969	0.02085	0.021358
comp5409_c0_seq1	Q12559.2	0.00541	0.009381	0.008001	0.035517	0.010678	0	0.012245	0.008089
comp5434_c0_seq1	O13744.2	0.003607	0.010998	0.012205	0.017023	0.010767	0	0.008727	0.003367
comp5499_c0_seq1	Q8S403.1	0	0	0.003907	0.012199	0	0	0.002909	0.00505
comp5549_c0_seq1	Q04311.1	0.003607	0.020512	0.002729	0.041421	0.014326	0.014743	0.01494	0
comp556_c0_seq1	P78605.1	0.077006	0.033612	0.011319	0.060298	0.128827	0.009162	0.0137	0.023567
comp5573_c0_seq1	Q6BZX4.1	0.009016	0.014556	0.006823	0.017023	0.004619	0	0.002909	0.017861
comp5633_c0_seq1	Q6P158.2	0.007213	0	0.00241	0.009741	0.020403	0	0.003153	0.004722
comp5686_c0_seq1	P87061.1	0.103873	0.033962	0.043323	0.081297	0.057764	0.031391	0.035624	0.067313
comp5701_c0_seq1	Q9HGN5.1	0.012623	0.026631	0.014213	0.035993	0.030905	0.01292	0.025526	0.017642
comp5704_c0_seq1	Q9C0Y6.1	0.007213	0.009757	0.156773	0.012153	0.013474	0	0.182298	0.004722
comp5754_c0_seq1	Q14204.5	0.146683	0.128979	0.083471	0.185591	0.093744	0.088509	0.112805	0.097438
comp5760_c0_seq1	Q12740.1	0.01082	0	0	0.012199	0.006942	0	0.007292	0.007084
comp578_c0_seq1	P13259.2	0.065993	0.060943	0.024411	0.050337	0.033126	0.040682	0.004393	0.008089

comp5840_c0_seq1	Q9P7V2.1	0.012499	0.069085	0.022049	0.033397	0.003471	0.047225	0.030367	0.006733
comp5849_c0_seq1	P0CP71.1	0.012909	0.027657	0.03617	0	0	0.032558	0.006062	0.008089
comp5853_c0_seq1	P41889.2	0	0	0.003907	0.031031	0.004619	0.002814	0.002928	0
comp586_c0_seq1	Q75ZP8.1	2.914369	0.247395	0.388174	0.890105	2.141565	0.391079	0.088578	0.30757
comp5878_c0_seq1	P0CO99.1	0.015109	0.01116	0.047656	0.025731	0.014415	0.007755	0.028303	0.010756
comp589_c0_seq1	Q9Y697.3	0.019426	0.020379	0.014505	0.05392	0.032919	0.035898	0.00899	0.024922
comp5891_c0_seq1	Q8K157.1	0.101307	0.045418	0.068318	0.205619	0.094981	0.059533	0.111453	0.126098
comp5914_c0_seq1	Q8T9S7.1	0.009999	0.030216	0.006636	0.101616	0.018491	0.03387	0.002909	0.029317
comp5926_c0_seq1	Q4WY82.2	0.623073	0.249411	0.096111	0.767584	0.668604	0.509532	0.259681	0.466349
comp5935_c0_seq1	Q91W86.3	0.023318	0.024825	0.034144	0.079001	0.016181	0.026337	0.037912	0.014297
comp5981_c0_seq1	Q9UUA1.1	0	0.020943	0.003907	0.012153	0	0	0	0.009575
comp5984_c0_seq1	Q9UW21.1	0.003607	0.016659	0	0.047487	0.012887	0.014743	0.017532	0.012133
comp5987_c0_seq1	Q9UUA1.1	0.017498	0.049353	0.007815	0.058306	0.015872	0.020804	0.016331	0.014625
comp5989_c0_seq1	P22550.1	0.113683	0.225444	0.137014	0.295128	0.185072	0.155013	0.255612	0.159565
comp5996_c0_seq1	Q10318.1	0.012213	0.043316	0.025957	0.078655	0.032331	0.048419	0.028527	0.041865
comp601_c0_seq1	P09437.2	0	0.015041	0.022001	0.054524	0.013871	0.035148	0.024212	0.071029
comp606_c0_seq1	Q9Y7K9.2	0.058987	0.067275	0.027273	0.19969	0.021445	0.115152	0.012143	0.08163
comp610_c0_seq1	O60094.2	0.050748	0.020782	0.017664	0.062965	0.051472	0.013641	0.032939	0.016659
comp6100_c0_seq1	P62792.2	0.101309	0.678899	0.209236	0.368138	0.239927	0.195765	0.215766	0.343311
comp6103_c0_seq1	P31382.2	0.028853	0.031915	0.028637	0.065644	0.085746	0.027023	0.070656	0.066198
comp6105_c0_seq1	Q05567.1	0.017622	0.106224	0.059627	0.057319	0.022562	0.062395	0.050954	0.042389
comp611_c0_seq1	P0CQ97.1	0.170989	0.483981	0.224982	0.401846	0.183536	0.291998	0.326668	0.297406
comp6132_c0_seq1	O14009.1	0	0.003935	0	0.009741	0	0	0	0.012789
comp6145_c0_seq1	P25711.2	0.056118	0.01116	0.007815	0.004916	0.011562	0.038331	0.012123	0.018166
comp6171_c0_seq1	B0CPQ8.1	0.012213	0	0.006636	0.014611	0.011562	0	0	0.016528
comp6203_c0_seq1	O59748.1	0	0	0	0	0.003648	0.009051	0	0.003367
comp6207_c0_seq1	B2W3C7.1	0	0.003558	0.034354	0.017928	0	0.091446	0.012021	0.090418
comp624_c0_seq1	P0CQ77.1	0.029014	0.107682	0.044533	0.058407	0.021344	0.083715	0.023779	0.04709
comp6241_c0_seq1	P55251.1	0.076061	0.100674	0.066954	0.170413	0.082668	0.112368	0.097988	0.102312
comp6311_c0_seq1	Q09752.2	0.00541	0.019487	0	0.020431	0.003471	0.007884	0.006305	0.012417

comp6320_c0_seq1	C3K630.1	0.004999	0.052885	0.00859	0.007237	0.005207	0.023442	0.025701	0
comp641_c0_seq1	Q9YHY6.1	0.026925	0.041832	0.007549	0.068877	0.035304	0.026162	0.008766	0.010909
comp6472_c0_seq1	A6QLU1.1	0.095637	0.021673	0.02232	0.062756	0.103425	0.02867	0.021021	0.024748
comp6484_c0_seq1	P20654.1	0.105524	0.249109	0.217781	0.129167	0.05969	0.189917	0.162745	0.077937
comp6551_c0_seq1	P87115.1	0.014426	0.010998	0.012365	0.052632	0.025212	0.016695	0.017737	0.018867
comp6590_c0_seq1	Q9JHW0.1	0.047184	0.0131	0.024592	0.042708	0.033202	0.020452	0.01205	0.015172
comp6597_c0_seq1	P07921.1	0	0	0.002729	0.014657	0	0	0.004729	0.003367
comp660_c0_seq1	P39744.2	0.043906	0.016497	0.009955	0.063303	0.061545	0.02183	0.012274	0.011433
comp6615_c0_seq1	P09794.1	0.004999	0.035258	0.021709	0.009741	0.003648	0.061311	0.051768	0.0155
comp6644_c0_seq1	P79089.1	0.161932	0.228925	0.177405	0.43935	0.230095	0.21786	0.200526	0.315263
comp6653_c0_seq1	Q0IID9.1	0.007213	0.005902	0.009769	0.020386	0.006942	0.005757	0.002909	0.008067
comp6671_c0_seq1	P49008.2	0.009999	0	0.00859	0.026114	0	0.009051	0.015061	0.003367
comp6675_c0_seq1	P49008.2	0.009999	0	0.00859	0.026114	0	0.009051	0.015061	0.003367
comp67_c0_seq1	P0CG77.1	0.525867	1.498098	1.463127	0.323992	0.994409	2.396638	1.738704	0.37667
comp6718_c0_seq1	Q47944.1	0.009016	0.04453	0.041166	0.031634	0.012401	0.072278	0.044463	0.029272
comp677_c0_seq1	Q9C0Y6.1	0.246144	0.141211	0.157256	0.335151	0.782498	0.143424	0.160341	0.20397
comp678_c0_seq1	P28737.2	0.03616	0.028222	0.037976	0.06314	0.017122	0.02421	0.031446	0.016308
comp6795_c0_seq1	Q8R5L3.1	0.018605	0.018599	0.021252	0.038696	0.009239	0.010522	0.036175	0.03957
comp680_c0_seq1	Q5I0K3.2	0	0	0.003907	0.014565	0	0	0.002928	0.012133
comp6811_c0_seq1	Q10298.1	0	0	0.00241	0.014611	0.004619	0	0	0.00505
comp6839_c0_seq1	P34227.1	0.021105	0.066791	0.074693	0.03051	0.003471	0.080373	0.037527	0.083905
comp6890_c0_seq1	P84193.2	0.550761	0.038923	0.029852	0.267654	0.386253	0.032558	0.019454	0.028311
comp6916_c0_seq1	O14088.1	0.014426	0.022319	0.01932	0.032968	0.042732	0.019573	0.02755	0.016856
comp695_c0_seq1	Q9C0V4.1	0.027211	0.015094	0.008187	0.058178	0.017709	0.005627	0.043486	0.042084
comp6966_c0_seq1	Q1K9C2.1	0.058974	0.038273	0.01073	0.055474	0.095511	0.023108	0.02573	0.040094
comp6967_c0_seq1	P50276.1	0	0	0	0.02034	0	0	0	0
comp6993_c0_seq1	Q9P7V2.1	0.030942	0.024475	0.025368	0.057886	0.037494	0.019397	0.046595	0.02407
comp700_c0_seq1	Q1JQB2.1	0.01623	0.018438	0.011319	0.054907	0.003648	0.033261	0.02103	0.040925
comp7004_c0_seq1	Q8IYS1.2	0	0.02426	0.020871	0.018494	0	0.042394	0.056055	0.003367
comp7007_c0_seq1	Q8IYS1.2	0	0.02426	0.020871	0.018494	0	0.042394	0.056055	0.003367

comp7072_c0_seq1	P31434.2	0.165702	0.029274	0.036515	0.046929	0.120471	0.081957	0.012021	0.050501
comp712_c0_seq1	O74430.1	0.00541	0.003558	0	0.009741	0.006929	0	0.01799	0.004722
comp7145_c0_seq1	P20261.3	0	0.003881	0.002729	0.012153	0	0	0	0.003367
comp715_c0_seq1	Q4P9K9.1	0.087141	0.037629	0.019777	0.055857	0.035494	0.023618	0.02574	0.039001
comp7161_c0_seq1	P23262.4	0.008606	0.019003	0.011319	0	0	0.018582	0.052791	0.028662
comp718_c0_seq1	Q66JG3.1	0.026639	0.01682	0.020499	0.066027	0.049674	0.025616	0.049446	0.035722
comp726_c0_seq1	P33277.1	0.038851	0.040134	0.016374	0.093787	0.041981	0.034778	0.050695	0.063485
comp7335_c0_seq1	P49426.1	0.008606	0.043021	0.070458	0	0.007296	0.044441	0.063754	0.038542
comp735_c0_seq1	O74462.1	0.01082	0.005337	0.016591	0.07913	0.033391	0.024515	0.01798	0.025272
comp7356_c0_seq1	Q5FPZ4.1	0	0.003558	0	0.028189	0.007119	0.005627	0.002909	0.00505
comp7373_c0_seq1	Q9USS7.1	0.027963	0.018841	0.004094	0.048483	0.005472	0.013336	0.019547	0.0155
comp744_c0_seq1	O14333.4	0.020122	0.018599	0.019001	0.03633	0.038686	0.015288	0.022363	0.025075
comp7470_c0_seq1	Q43139.2	0.261491	0.261638	0.077747	0.123475	0.159753	0.15174	0.291031	0.14457
comp7473_c0_seq1	Q6DBY2.1	0.009016	0.024637	0.016777	0.05922	0.016181	0.020229	0.010201	0.035044
comp749_c0_seq1	P0CP73.1	0.009016	0.009381	0.009366	0.019527	0.012136	0	0	0.00975
comp753_c0_seq1	Q00078.1	0.102414	0.116768	0.080188	0.146977	0.161949	0.10837	0.06401	0.156091
comp7536_c0_seq1	Q9C107.1	0.012213	0.014718	0.019866	0.045121	0.017696	0.014789	0.018204	0.0209
comp764_c0_seq1	Q02899.3	0.082429	0.097115	0.218941	0.206422	0.149159	0.10926	0.275221	0.2605
comp769_c0_seq1	B2WBQ6.1	0.133055	0.149812	0.119834	0.200748	0.140482	0.079441	0.177895	0.146672
comp77_c0_seq1	P28040.1	0.041842	0.013395	0.007523	0.05232	0.012502	0.040905	0.013456	0.013117
comp771_c0_seq1	P33202.1	0.031228	0.052911	0.050257	0.090204	0.0278	0.05026	0.048308	0.044094
comp7710_c0_seq1	P38754.1	0.365759	0.317577	0.267664	0.492116	0.37214	0.288239	0.313096	0.328108
comp774_c0_seq1	O14035.1	0.003607	0	0	0.017069	0.007296	0	0	0
comp775_c0_seq1	Q7L5Y9.1	0	0.003558	0.003907	0.009741	0.003648	0	0.003153	0.00505
comp780_c0_seq1	P36594.1	0.068207	0.061889	0.0395	0.132995	0.056175	0.06964	0.052525	0.081347
comp781_c0_seq1	P36594.1	0	0.007763	0.002729	0.014611	0.003471	0	0.007302	0
comp7877_c0_seq1	O74314.1	0	0	0.002729	0.009741	0	0	0.003153	0.008089
comp7879_c0_seq1	P38861.3	0.009999	0.003558	0.005861	0.037838	0.010577	0.01615	0.005857	0.025272
comp7889_c0_seq1	P38675.3	0	0	0.002729	0.017023	0	0	0.004364	0.004722
comp7935_c0_seq1	P29064.1	0.03691	0.045363	0.020371	0.046117	0.019652	0.064523	0.011899	0.01045

comp7944_c0_seq1	O94255.1	0	0	0	0.015515	0.003471	0	0.007657	0
comp797_c0_seq1	O13775.1	0.552715	0.188067	0.116968	0.460716	0.405885	0.18396	0.244107	0.252724
comp7981_c0_seq1	Q08DI8.1	0.015409	0.026496	0.010438	0.04767	0.017608	0.008635	0.016385	0.040159
comp8044_c0_seq1	Q5HZM6.1	0.004999	0.003881	0.00241	0.018403	0	0.005757	0	0.013117
comp805_c0_seq1	Q9UUF2.1	0.033728	0.063963	0.035508	0.118997	0.058781	0.052593	0.045175	0.060359
comp812_c0_seq1	P38754.1	0	0.057682	0.027717	0	0	0.037628	0	0.00505
comp8185_c0_seq1	Q9P7T8.1	0.012213	0.009219	0.015816	0.035042	0.069918	0.004414	0.01311	0.016178
comp820_c0_seq1	Q63569.1	0.19421	0.360751	0.21663	0.315187	0.165128	0.228946	0.260051	0.201104
comp8206_c0_seq1	O94505.1	0.274459	0.084095	0.23071	0.07078	0.460047	0.042997	0.275434	0.044817
comp825_c0_seq1	O94153.1	0.018319	0.013638	0.008001	0	0.022139	0.03774	0.014789	0.0101
comp8278_c0_seq1	Q9HFF5.1	0.029139	0.033747	0.022049	0.070852	0.01059	0.027439	0.01022	0.022233
comp829_c0_seq1	Q5KPF3.1	0.007213	0.049433	0.046779	0.018403	0.007119	0.033724	0.024003	0.01515
comp8303_c0_seq1	Q642H7.1	0.027621	0.026201	0.015227	0.015607	0.010767	0.042838	0.01188	0.00505
comp8304_c0_seq1	P41891.2	0.019015	0.057249	0.019506	0.052066	0.052439	0.031438	0.017868	0.044356
comp8323_c0_seq1	Q05871.1	0.017622	0.031861	0.020499	0.070815	0.019829	0.033002	0.028556	0.011806
comp8361_c0_seq1	Q8TB22.3	0.010409	0.005822	0.009955	0.015515	0.008943	0.003294	0.024052	0.014823
comp8362_c0_seq1	Q9C0Y6.1	0	0.045122	0.047182	0.061019	0	0.042681	0.035698	0.01515
comp837_c0_seq1	P34809.3	0.088436	0.039758	0.069582	0.122706	0.073358	0.043607	0.085435	0.06012
comp839_c0_seq1	Q3SZH7.3	0.084067	0.163429	0.13344	0.260583	0.086815	0.124261	0.13349	0.220999
comp8484_c0_seq1	O94413.1	0.046228	0.018761	0.019506	0.060124	0.10066	0.020804	0.027102	0.024594
comp854_c0_seq1	Q10342.2	0.039138	0.00744	0.004094	0.043229	0.03117	0.018102	0.023949	0.012614
comp8547_c0_seq1	O36021.1	0.003607	0.011724	0	0.027622	0.030009	0.002814	0.007292	0.009772
comp857_c0_seq1	P05373.2	0.066075	0.083369	0.146242	0.021673	0.026564	0.07994	0.081961	0.021228
comp858_c0_seq1	O13651.1	0.300103	0.251002	0.196562	0.429145	0.177534	0.178982	0.215208	0.219534
comp8594_c0_seq1	Q03392.1	0.014712	0.074876	0.023684	0.079971	0.003471	0.073462	0	0.018975
comp86_c0_seq1	Q9USQ4.1	0.014426	0.038435	0.030618	0.090945	0.027624	0.028365	0.019912	0.062829
comp860_c0_seq1	Q68FU3.3	0.021639	0.039408	0.025181	0.048841	0.031859	0.022322	0.007321	0.017817
comp866_c0_seq1	O94590.1	0.101472	0.065474	0.051489	0.152194	0.076596	0.06903	0.085275	0.088387
comp8761_c0_seq1	O14088.1	0.018033	0	0	0.02289	0.006929	0	0.002928	0.00505
comp889_c0_seq1	Q03002.1	0.022622	0.043504	0.028983	0.070943	0.031965	0.032639	0.032686	0.047309

comp8921_c0_seq1	Q9F131.1	0.01082	0.010675	0.004094	0	0	0.062783	0.018092	0.008089
comp8922_c0_seq1	Q9F131.1	0.01082	0.010675	0.004094	0	0	0.062783	0.018092	0.008089
comp893_c0_seq1	Q9Y7M5.1	0	0	0	0.012199	0.020593	0	0.002928	0.007084
comp8983_c0_seq1	P49374.1	0.00541	0	0.003907	0.017928	0.007119	0	0.002909	0.004722
comp9008_c0_seq1	Q03441.1	0.011106	0.026093	0.046349	0.080309	0.01271	0.00918	0.063825	0.026605
comp9089_c0_seq1	Q9V9A7.1	0.019836	0.015659	0.014637	0.039913	0.01059	0.013465	0.01673	0.016133
comp93_c0_seq1	Q12200.1	0.017622	0.010998	0.020955	0.048391	0.013562	0.020804	0.044419	0.016505
comp9385_c0_seq1	P39864.1	0	0	0	0.009741	0	0	0	0
comp94_c0_seq1	O94562.1	0.052457	0.011698	0.012981	0.048575	0.074061	0.030207	0.002928	0.01915
comp95_c0_seq1	P0CP31.1	0.034138	0.033855	0.019909	0.078655	0.034553	0.039017	0.020928	0.045931
comp954_c0_seq1	P63009.2	0.044833	0.150433	0.058506	0.117735	0.072709	0.091564	0.078867	0.102204
comp9561_c0_seq1	Q9P7U2.1	0	0.070323	0.003907	0.210953	0	0.034861	0.01081	0.042061
comp9575_c0_seq1	Q9P7U2.1	0	0.070323	0.003907	0.210953	0	0.034861	0.01081	0.042061
comp963_c0_seq1	P42892.2	0.046104	0.043989	0.034684	0.12818	0.031846	0.075812	0.04066	0.073476
comp974_c0_seq1	P38226.1	0.012499	0	0.005458	0.023227	0.012591	0.009051	0.014959	0.008416
comp976_c0_seq1	P23231.2	0.038317	0.162639	0.089252	0.146894	0.032919	0.087595	0.10401	0.100104
comp9796_c0_seq1	Q9Y8G7.1	0	0.025716	0.007709	0	0	0.026402	0.012143	0.018189
comp9801_c0_seq1	Q9Y8G7.1	0	0.01752	0.005458	0	0	0.038923	0.019669	0
comp982_c0_seq1	O42781.1	0	0.003881	0.005458	0.053828	0.016181	0.016677	0.018112	0.008416
comp983_c0_seq1	P0CP67.1	0.023318	0.011321	0.017664	0.068823	0.010413	0.028911	0	0.019872
comp9839_c0_seq1	Q4WCR3.1	0.003607	0.084825	0.020238	0.035272	0.005207	0.04222	0.021895	0.01915
comp9840_c0_seq1	Q8CGC4.3	0.021229	0.014718	0.010916	0.0428	0.050394	0.031373	0.027326	0.019217
comp9843_c0_seq1	Q9QYZ3.1	0	0.005902	0.005861	0.009741	0	0	0	0
comp9865_c0_seq1	Q8HXM1.1	0.00541	0.007493	0.002729	0.039263	0.008943	0.007755	0.017849	0.019195
comp9885_c0_seq1	P0CO83.1	0	0	0.010916	0	0.007119	0.018342	0.010425	0.007084
comp9886_c0_seq1	Q9R111.1	0	0.051912	0.010225	0	0.008091	0.014807	0.007516	0
comp9900_c0_seq1	Q12019.1	0.024425	0.029974	0.015737	0.037976	0.031846	0.015112	0.017878	0.02525
comp9910_c0_seq1	O94751.1	0.003607	0.0169	0.007868	0.028143	0.015784	0.009977	0.007657	0.013139
comp9914_c0_seq1	Q8YVS7.1	0.031105	0.047063	0.054966	0.090634	0.024051	0.038618	0.048462	0.049451
comp992_c0_seq1	P70627.1	0.021925	0.015094	0.027724	0.070897	0.015872	0.02955	0.027112	0.03065

* These labels represented the same conditions described in Figure 2 and Figure 3 legend.

Supplementary Table 4. The functional enrichment of differentially expressed proteins between dye/lignin combined treatment and dye or lignin as the single substrate.

GO Term	Gene Count	%	P-Value	Benjamini
Lignin+Dye v.s. Dye				
catalytic activity	87	69.0	2.8E-2	1.0E0
protein transporter activity	5	4.0	2.9E-2	1.0E0
oxidoreductase activity	21	16.7	3.3E-2	9.9E-1
coenzyme binding	9	7.1	4.8E-2	1.0E0
Lignin+Dye v.s. Lignin				
nucleotidyltransferase activity	7	9.6	5.7E-3	8.0E-1
oxidoreductase activity	16	21.9	7.4E-3	6.5E-1
oxidoreductase activity, acting on CH-OH group of donors	7	9.6	1.2E-2	6.9E-1
structural constituent of ribosome	7	9.6	2.6E-2	8.5E-1
oxidoreductase activity, acting on the CH-OH group of donors, NAD or NADP as acceptor	6	8.2	2.8E-2	8.0E-1
coenzyme binding	7	9.6	3.0E-2	7.6E-1
structural molecule activity	8	11.0	3.5E-2	7.6E-1
cofactor binding	9	12.3	3.6E-2	7.3E-1
NAD binding	4	5.5	4.0E-2	7.3E-1
sulfate adenylyltransferase activity	2	2.7	4.8E-2	7.5E-1

Supplementary Table 5. Differentially expressed oxidation reduction related proteins during different stages of DR5B decolorization by *I. lacteus* CD2

Protein ID	Protein name	Normanized expression abundance					
		Dye_day1*	Dye_day3*	Dye_day5*	Lignin+Dye _day1*	Lignin+Dye _day3*	Lignin+Dye _day5*
comp1026_c0_seq1	Uncharacterized oxidoreductase YusZ	0.012909	0.007117	0.027507	0.015784	0.019878	0.026775
comp10633_c0_seq1	Ceramide very long chain fatty acid hydroxylase SCS7	0.003607	0	0	0.005207	0.007163	0.004393
comp10841_c0_seq1	D-arabinitol dehydrogenase 1	0.017909	0	0.020096	0.008267	0.035482	0.016048
comp10867_c0_seq1	Uncharacterized oxidoreductase C736.13	0.079642	0.00744	0.014637	0.039733	0	0.002909
comp10887_c0_seq1	Coproporphyrinogen-III oxidase, mitochondrial	0.069421	0.127544	0.051706	0.03473	0.072278	0.148963
comp10893_c0_seq1	Uncharacterized oxidoreductase C977.08/C1348.09	1.433883	2.925966	1.41707	1.896021	3.226742	2.147412
comp10917_c0_seq1	Putative aryl-alcohol dehydrogenase C977.14c	0.490907	0.10744	0.062351	0.074679	0.070074	0.210105
comp10918_c0_seq1	Putative aryl-alcohol dehydrogenase C977.14c	3.040926	2.348992	1.295823	2.612027	1.29779	1.390805
comp10942_c0_seq1	Putative sterigmatocystin biosynthesis peroxidase stcC	0	0.030485	0.002729	0	0.042616	0
comp1098_c0_seq1	D-arabinose 1-dehydrogenase	0.018033	0.01116	0.002729	0.045717	0	0.002928
comp11028_c0_seq1	Putative glycine dehydrogenase [decarboxylating], mitochondrial	0.039424	0.0169	0.002729	0.021256	0	0.004364
comp11072_c0_seq1	Delta(12) fatty acid desaturase	0.062456	0.079515	0.056123	0.034067	0.090686	0.106612
comp11085_c0_seq1	Cytochrome P450 67 (Fragment)	0	0.009381	0	0.003471	0.005886	0.002928
comp11088_c0_seq1	Uncharacterized oxidoreductase C663.06c	0.296573	0.227098	0.719176	0.928044	0.302728	0.905529
comp11116_c0_seq1	Putative peroxiredoxin (Fragment)	0.120842	0.642758	0.531603	0.140331	0.905579	0.52341
comp11136_c0_seq1	Elongation factor Tu, mitochondrial	0.372471	0.607441	0.273127	0.326387	0.372943	0.24681

comp11140_c0_seq1	Probable NAD(P)H-dependent D-xylose reductase xyl1	0.046692	0.065635	0.005458	0.051971	0.039803	0.009122
comp11144_c0_seq1	Psi-producing oxygenase A	0.091936	0.183429	0.073892	0.167792	0.141501	0.101649
comp11147_c0_seq1	Probable formate dehydrogenase	0.058153	0.009381	0.006636	0.041495	0.058015	0.045155
comp11166_c0_seq1	NADPH--cytochrome P450 reductase	0.072455	0.23901	0.132739	0.096496	0.191199	0.225554
comp11175_c0_seq1	Delta-1-pyrroline-5-carboxylate dehydrogenase	0.418734	0.44912	0.539283	0.098598	0.820254	0.569768
comp11253_c0_seq1	Aldehyde dehydrogenase	0.230754	0.130997	0.238662	0.248888	0.168842	0.344868
comp11254_c0_seq1	Putative peroxiredoxin pmp20	0	0	0.013862	0	0.02234	0.031802
comp11264_c0_seq1	Probable peptide methionine sulfoxide reductase	0.102713	0.035258	0.018253	0.0407	0.025746	0.020787
comp11281_c0_seq1	Dihydrolipoyl dehydrogenase, mitochondrial	0.009016	0.091486	0.123704	0.031554	0.229202	0.131343
comp11284_c0_seq1	Probable succinate dehydrogenase [ubiquinone] flavoprotein subunit, mitochondrial	0.416537	0.331948	0.393615	0.577574	0.388632	0.726652
comp11340_c0_seq1	Putative aryl-alcohol dehydrogenase C977.14c	0.044139	0.047762	0.013862	0.117158	0.019509	0.009093
comp11343_c0_seq1	Ribonucleoside-diphosphate reductase large chain	0.094256	0.217285	0.181255	0.143745	0.200524	0.233631
comp11355_c0_seq1	Superoxide dismutase [Mn], mitochondrial	0.123316	0.050299	0.08643	0.076786	0.082155	0.085474
comp11413_c0_seq1	NADH-ubiquinone oxidoreductase 20.8 kDa subunit	0.036066	0.054905	0.016458	0.055923	0.031549	0.023808
comp11414_c0_seq1	Prohibitin-2	0.018033	0.037897	0.024003	0.064195	0.021331	0.040324
comp11433_c0_seq1	Inosine-5'-monophosphate dehydrogenase	0.030656	0.00744	0.014775	0.056413	0.028929	0.037761
comp11441_c0_seq1	Protein GCY	0.127058	0.102646	0.107899	0.156367	0.14945	0.153431
comp11477_c0_seq1	Putative reductase 1	0.057904	0.056201	0.087586	0.02474	0.18517	0.11061
comp11486_c0_seq1	Versatile peroxidase VPL2	0	0.003881	0	0	0.02992	0.004729
comp11491_c0_seq1	Succinate dehydrogenase [ubiquinone] cytochrome b small subunit, mitochondrial	0.134826	0.016982	0.031849	0.059266	0.033261	0.025535

comp11497_c0_seq1	UDP-glucose 6-dehydrogenase 3	0.189374	0.248197	0.191042	0.142283	0.137105	0.149694
comp11505_c0_seq1	NADH-cytochrome b5 reductase 2	0.069645	0.137171	0.065754	0.125223	0.111276	0.09205
comp11519_c0_seq1	Sphingolipid C4-hydroxylase SUR2	0	0	0	0.004619	0	0.007545
comp11564_c0_seq1	NAD(P) transhydrogenase, mitochondrial	0.026694	0.042993	0.024083	0.05341	0.036268	0.043608
comp11597_c0_seq1	Uncharacterized oxidoreductase C513.06c	0.012499	0.013342	0.006823	0.041968	0.019878	0.01676
comp11612_c0_seq1	Diacetyl reductase [(S)-acetoin forming]	0.101798	0.094507	0.235722	0.253776	0.023747	0.45998
comp11723_c0_seq1	Cytochrome b2, mitochondrial	0.049423	0.043424	0.036692	0.138475	0.039803	0.056981
comp11732_c0_seq1	Alcohol oxidase	0.112989	0.083182	0.020079	0.230604	0.144416	0.013363
comp11734_c0_seq1	72 kDa inositol polyphosphate 5-phosphatase	0.009016	0.003881	0.003907	0.011562	0	0.002909
comp11737_c0_seq1	Probable NAD-dependent malic enzyme 3	0.012623	0.051079	0.045012	0.035405	0.0422	0.055498
comp11773_c0_seq1	Short/branched chain specific acyl-CoA dehydrogenase, mitochondrial	0	0	0.004094	0.003648	0.004414	0
comp1200_c0_seq1	Aflatoxin B1 aldehyde reductase member 3	0.050654	0.052858	0.075358	0.064504	0.031854	0.091
comp128_c0_seq1	Pyridoxal reductase	0.061897	0	0.016591	0.044454	0	0.020899
comp1309_c0_seq1	Putative glutamate synthase [NADPH]	0.082443	0.117847	0.065683	0.175587	0.081347	0.070277
comp1338_c0_seq1	Deoxyhypusine hydroxylase	0.015409	0.003935	0.003907	0.004619	0	0
comp1382_c0_seq1	Putative aryl-alcohol dehydrogenase C977.14c	0.027497	0.089004	0.041454	0	0.005627	0.040231
comp1405_c0_seq1	Peroxisomal catalase	3.399095	3.702818	5.67768	3.873486	6.096779	3.950232
comp1425_c0_seq1	Glucose-6-phosphate 1-dehydrogenase	0.488947	0.436117	0.316103	0.419743	0.401456	0.424672
comp1446_c0_seq1	Linoleate 10R-lipoxygenase	0.039603	0.068414	0.031663	0.072564	0.067353	0.031212
comp1449_c0_seq1	Linoleate 10R-lipoxygenase	0.039603	0.068414	0.031663	0.072564	0.067353	0.031212
comp1499_c0_seq1	Aryl-alcohol dehydrogenase [NADP(+)]	0.114188	0.509955	1.087463	0.34533	0.205969	1.08388

comp1501_c0_seq1	Probable homoserine dehydrogenase	0.062852	0.022911	0.014319	0.057911	0.065114	0.022081
comp1519_c0_seq1	Homoisocitrate dehydrogenase	0.016516	0.015256	0.036346	0.048601	0.020675	0.04662
comp1644_c0_seq1	Protein MSP1	0.003607	0.027172	0.035508	0.0104	0.022756	0.037337
comp1770_c0_seq1	6-hydroxy-D-nicotine oxidase	0	0.020055	0.004094	0	0.025699	0.066788
comp1796_c0_seq1	Pyridoxal reductase	0.063563	0.028706	0.095188	0.086038	0.010698	0.133358
comp1799_c0_seq1	Alcohol oxidase	0	0	0.003615	0.009826	0.016871	0.019313
comp1989_c0_seq1	Putative dioxygenase C576.01c	0.155404	0.037414	0.056379	0.065325	0.073814	0.072908
comp20_c0_seq1	Probable 2-oxoglutarate dehydrogenase E1 component DHKTD1, mitochondrial	0.033372	0.022561	0.017234	0.028008	0.018582	0.016068
comp2072_c0_seq1	Succinate dehydrogenase [ubiquinone] iron-sulfur subunit, mitochondrial	0.055081	0.096416	0.088145	0.0769	0.065883	0.050019
comp2076_c0_seq1	NAD/NADP-dependent indole-3-acetaldehyde reductase	0.059384	0.057981	0.079612	0.129988	0.120826	0.195168
comp2181_c0_seq1	Uncharacterized oxidoreductase C663.06c	0.027049	0.033048	0.087236	0.095511	0.034556	0.138626
comp2188_c0_seq1	Hydroxyacid-oxoacid transhydrogenase, mitochondrial	0.067169	0.04488	0.034844	0.075032	0.064523	0.018909
comp2221_c0_seq1	Uncharacterized oxidoreductase C30D10.05c	0.038606	0.03744	0.023095	0.083843	0.031068	0.022466
comp2223_c0_seq1	Pyruvate dehydrogenase E1 component subunit beta, mitochondrial	0.047047	0.069597	0.076803	0.055998	0.057424	0.075807
comp223_c0_seq1	Aryl-alcohol dehydrogenase [NADP(+)]	0.070079	0.157534	0.566314	0.222659	0.105874	0.771682
comp2308_c0_seq1	GDP-L-fucose synthase	0.020819	0.04523	0.042331	0.040422	0.041496	0.077242
comp232_c0_seq1	Quinone oxidoreductase PIG3	0.037908	0.018976	0.014824	0.034641	0.0252	0.062258
comp2410_c0_seq1	Alcohol oxidase	0.053564	0.020943	0.005861	0.0996	0.018277	0.015203
comp2500_c0_seq1	Probable quinone oxidoreductase	0.064068	0.031753	0.038135	0.065559	0.069547	0.059842
comp2547_c0_seq1	Probable sulfite reductase [NADPH] flavoprotein component	0.093862	0.03744	0.034179	0.092331	0.027153	0.015427

comp2681_c0_seq1	Delta(12) fatty acid desaturase	0	0.003881	0.005139	0	0.006108	0
comp269_c0_seq1	Alpha-aminoadipic semialdehyde synthase, mitochondrial	0.023729	0.011321	0.021757	0.031744	0.003294	0.049538
comp2691_c0_seq1	Cytochrome c oxidase subunit 6, mitochondrial	0.101633	0.093264	0.251653	0.089257	0.199313	0.400976
comp2742_c0_seq1	Calcium-binding mitochondrial carrier protein Aralar1	0.143326	0.071994	0.04207	0.078225	0.040507	0.048561
comp2756_c0_seq1	NADH-ubiquinone oxidoreductase 49 kDa subunit, mitochondrial	0.088207	0.056793	0.056521	0.096994	0.063467	0.087796
comp276_c0_seq1	NAD/NADP-dependent indole-3-acetaldehyde reductase	0.01082	0.020217	0.021009	0.036465	0.048742	0.113475
comp2778_c0_seq1	Zinc-type alcohol dehydrogenase-like protein C2E1P3.01	0.021229	0.061319	0.048015	0.026771	0.03673	0.043506
comp2798_c0_seq1	Aldo-keto reductase yakc [NADP(+)]	0.004999	0.00744	0.075438	0.01521	0.042101	0.121255
comp2800_c0_seq1	D-amino-acid oxidase	0.04594	0.020379	0.022001	0.053971	0.021045	0.019557
comp2849_c0_seq1	Ent-kaurene oxidase	0	0.016336	0.002729	0	0.02533	0.002909
comp297_c0_seq1	Putative aryl-alcohol dehydrogenase C977.14c	0.056884	0.007117	0.15513	0.039243	0.024626	0.250157
comp2977_c0_seq1	D-3-phosphoglycerate dehydrogenase	0.070079	0.065715	0.053464	0.109917	0.048308	0.08976
comp3053_c0_seq1	Putative aryl-alcohol dehydrogenase C977.14c	0.803275	0.505727	0.259963	0.459133	0.366994	0.268812
comp3095_c0_seq1	Salicylate hydroxylase	0.003607	0.071296	0.069466	0.003648	0.01377	0.110201
comp3277_c0_seq1	D-serine dehydratase	0.007213	0.019029	0.036489	0.010413	0.042376	0.033999
comp3318_c0_seq1	1,2-dihydroxy-3-keto-5-methylthiopentene dioxygenase 2	0.039916	0.080462	0.02791	0.026184	0.12337	0.048773
comp3328_c0_seq1	Probable NAD(P)H-dependent D-xylose reductase xyl1	0.066143	0.734609	0.829477	0.07206	0.913692	0.348511
comp3419_c0_seq1	PKHD-type hydroxylase TPA1	0.035941	0.116308	0.036081	0.022505	0.248002	0.03762
comp3428_c0_seq1	Peroxiredoxin C1773.02c	0.024425	0.021481	0.012365	0.039159	0.054176	0.012245
comp3491_c0_seq1	Hydroxyquinol 1,2-dioxygenase	0.016516	0.055175	0.140308	0.027624	0.125307	0.118146
comp3494_c0_seq1	Hydroxyquinol 1,2-dioxygenase	0.028728	0.050699	0.150104	0.037538	0.134598	0.099652

comp3525_c0_seq1	Bifunctional P-450:NADPH-P450 reductase	0	0.018599	0.00241	0.005472	0.018277	0.004364
comp3895_c0_seq1	Cytochrome P450 67 (Fragment)	0	0	0	0	0.002814	0
comp3951_c0_seq1	Uncharacterized oxidoreductase C736.13	0	0.00744	0	0	0.002943	0.002928
comp3984_c0_seq1	NADPH-dependent 1-acyldihydroxyacetone phosphate reductase	0.00541	0.003558	0.022346	0.019343	0.023026	0.040183
comp4017_c0_seq1	Cytochrome P450 67 (Fragment)	0.009999	0	0.003907	0.015974	0	0.010454
comp4180_c0_seq1	D-amino-acid oxidase	0.009016	0.016982	0.009233	0.019343	0.031549	0.014603
comp4194_c0_seq1	Aflatoxin B1 aldehyde reductase member 4	0	0.135447	0.075141	0	0.052529	0.191843
comp4214_c0_seq1	Formate dehydrogenase	0.074955	0.008896	0.056893	0.040908	0.085807	0.20575
comp4226_c0_seq1	NADPH-dependent methylglyoxal reductase GRE2	0.12621	0.137173	0.267318	0.088158	0.091516	0.379885
comp4367_c0_seq1	Aldehyde dehydrogenase	0.086222	0.037764	0.052751	0.049303	0.036361	0.057269
comp4411_c0_seq1	Lysine-specific demethylase 5A	0.026515	0.026496	0.017664	0.021079	0.027458	0.016272
comp4443_c0_seq1	Phenol 2-monooxygenase	0.007499	0.040864	0.04362	0.005472	0.003294	0.070335
comp4445_c0_seq1	Lanosterol 14-alpha demethylase	0.026925	0.036199	0.052587	0.023181	0.021636	0.067611
comp4485_c0_seq1	Choline dehydrogenase	0.026228	0.165964	0.442046	0.010413	0.456142	0.602321
comp4490_c0_seq1	Bifunctional P-450:NADPH-P450 reductase	0.044178	0.0355	0.009073	0.015872	0.024274	0.002928
comp468_c0_seq1	Pentachlorophenol 4-monooxygenase	0.012623	0.189058	0.138697	0.039261	0.104085	0.206782
comp4778_c0_seq1	Manganese peroxidase 3	0.007499	0.013315	0.019006	0	0.047874	0.062619
comp4780_c0_seq1	Manganese peroxidase 3	0.011106	0.011724	0.011165	0.003648	0.109019	0.077983
comp4869_c0_seq1	Uncharacterized oxidoreductase C26H5.09c	0.145309	0.052642	0.049375	0.253069	0.084335	0.073049
comp4988_c0_seq1	Cytochrome P450 67 (Fragment)	0.018319	0.003558	0.002729	0.011549	0	0
comp515_c0_seq1	NADH-ubiquinone oxidoreductase 24 kDa subunit, mitochondrial	0.023032	0.024663	0.030639	0.042909	0.02867	0.022549

comp5163_c0_seq1	Probable quinone oxidoreductase	0.138765	0.062345	0.135809	0.151804	0.118206	0.152738
comp534_c0_seq1	Apoptosis-inducing factor 1	0.063797	0.149597	0.185206	0.170228	0.354296	0.270732
comp54_c0_seq1	Dihydroorotate dehydrogenase (quinone), mitochondrial	0.038727	0.01682	0.025372	0.03473	0.023969	0.02085
comp5704_c0_seq1	Zinc-type alcohol dehydrogenase-like protein PB24D3.08c	0.007213	0.009757	0.156773	0.013474	0	0.182298
comp5760_c0_seq1	Pyrroline-5-carboxylate reductase	0.01082	0	0	0.006942	0	0.007292
comp586_c0_seq1	N/A	2.914369	0.247395	0.388174	2.141565	0.391079	0.088578
comp5926_c0_seq1	Linoleate 10R-lipoxygenase	0.623073	0.249411	0.096111	0.668604	0.509532	0.259681
comp601_c0_seq1	Cytochrome b2, mitochondrial	0	0.015041	0.022001	0.013871	0.035148	0.024212
comp6145_c0_seq1	NADH-ubiquinone oxidoreductase 21 kDa subunit, mitochondrial	0.056118	0.01116	0.007815	0.011562	0.038331	0.012123
comp6472_c0_seq1	Glycerol-3-phosphate dehydrogenase, mitochondrial	0.095637	0.021673	0.02232	0.103425	0.02867	0.021021
comp677_c0_seq1	Zinc-type alcohol dehydrogenase-like protein PB24D3.08c	0.246144	0.141211	0.157256	0.782498	0.143424	0.160341
comp678_c0_seq1	Protein MSP1	0.03616	0.028222	0.037976	0.017122	0.02421	0.031446
comp6839_c0_seq1	Mitochondrial peroxiredoxin PRX1	0.021105	0.066791	0.074693	0.003471	0.080373	0.037527
comp6916_c0_seq1	Uncharacterized oxidoreductase C2F3.05c	0.014426	0.022319	0.01932	0.042732	0.019573	0.02755
comp6966_c0_seq1	Sulfite reductase [NADPH] subunit beta	0.058974	0.038273	0.01073	0.095511	0.023108	0.02573
comp7161_c0_seq1	Salicylate hydroxylase	0.008606	0.019003	0.011319	0	0.018582	0.052791
comp8362_c0_seq1	2-alkenal reductase (NADP(+)-dependent)	0	0.045122	0.047182	0	0.042681	0.035698
comp860_c0_seq1	Electron transfer flavoprotein subunit beta	0.021639	0.039408	0.025181	0.031859	0.022322	0.007321
comp8761_c0_seq1	Uncharacterized oxidoreductase C2F3.05c	0.018033	0	0	0.006929	0	0.002928
comp9561_c0_seq1	Putative aryl-alcohol dehydrogenase C977.14c	0	0.070323	0.003907	0	0.034861	0.01081

comp9575_c0_seq1	Putative aryl-alcohol dehydrogenase C977.14c	0	0.070323	0.003907	0	0.034861	0.01081
comp9796_c0_seq1	Bifunctional P-450:NADPH-P450 reductase	0	0.025716	0.007709	0	0.026402	0.012143
comp9801_c0_seq1	Bifunctional P-450:NADPH-P450 reductase	0	0.01752	0.005458	0	0.038923	0.019669
comp9914_c0_seq1	N/A	0.031105	0.047063	0.054966	0.024051	0.038618	0.048462

* These labels represented the same conditions described in Figure 3 legend.

Supplementary Table 6. Functional enrichment analysis of each module from total protein co-expression network.

Module	Node Number	Enrichment Biological process				
		Term	Gene Count	Gene %	p-Value	FDR
Black	223	GO:0055085 transmembrane transport	11	6.043956	0.002746	3.761306
		GO:0051726 regulation of cell cycle	8	4.395604	0.036731	40.65402
Blue	387	GO:0010605 negative regulation of macromolecule metabolic process	23	6.990881	1.77E-04	0.284435
		GO:0044257 cellular protein catabolic process	26	7.902736	9.48E-04	1.513101
		GO:0030163 protein catabolic process	26	7.902736	0.001213	1.932149
		GO:0006397 mRNA processing	24	7.294833	0.001486	2.362625
		GO:0044265 cellular macromolecule catabolic process	33	10.0304	0.001919	3.042103
		GO:0016071 mRNA metabolic process	28	8.510638	0.001925	3.050497
		GO:0051172 negative regulation of nitrogen compound metabolic process	16	4.863222	0.002024	3.204974
		GO:0045934 negative regulation of nucleobase, nucleoside, nucleotide and nucleic acid metabolic process	16	4.863222	0.002024	3.204974
		GO:0016481 negative regulation of transcription	13	3.951368	0.002028	3.211358
		GO:0051603 proteolysis involved in cellular protein catabolic process	24	7.294833	0.002145	3.393786
		GO:0031327 negative regulation of cellular biosynthetic process	16	4.863222	0.002393	3.77891
		GO:0019941 modification-dependent protein catabolic process	23	6.990881	0.003022	4.749566
		GO:0009890 negative regulation of biosynthetic process	16	4.863222	0.003299	5.17505
		GO:0010558 negative regulation of macromolecule biosynthetic process	16	4.863222	0.003299	5.17505
		GO:0010629 negative regulation of gene expression	15	4.559271	0.00347	5.436447
		GO:0043632 modification-dependent macromolecule catabolic process	23	6.990881	0.004744	7.361701
		GO:0040029 regulation of gene expression, epigenetic	10	3.039514	0.004988	7.725257
		GO:0009057 macromolecule catabolic process	34	10.33435	0.009079	13.64087
		GO:0001701 in utero embryonic development	4	1.215805	0.010359	15.41676
		GO:0008380 RNA splicing	17	5.167173	0.011628	17.14332

		GO:0006508 proteolysis	31	9.422492	0.01331	19.38243
		GO:0000377 RNA splicing, via transesterification reactions with bulged adenosine as nucleophile	12	3.647416	0.014472	20.89522
		GO:0000398 nuclear mRNA splicing, via spliceosome	12	3.647416	0.014472	20.89522
		GO:0000375 RNA splicing, via transesterification reactions	12	3.647416	0.014472	20.89522
		GO:0022402 cell cycle process	31	9.422492	0.018971	26.50677
		GO:0031145 anaphase-promoting complex-dependent proteasomal ubiquitin-dependent protein catabolic process	4	1.215805	0.019085	26.64397
		GO:0006511 ubiquitin-dependent protein catabolic process	15	4.559271	0.022913	31.11345
		GO:0045814 negative regulation of gene expression, epigenetic	7	2.12766	0.024434	32.81806
		GO:0006342 chromatin silencing	7	2.12766	0.024434	32.81806
		GO:0001824 blastocyst development	3	0.911854	0.031963	40.6868
		GO:0016458 gene silencing	9	2.735562	0.038398	46.71852
		GO:0048589 developmental growth	4	1.215805	0.045416	52.63866
		GO:0045005 maintenance of fidelity during DNA-dependent DNA replication	4	1.215805	0.045416	52.63866
		GO:0051329 interphase of mitotic cell cycle	8	2.431611	0.048543	55.07206
		GO:0051325 interphase	8	2.431611	0.048543	55.07206
		GO:0055114 oxidation reduction	50	24.87562	1.92E-08	2.90E-05
		GO:0019439 aromatic compound catabolic process	9	4.477612	6.51E-06	0.009846
		GO:0009072 aromatic amino acid family metabolic process	6	2.985075	0.005893	8.555429
		GO:0006559 L-phenylalanine catabolic process	3	1.492537	0.011943	16.62402
		GO:0006743 ubiquinone metabolic process	4	1.99005	0.02274	29.39559
Brown	255	GO:0006744 ubiquinone biosynthetic process	4	1.99005	0.02274	29.39559
		GO:0006570 tyrosine metabolic process	3	1.492537	0.022873	29.54075
		GO:0042375 quinone cofactor metabolic process	4	1.99005	0.02981	36.74123
		GO:0045426 quinone cofactor biosynthetic process	4	1.99005	0.02981	36.74123
		GO:0009074 aromatic amino acid family catabolic process	3	1.492537	0.036514	43.04263
		GO:0006558 L-phenylalanine metabolic process	3	1.492537	0.036514	43.04263

		GO:0055085 transmembrane transport	13	12.26415	0.001177	1.674974
		GO:0019725 cellular homeostasis	7	6.603774	0.018404	23.39486
		GO:0032324 molybdopterin cofactor biosynthetic process	3	2.830189	0.034316	39.40631
		GO:0019720 Mo-molybdopterin cofactor metabolic process	3	2.830189	0.034316	39.40631
Cyan	122	GO:0043545 molybdopterin cofactor metabolic process	3	2.830189	0.034316	39.40631
		GO:0006777 Mo-molybdopterin cofactor biosynthetic process	3	2.830189	0.034316	39.40631
		GO:0051189 prosthetic group metabolic process	3	2.830189	0.034316	39.40631
		GO:0030003 cellular cation homeostasis	5	4.716981	0.041285	45.38691
		GO:0055080 cation homeostasis	5	4.716981	0.047457	50.21968
Darkgreen	38	N/A				
Darkgrey	34	N/A				
Darkmagenta	14	N/A				
Darkolivegreen	14	N/A				
		GO:0032509 endosome transport via multivesicular body sorting pathway	2	6.666667	0.038998	39.00586
Darkorange	31	GO:0032511 late endosome to vacuole transport via multivesicular body sorting pathway	2	6.666667	0.038998	39.00586
		GO:0045324 late endosome to vacuole transport	2	6.666667	0.048516	46.10372
Darkred	41	N/A				
		GO:0006413 translational initiation	3	8.823529	0.028495	31.21014
Darkturquoise	38	GO:0030005 cellular di-, tri-valent inorganic cation homeostasis	3	8.823529	0.038901	40.15916
		GO:0055066 di-, tri-valent inorganic cation homeostasis	3	8.823529	0.041699	42.37462
Green	227	GO:0055114 oxidation reduction	44	23.28042	1.96E-06	0.002969
		GO:0008652 cellular amino acid biosynthetic process	16	8.465608	2.90E-04	0.438221

		GO:0009309 amine biosynthetic process	17	8.994709	5.84E-04	0.87913
		GO:0016053 organic acid biosynthetic process	18	9.52381	0.001905	2.841252
		GO:0046394 carboxylic acid biosynthetic process	18	9.52381	0.001905	2.841252
		GO:0006790 sulfur metabolic process	11	5.820106	0.003074	4.547318
		GO:0009069 serine family amino acid metabolic process	7	3.703704	0.004995	7.290897
		GO:0006555 methionine metabolic process	6	3.174603	0.0191	25.28927
		GO:0044271 nitrogen compound biosynthetic process	22	11.64021	0.019555	25.81152
		GO:0000103 sulfate assimilation	3	1.587302	0.020853	27.28196
		GO:0009086 methionine biosynthetic process	5	2.645503	0.026001	32.85196
		GO:0044272 sulfur compound biosynthetic process	7	3.703704	0.026591	33.46477
		GO:0009067 aspartate family amino acid biosynthetic process	6	3.174603	0.033944	40.67072
		GO:0019318 hexose metabolic process	8	4.232804	0.034543	41.2245
		GO:0043623 cellular protein complex assembly	9	5.521472	0.003539	5.407676
		GO:0070271 protein complex biogenesis	12	7.361963	0.004907	7.422726
Greenyellow	197	GO:0006461 protein complex assembly	12	7.361963	0.004907	7.422726
		GO:0065003 macromolecular complex assembly	13	7.97546	0.032037	39.98295
		GO:0034622 cellular macromolecular complex assembly	10	6.134969	0.04839	54.05356
Grey60	71	GO:0019673 GDP-mannose metabolic process	2	3.225806	0.038429	40.98007
		GO:0007264 small GTPase mediated signal transduction	5	5.376344	0.020635	26.1079
Lightcyan	108	GO:0006271 DNA strand elongation during DNA replication	3	3.225806	0.040887	45.43458
		GO:0022616 DNA strand elongation	3	3.225806	0.040887	45.43458
Lightgreen	64	N/A				
Lightyellow	49	GO:0055114 oxidation reduction	10	23.25581	0.046411	48.51141
Magenta	207	GO:0044264 cellular polysaccharide metabolic process	6	3.726708	0.009389	13.51886

		GO:0031125 rRNA 3'-end processing	3	1.863354	0.023965	31.16685
		GO:0042592 homeostatic process	11	6.832298	0.024514	31.76089
		GO:0019725 cellular homeostasis	9	5.590062	0.028139	35.56231
		GO:0055085 transmembrane transport	14	8.695652	0.037541	44.52064
		GO:0055066 di-, tri-valent inorganic cation homeostasis	5	3.10559	0.047642	52.83951
		GO:0006493 protein amino acid O-linked glycosylation	3	3.488372	0.007012	9.549232
		GO:0055114 oxidation reduction	18	20.93023	0.010308	13.73726
		GO:0009101 glycoprotein biosynthetic process	4	4.651163	0.032613	37.67952
Midnightblue	108	GO:0006486 protein amino acid glycosylation	4	4.651163	0.032613	37.67952
		GO:0070085 glycosylation	4	4.651163	0.032613	37.67952
		GO:0043413 biopolymer glycosylation	4	4.651163	0.032613	37.67952
		GO:0009100 glycoprotein metabolic process	4	4.651163	0.043247	46.76924
Orange	32	N/A				
paleturquoise	20	GO:0033554 cellular response to stress	6	33.33333	0.01611	16.96155
		GO:0055114 oxidation reduction	29	16.86047	0.010993	15.56778
		GO:0006534 cysteine metabolic process	4	2.325581	0.033641	40.77873
		GO:0006790 sulfur metabolic process	8	4.651163	0.035403	42.41032
Pink	210	GO:0006541 glutamine metabolic process	4	2.325581	0.040475	46.8757
		GO:0010948 negative regulation of cell cycle process	4	2.325581	0.047953	52.87248
		GO:0006553 lysine metabolic process	3	1.744186	0.048283	53.12153
		GO:0046677 response to antibiotic	3	1.764706	0.024301	31.41688
		GO:0006259 DNA metabolic process	16	9.411765	0.028379	35.6824
Purple	205	GO:0006913 nucleocytoplasmic transport	8	4.705882	0.031545	38.82124
		GO:0051169 nuclear transport	8	4.705882	0.034209	41.35087
		GO:0006260 DNA replication	9	5.294118	0.036364	43.32523

		GO:0006406 mRNA export from nucleus	5	2.941176	0.038043	44.82033
		GO:0044087 regulation of cellular component biogenesis	5	2.941176	0.0432	49.18519
		GO:0006405 RNA export from nucleus	5	2.941176	0.0432	49.18519
		GO:0010604 positive regulation of macromolecule metabolic process	6	3.529412	0.044859	50.51896
Red	224	GO:0015672 monovalent inorganic cation transport	8	4.188482	9.56E-04	1.499417
		GO:0015992 proton transport	5	2.617801	0.021981	29.61657
		GO:0006818 hydrogen transport	5	2.617801	0.026531	34.6162
		GO:0015837 amine transport	6	3.141361	0.030423	38.62728
		GO:0006814 sodium ion transport	3	1.570681	0.048582	54.47691
Royalblue	48	N/A				
Saddlebrown	23	N/A				
Salmon	147	GO:0019722 calcium-mediated signaling	3	2.380952	0.01278	17.04662
		GO:0055085 transmembrane transport	11	8.730159	0.044356	48.27387
Sienna3	13	N/A				
Skyblue	23	GO:0055085 transmembrane transport	4	20	0.047487	43.7957
Skyblue3	11	N/A				
Steelblue	20	N/A				
Tan	196	GO:0015711 organic anion transport	3	2.054795	0.017979	24.28349
		GO:0007568 aging	4	2.739726	0.026418	33.66834
		GO:0007264 small GTPase mediated signal transduction	6	4.109589	0.027478	34.76623
		GO:0045859 regulation of protein kinase activity	4	2.739726	0.049471	54.06332

Turquoise	711	GO:0006350 transcription	59	9.899329	0.002969	4.867518
		GO:0006360 transcription from RNA polymerase I promoter	8	1.342282	0.009606	14.95274
		GO:0045449 regulation of transcription	56	9.395973	0.013613	20.54556
		GO:0006323 DNA packaging	11	1.845638	0.013651	20.59699
		GO:0010608 posttranscriptional regulation of gene expression	17	2.852349	0.022562	31.81398
		GO:0010629 negative regulation of gene expression	19	3.187919	0.030992	41.03813
		GO:0040029 regulation of gene expression, epigenetic	12	2.013423	0.032717	42.774
		GO:0006333 chromatin assembly or disassembly	9	1.510067	0.035944	45.89468
		GO:0016458 gene silencing	13	2.181208	0.040975	50.44296
		GO:0040008 regulation of growth	9	1.510067	0.047219	55.58722
Violet	16	GO:0008202 steroid metabolic process	3	21.42857	0.006521	5.810298
		GO:0016128 phytosteroid metabolic process	2	14.28571	0.043699	33.55522
		GO:0008204 ergosterol metabolic process	2	14.28571	0.043699	33.55522
White	30	GO:0006873 cellular ion homeostasis	3	11.11111	0.048453	46.02995
		GO:0055082 cellular chemical homeostasis	3	11.11111	0.048453	46.02995
Yellow	244	GO:0045184 establishment of protein localization	24	11.76471	0.001699	2.534166
		GO:0008104 protein localization	26	12.7451	0.003171	4.68049
		GO:0015031 protein transport	23	11.27451	0.003354	4.943299
		GO:0006631 fatty acid metabolic process	9	4.411765	0.021577	28.05075
Yellowgreen	11	N/A				

Supplementary Table 7. Protein information in brown module.

Protein ID	Protein name	Connections
comp10018_c0_seq1	Diacetyl reductase [(S)-acetoin forming]	1010
comp10067_c0_seq1	Iron transport multicopper oxidase FET3	1057
comp10082_c0_seq1	Acyl-CoA desaturase	1010
comp10200_c0_seq1	N-chimaerin	2004
comp10420_c0_seq1	Sorbitol dehydrogenase	1039
comp10434_c0_seq1	Uncharacterized methyltransferase C1347.09	1010
comp1049_c0_seq1	UPF0357 protein C1687.07	1010
comp105_c0_seq1	U4/U6 small nuclear ribonucleoprotein Prp31	1825
comp10785_c0_seq1	Elongation factor 2	1010
comp10849_c0_seq1	Geranylgeranyl transferase type-2 subunit alpha	1010
comp10900_c0_seq1	Cofilin	1419
comp10990_c0_seq1	Putative epoxide hydrolase	1913
comp10992_c0_seq1	Putative epoxide hydrolase	2092
comp11005_c0_seq1	Mitochondrial import inner membrane translocase subunit TIM10	1280
comp11010_c0_seq1	Putative band 7 family protein R614	1430
comp11027_c0_seq1	Ubiquitin-conjugating enzyme spm2	1331
comp11043_c0_seq1	Tubulin beta-1 chain	1781
comp11056_c0_seq1	24 kDa Ras-like protein	1571
comp1106_c0_seq1	L-2,3-butanediol dehydrogenase	2016
comp11087_c0_seq1	Uncharacterized oxidoreductase C663.06c	1534
comp11099_c0_seq1	Methylsterol monooxygenase	1568
comp11103_c0_seq1	ATP-citrate synthase	1258
comp11107_c0_seq1	Serine/threonine-protein kinase hal4	1440
comp11108_c0_seq1	Peroxidase 2	1333
comp11130_c0_seq1	Phenylalanine ammonia-lyase	996
comp1114_c0_seq1	Casein kinase I isoform delta-like	890
comp11172_c0_seq1	NADH dehydrogenase [ubiquinone] iron-sulfur protein 5-B	929

comp11208_c0_seq1	V-type proton ATPase subunit F	1804
comp11224_c0_seq1	Cysteine proteinase 1, mitochondrial	1725
comp1124_c0_seq1	Ubiquitin-conjugating enzyme E2-16 kDa	1308
comp11269_c0_seq1	54S ribosomal protein L38, mitochondrial	1010
comp11284_c0_seq1	Probable succinate dehydrogenase [ubiquinone] flavoprotein subunit, mitochondrial	1736
comp1129_c0_seq1	Uncharacterized RING finger protein P8B7.15c	1296
comp11306_c0_seq1	Endonuclease III homolog	1010
comp11308_c0_seq1	Thioredoxin-like protein 4A	2031
comp11309_c0_seq1	Mannosyl phosphorylinositol ceramide synthase SUR1	1411
comp1135_c0_seq1	Peroxiredoxin HYR1	1356
comp11389_c0_seq1	Signal peptidase complex catalytic subunit SEC11	1396
comp11390_c0_seq1	Purine-cytosine permease fcyB	1010
comp11405_c0_seq1	Cyanate hydratase	1251
comp11421_c0_seq1	N/A	1974
comp11453_c0_seq1	Mitochondrial intermembrane space cysteine motif-containing protein MIC17	1010
comp11455_c0_seq1	Glutathione S-transferase PM239X14	1362
comp11546_c0_seq1	Probable glycosidase C21B10.07	1010
comp11548_c0_seq1	Transmembrane protein 184 homolog C30D11.06c	1716
comp11577_c0_seq1	Dolichyl-diphosphooligosaccharide--protein glycosyltransferase subunit 1	1305
comp11612_c0_seq1	Diacetyl reductase [(S)-acetoin forming]	1915
comp11616_c0_seq1	Peroxisomal targeting signal receptor	1640
comp11645_c0_seq1	Eukaryotic translation initiation factor 4E-1	1603
comp1166_c0_seq1	Meiotic expression up-regulated protein 26	1238
comp11664_c0_seq1	Cohesin subunit rad21	1447
comp11666_c0_seq1	Putative xanthine/uracil permease C887.17	955
comp11696_c0_seq1	Probable ATP-dependent permease	1242
comp11704_c0_seq1	Probable aspartic-type endopeptidase AFUA_3G01220	1635
comp11736_c0_seq1	Cytosolic Fe-S cluster assembly factor NAR1	1225
comp11763_c0_seq1	Carboxypeptidase Y homolog A	1790
comp11781_c0_seq1	Nitrogen regulatory protein areA	1567

comp11822_c0_seq1	Transport protein particle subunit trs31	1376
comp1199_c0_seq1	GTP cyclohydrolase 1	1010
comp1273_c0_seq1	Urease	1072
comp1296_c0_seq1	Chitinase 1	1010
comp1346_c0_seq1	Uncharacterized MFS-type transporter C1683.03c	1044
comp1364_c0_seq1	Putative epoxide hydrolase	1459
comp1376_c0_seq1	Protein disulfide-isomerase	1841
comp1434_c0_seq1	Endoglucanase EG-II	1010
comp1480_c0_seq1	Probable serine/threonine-protein kinase drkD	1010
comp1503_c0_seq1	Autophagy-related protein 8	2077
comp1520_c0_seq1	NADP-dependent alcohol dehydrogenase 6	1532
comp1531_c0_seq1	Probable prephenate dehydrogenase [NADP(+)]	1768
comp1622_c0_seq1	Thioredoxin reductase	1665
comp1631_c0_seq1	Uncharacterized methyltransferase C1B3.06c	1516
comp1650_c0_seq1	Nitrate reductase [NADPH]	1493
comp1651_c0_seq1	Homogentisate 1,2-dioxygenase	2003
comp166_c0_seq1	GTP-binding protein 1 (Fragment)	1158
comp1692_c0_seq1	Glutathione S-transferase 2	1991
comp1749_c0_seq1	Homologous-pairing protein 2	1010
comp1770_c0_seq1	6-hydroxy-D-nicotine oxidase	1622
comp1859_c0_seq1	NADH dehydrogenase [ubiquinone] complex I, assembly factor 7	1010
comp1963_c0_seq1	Low-affinity potassium transport protein	1469
comp1998_c0_seq1	N-lysine methyltransferase see1	1010
comp200_c0_seq1	Uncharacterized transporter C11D3.18C	1010
comp2012_c0_seq1	Postreplication repair E3 ubiquitin-protein ligase rad18	1010
comp2024_c0_seq1	E3 ubiquitin-protein ligase RNF220	1553
comp2037_c0_seq1	Golgi apparatus membrane protein TVP38	1562
comp2041_c0_seq1	Thioredoxin domain-containing protein C13F5.05, mitochondrial	1270
comp2072_c0_seq1	Succinate dehydrogenase [ubiquinone] iron-sulfur subunit, mitochondrial	1406
comp2076_c0_seq1	NAD/NADP-dependent indole-3-acetaldehyde reductase	1557

comp2095_c0_seq1	Peptidyl-Lys metalloendopeptidase	1010
comp2113_c0_seq1	Potassium voltage-gated channel subfamily B member 2	1010
comp2121_c0_seq1	Uncharacterized oxidoreductase YMR226C	1094
comp2146_c0_seq1	Uncharacterized oxidoreductase YusZ	1181
comp2157_c0_seq1	N/A	1710
comp2187_c0_seq1	Cytochrome b-c1 complex subunit Rieske, mitochondrial	1305
comp223_c0_seq1	Aryl-alcohol dehydrogenase [NADP(+)]	1475
comp2252_c0_seq1	Probable amino-acid permease PB1C11.02	1615
comp2295_c0_seq1	Lipase 4	1010
comp231_c0_seq1	L-sorbose 1-dehydrogenase	1010
comp232_c0_seq1	Quinone oxidoreductase PIG3	1562
comp2338_c0_seq1	Serine/threonine/tyrosine-interacting protein	1599
comp2345_c0_seq1	Small glutamine-rich tetratricopeptide repeat-containing protein	1010
comp2355_c0_seq1	Aldo-keto reductase yakc [NADP(+)]	1595
comp2385_c0_seq1	Protein SKT5	1927
comp2452_c0_seq1	Histidine-rich membrane protein KE4 homolog 1	1010
comp2458_c0_seq1	Dimethyladenosine transferase	1010
comp2499_c0_seq1	Translation initiation factor IF-2	1494
comp2569_c0_seq1	Manganese peroxidase 3	1168
comp2570_c0_seq1	Manganese peroxidase 3	1010
comp2627_c0_seq1	Mitotic spindle assembly checkpoint protein MAD2B	1010
comp2644_c0_seq1	Lipase 1	1010
comp2645_c0_seq1	Lipase 1	1496
comp2650_c0_seq1	Serine--tRNA ligase	1010
comp2665_c0_seq1	Protein CGI121	1755
comp2678_c0_seq1	Serine/threonine-protein kinase 32A	2049
comp269_c0_seq1	Alpha-aminoadipic semialdehyde synthase, mitochondrial	1582
comp2691_c0_seq1	Cytochrome c oxidase subunit 6, mitochondrial	1736
comp2706_c0_seq1	Zinc phosphodiesterase ELAC protein 1	1004
comp2714_c0_seq1	Glycerol-3-phosphate O-acyltransferase 1	1609

comp2744_c0_seq1	Transcription initiation factor IIA large subunit	1010
comp276_c0_seq1	NAD/NADP-dependent indole-3-acetaldehyde reductase	1785
comp2762_c0_seq1	Carboxypeptidase S	1382
comp2775_c0_seq1	Very-long-chain 3-oxoacyl-CoA reductase	1011
comp2777_c0_seq1	Putative reductase 1	1010
comp2782_c0_seq1	ABC transporter ATP-binding protein/permease VMR1	1432
comp2798_c0_seq1	Aldo-keto reductase yakc [NADP(+)]	1595
comp2804_c0_seq1	U3 small nucleolar RNA-associated protein 14 homolog C	1960
comp2820_c0_seq1	Heat shock 70 kDa protein cognate 1	1010
comp2845_c0_seq1	General negative regulator of transcription subunit 2	2069
comp2912_c0_seq1	RNA polymerase II transcription factor B subunit 4	1010
comp2938_c0_seq1	DNA-directed RNA polymerase III subunit rpc3	1352
comp2949_c0_seq1	Transforming growth factor beta regulator 1	1760
comp2959_c0_seq1	Amino-acid permease BAT1	1533
comp297_c0_seq1	Putative aryl-alcohol dehydrogenase C977.14c	1648
comp2974_c0_seq1	O-methylsterigmatocystin oxidoreductase	1991
comp2999_c0_seq1	Peroxisomal membrane protein LPX1	1491
comp3034_c0_seq1	Dolichyl-phosphate beta-glucosyltransferase	1357
comp3035_c0_seq1	Extracellular metalloproteinase MEP	1431
comp3075_c0_seq1	OTU domain-containing protein 3	1010
comp3078_c0_seq1	Zinc-binding alcohol dehydrogenase domain-containing protein cipB	1419
comp3093_c0_seq1	Guanine nucleotide-binding protein alpha-3 subunit	980
comp3146_c0_seq1	Uncharacterized MscS family protein C1183.11	2094
comp3187_c0_seq1	78 kDa glucose-regulated protein homolog	1408
comp327_c0_seq1	Manganese peroxidase 3	1010
comp328_c0_seq1	Phenol 2-monooxygenase	990
comp3325_c0_seq1	Probable anthranilate synthase component 1	1897
comp337_c0_seq1	3-ketoacyl-CoA thiolase, peroxisomal	2085
comp3424_c0_seq1	Protein YIPF5 homolog	1010
comp344_c0_seq1	DNA mismatch repair protein msh6	1074

comp3443_c0_seq1	Ubiquinone biosynthesis protein COQ4, mitochondrial	1010
comp3527_c0_seq1	Uncharacterized oxidoreductase C736.13	1010
comp3559_c0_seq1	Werner syndrome ATP-dependent helicase homolog	2160
comp358_c0_seq1	Ribonuclease T2	1010
comp3593_c0_seq1	Cytidine deaminase	1619
comp3597_c0_seq1	Lipid A export ATP-binding/permease protein MsbA	1298
comp3635_c0_seq1	Acyl-coenzyme A:6-aminopenicillanic-acid-acyltransferase 40 kDa form	1653
comp3637_c0_seq1	CaiB/baiF CoA-transferase family protein C7orf10	1010
comp3643_c0_seq1	Lysine-specific permease	2124
comp3702_c0_seq1	Uncharacterized oxidoreductase C736.13	1010
comp3856_c0_seq1	D-lactate dehydrogenase [cytochrome], mitochondrial	1512
comp3909_c0_seq1	TIP41-like protein	1010
comp3938_c0_seq1	Glucan endo-1,3-alpha-glucosidase agn1	1010
comp395_c0_seq1	Putative epoxide hydrolase	1591
comp4013_c0_seq1	PHO85 cyclin-9	1010
comp4025_c0_seq1	Serine/threonine-protein kinase pakC	1454
comp416_c0_seq1	Uncharacterized transporter YIL166C	1010
comp4174_c0_seq1	Phosphatidylinositide phosphatase SAC2	1968
comp4191_c0_seq1	Mitogen-activated protein kinase 1	1974
comp4226_c0_seq1	NADPH-dependent methylglyoxal reductase GRE2	1352
comp4235_c0_seq1	Putative NADPH-dependent methylglyoxal reductase GRP2	1010
comp4309_c0_seq1	Putative carboxymethylenebutenolidase	1613
comp4333_c0_seq1	Zinc-type alcohol dehydrogenase-like protein C2E1P3.01	1769
comp4360_c0_seq1	tRNA-specific 2-thiouridylase MnmA	1010
comp4459_c0_seq1	Oxalate decarboxylase OxdC	1570
comp4482_c0_seq1	Sugar phosphatase YfbT	1010
comp4485_c0_seq1	Choline dehydrogenase	2032
comp4503_c0_seq1	Thiamine thiazole synthase	1666
comp454_c0_seq1	Porphobilinogen deaminase	1387
comp455_c0_seq1	N/A	1308

comp4584_c0_seq1	Inositol oxygenase	1010
comp4631_c0_seq1	Tryptophan synthase	1135
comp4676_c0_seq1	Peroxiredoxin-6	1939
comp4685_c0_seq1	DNA repair and recombination protein pif1, mitochondrial	1370
comp4764_c0_seq1	Aquaporin-2	1010
comp478_c0_seq1	4-hydroxybenzoate polyprenyltransferase, mitochondrial	1010
comp4834_c0_seq1	Putative sterigmatocystin biosynthesis monooxygenase stcW	1010
comp5009_c0_seq1	Mitochondrial substrate carrier family protein G	1010
comp5144_c0_seq1	ATP-dependent DNA helicase hus2/rqh1	1241
comp5198_c0_seq1	Salicylate hydroxylase	1010
comp5212_c0_seq1	Vacuolar protein sorting-associated protein 74	1716
comp5239_c0_seq1	Homogentisate 1,2-dioxygenase	1571
comp5246_c0_seq1	Homogentisate 1,2-dioxygenase	1584
comp5320_c0_seq1	Phosphatidylglycerol/phosphatidylinositol transfer protein	1381
comp5369_c0_seq1	Chitin biosynthesis protein CHS5	1352
comp5374_c0_seq1	Rho GTPase-activating protein 39	1599
comp540_c0_seq1	1,4-alpha-glucan-branching enzyme	789
comp5482_c0_seq1	Glutathione S-transferase PM239X14	1010
comp552_c0_seq1	Ras-related GTP-binding protein A	1010
comp5681_c0_seq1	Vanillin dehydrogenase	1218
comp5683_c0_seq1	Putative aryl-alcohol dehydrogenase C977.14c	1124
comp5731_c0_seq1	Protein N-lysine methyltransferase METTL21A	1010
comp5759_c0_seq1	UDP-N-acetylglucosamine transferase subunit ALG13 homolog	1080
comp5810_c0_seq1	Uncharacterized amino-acid permease C584.13	1024
comp5901_c0_seq1	Quinone-oxidoreductase homolog, chloroplastic	1256
comp5953_c0_seq1	Translation initiation factor eIF-2B subunit alpha	1659
comp596_c0_seq1	Versatile peroxidase VPL1	1304
comp6043_c0_seq1	Putative ATP-dependent helicase hrq1	1010
comp6193_c0_seq1	Alpha-ketoglutarate-dependent sulfonate dioxygenase	1163
comp6210_c0_seq1	L-fuconate dehydratase	1532

comp6254_c0_seq1	Protein ABC1 homolog, mitochondrial	1177
comp6282_c0_seq1	Uncharacterized N-acetyltransferase p20	1010
comp6340_c0_seq1	Cytosolic Fe-S cluster assembly factor CFD1	1010
comp6342_c0_seq1	Cytosolic Fe-S cluster assembly factor CFD1	1253
comp6357_c0_seq1	Major facilitator superfamily domain-containing protein 10	1105
comp6358_c0_seq1	Major facilitator superfamily domain-containing protein 10	1370
comp640_c0_seq1	Probable drug/proton antiporter YHK8	1700
comp6414_c0_seq1	Putative agmatinase 1	1010
comp6525_c0_seq1	Oxalate decarboxylase OxdC	1010
comp661_c0_seq1	WW domain-containing oxidoreductase	1902
comp668_c0_seq1	Aldehyde dehydrogenase family 3 member H1	1089
comp6727_c0_seq1	Translin-associated protein X	1010
comp6728_c0_seq1	Alpha-aminoadipic semialdehyde dehydrogenase	1137
comp6729_c0_seq1	Alpha-aminoadipic semialdehyde dehydrogenase	1137
comp6738_c0_seq1	Uncharacterized transporter C11D3.06	1010
comp6931_c0_seq1	Copper resistance protein CRF1	1010
comp7051_c0_seq1	Formamidase	1335
comp7058_c0_seq1	Formamidase	1001
comp7084_c0_seq1	Inositol polyphosphate 5-phosphatase OCRL-1	1552
comp712_c0_seq1	Probable lipase C1672.09	1321
comp7161_c0_seq1	Salicylate hydroxylase	1912
comp7230_c0_seq1	2-alkenal reductase (NADP(+)-dependent)	1010
comp724_c0_seq1	Peptide chain release factor 1	1099
comp7357_c0_seq1	Probable endo-1,3(4)-beta-glucanase An02g00850	1010
comp7446_c0_seq1	O-methylsterigmatocystin oxidoreductase	1662
comp751_c0_seq1	Serine/threonine-protein kinase MRCK alpha	1010
comp7561_c0_seq1	Coiled-coil domain-containing protein 174	1010
comp7711_c0_seq1	Meiotically up-regulated gene 190 protein	1927
comp7712_c0_seq1	Calcium uniporter protein, mitochondrial	1010
comp7833_c0_seq1	UPF0695 membrane protein YOR390W	1010

comp7837_c0_seq1	Uncharacterized protein C119.09c	1118
comp7893_c0_seq1	Dynactin subunit 6	1010
comp8111_c0_seq1	Uncharacterized RWD, RING finger and WD repeat-containing protein C11E3.05	1252
comp814_c0_seq1	Cyclopropane-fatty-acyl-phospholipid synthase	1010
comp8242_c0_seq1	WD repeat-containing protein JIP5	1790
comp8361_c0_seq1	Spermatogenesis-associated protein 20	1576
comp868_c0_seq1	Dolichyl-phosphate-mannose--protein mannosyltransferase 4	1332
comp876_c0_seq1	Calcium-transporting ATPase sarcoplasmic/endoplasmic reticulum type	1675
comp877_c0_seq1	Uncharacterized ABC transporter ATP-binding protein C20G4.01	1593
comp88_c0_seq1	External alternative NAD(P)H-ubiquinone oxidoreductase B1, mitochondrial	1385
comp921_c0_seq1	N/A	1408
comp926_c0_seq1	Ribosome production factor 1	1751
comp9308_c0_seq1	Pyrimidine-specific ribonucleoside hydrolase RihA	1637
comp9309_c0_seq1	Putative oxidoreductase bli-4, mitochondrial	1010
comp961_c0_seq1	Pumilio domain-containing protein C6G9.14	1894
comp9647_c0_seq1	Probable glycosidase C21B10.07	1010
comp9941_c0_seq1	O-methylsterigmatocystin oxidoreductase	1010
comp998_c0_seq1	Putative sterigmatocystin biosynthesis monooxygenase stcW	1040

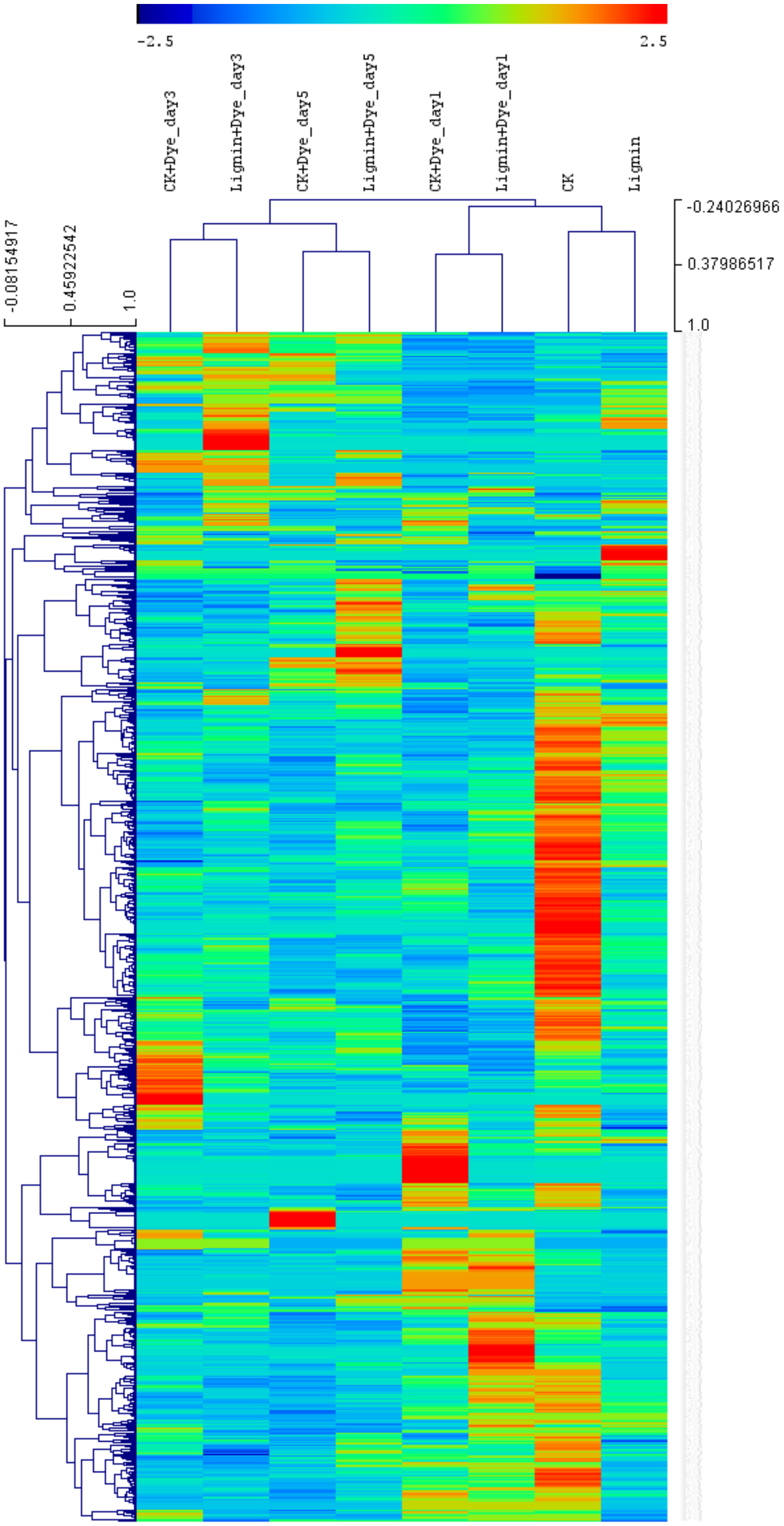

Figure S1. Hierarchical cluster overview of the total 896 differentially expressed proteins among the different time course and conditions. The detailed protein information and abundance in this heat map was listed in Supplementary Table 3.
